# Transcriptional and Proteomic Responses to Carbon Starvation in *Paracoccidioides*

**DOI:** 10.1371/journal.pntd.0002855

**Published:** 2014-05-08

**Authors:** Patrícia de Sousa Lima, Luciana Casaletti, Alexandre Melo Bailão, Ana Tereza Ribeiro de Vasconcelos, Gabriel da Rocha Fernandes, Célia Maria de Almeida Soares

**Affiliations:** 1 Laboratório de Biologia Molecular, Instituto de Ciências Biológicas, Universidade Federal de Goiás, Goiânia, Goiás, Brazil; 2 Programa de Pós Graduação em Patologia Molecular, Faculdade de Medicina, Universidade de Brasília, Brasília, Distrito Federal, Brazil; 3 Laboratório Nacional de Computação Científica (LNCC), Petrópolis, Rio de Janeiro, Brazil; 4 Laboratório de Bioinformática, Universidade Católica de Brasília, Brasília, Distrito Federal, Brazil; University of California San Diego School of Medicine, United States of America

## Abstract

**Background:**

The genus *Paracoccidioides* comprises human thermal dimorphic fungi, which cause paracoccidioidomycosis (PCM), an important mycosis in Latin America. Adaptation to environmental conditions is key to fungal survival during human host infection. The adaptability of carbon metabolism is a vital fitness attribute during pathogenesis.

**Methodology/Principal Findings:**

The fungal pathogen *Paracoccidioides* spp. is exposed to numerous adverse conditions, such as nutrient deprivation, in the human host. In this study, a comprehensive response of *Paracoccidioides*, *Pb*01, under carbon starvation was investigated using high-resolution transcriptomic (RNAseq) and proteomic (NanoUPLC-MS^E^) approaches. A total of 1,063 transcripts and 421 proteins were differentially regulated, providing a global view of metabolic reprogramming during carbon starvation. The main changes were those related to cells shifting to gluconeogenesis and ethanol production, supported by the degradation of amino acids and fatty acids and by the modulation of the glyoxylate and tricarboxylic cycles. This proposed carbon flow hypothesis was supported by gene and protein expression profiles assessed using qRT-PCR and western blot analysis, respectively, as well as using enzymatic, cell dry weight and fungus-macrophage interaction assays. The carbon source provides a survival advantage to *Paracoccidioides* inside macrophages.

**Conclusions/Significance:**

For a complete understanding of the physiological processes in an organism, the integration of approaches addressing different levels of regulation is important. To the best of our knowledge, this report presents the first description of the responses of *Paracoccidioides* spp. to host-like conditions using large-scale expression approaches. The alternative metabolic pathways that could be adopted by the organism during carbon starvation can be important for a better understanding of the fungal adaptation to the host, because systems for detecting and responding to carbon sources play a major role in adaptation and persistence in the host niche.

## Author Summary

The species of the *Paracoccidioides* genus, a neglected human pathogen, represent the causative agents of paracoccidioidomycosis (PCM), one of the most frequent systemic mycoses in Latin America. Despite being phagocytosed, the fungus conidia differentiate into the parasitic yeast form that subverts the normally harsh intraphagosomal environment and survives and replicates into murine and human macrophages. It has been suggested that alternative carbon metabolism plays a role in the survival and virulence of *Paracoccidioides* spp. within host cells. We used large-scale transcriptome and proteome approaches to better characterize the responses of *Paracoccidioides, Pb*01, yeast parasitic cells, to carbon starvation. We aimed to identify important molecules used by the fungus to adapt to these hostile conditions. The shift to a starvation mode, including gluconeogenesis and ethanol increases, activation of fatty acids, and amino acid degradation are the strategies used by the pathogen to persist under this stress. Our study provides a detailed map of *Paracoccidioides* spp. responses under carbon starvation conditions and contributes to further investigations of the importance of alternative carbon adaptation during fungus pathogenesis.

## Introduction

Metabolic adaptability and flexibility are important attributes for pathogens to successfully colonize, infect, and cause disease in a wide range of hosts. Therefore, they must be able to assimilate various carbon sources. Carbohydrates are the primary and preferred source of metabolic carbon for most organisms and are used for generating energy and producing biomolecules [1].

Studies have highlighted the importance of carbon metabolism in fungi [2], [3]. Pathogens such as *Candida albicans* display sufficient metabolic flexibility to assimilate the available nutrients in diverse niches such as the skin, mucous membranes, blood, and biofilms [4], [5]. The mucosal surface of the lung may provide a more nutrient-limited condition because it is not in direct contact with nutrients from food intake [6]. Additionally, in the lungs, macrophages rapidly phagocytize inhaled microorganisms supported by neutrophils and dendritic cells [7]. Macrophages are considered a glucose- and amino acid-poor environment [8], [9] and may form extremely nutrient-limited conditions causing severe starvation [10]. In *C. albicans* and *Cryptococcus neoformans*, alternative carbon metabolism was detected after internalization by macrophages playing a role in fungal survival in these host cells [9], [11], [12]. In contrast, in the case of systemic infections, pathogens can reach different internal organs such as the liver, which is the main storage compartment of glucose in the form of glycogen. The bloodstream, for example, is the major carrier of nutrients, glucose, proteins, amino acids, and vitamins in larger quantities [10]. In this way, metabolic and stress adaptation represent vital fitness attributes that have evolved alongside virulence attributes in fungi [8]–[10], [13].

Alternative carbon metabolism was also described to be important to protozoa and bacteria [14], [15]. *Entamoeba histolytica* uses an alternative source of energy when the microorganism is exposed to glucose starvation. In the specific pyruvate-to-ethanol pathway in *E. histolytica*, acetyl-CoA is converted to acetaldehyde, which is then reduced to ethanol [16]. Reduced levels of the long-chain fatty-acid-CoA ligase protein during glucose starvation conditions in *E. histolytica* may explain a mechanism by which acetyl-CoA is shuttled from the fatty acid metabolism into this pyruvate-to-ethanol pathway. In addition, the glucose starvation modulates the protozoa virulence, based on proteome analysis [15]. The transcriptome and large-scale proteome dynamics were also analyzed in *Bacillus subtilis* from glucose-starved cells. A direct consequence of glucose depletion on proteins was the switch from glycolytic to gluconeogenic metabolism and elevated abundance of proteins of the tricarboxylic cycle used for energy generation. Genes that are involved in exponential growth, amino-acid biosynthesis, purine/pyrimidine synthesis and the translational machinery were down-regulated in the bacteria cells under glucose starvation [14].

The species of the *Paracoccidioides* genus represent the causative agents of paracoccidioidomycosis (PCM), one of the most frequent systemic mycoses in Latin America [17]. The genus comprises four phylogenetic lineages (S1, PS2, PS3, and *Pb*01-like). The phylogenetic analysis of many *Paracoccidioides* isolates has resulted in the differentiation of the genus into two species: *P. brasiliensis*, which represents a complex of three phylogenetic groups, and *P. lutzii*, which represents the *Pb*01-like isolates [18]–[21]. *Paracoccidioides* spp. grows as a yeast form in the host tissue and *in vitro* at 36°C, while it grows as mycelium under saprobiotic condition and in culture at room temperature (18–23°C). As the dimorphism is dependent on temperature, when the mycelia/conidia are inhaled into the host lungs, the transition of the mycelia to the pathogenic yeast phase occurs [22].

One of the first lines of defense faced by *Paracoccidioides* spp. during host invasion is the lung resident macrophages. Despite being phagocytosed, the fungus conidia differentiate into the parasitic yeast form that subverts the normally harsh intraphagosomal environment and survives and replicates into murine and human macrophages [23]. It has been proposed for PCM and other systemic mycoses that the fungal intracellular parasitism is a major event for disease establishment and progression in susceptible hosts. The survival inside the macrophage may allow fungal latency and/or dissemination from the lungs to several organs such as observed in *C. neoformans* [24], [25]. In this sense, *Paracoccidioides* spp. has evolved defense mechanisms to survive under nutritionally poor environments. It has been suggested that alternative carbon metabolism plays a role in the survival and virulence of *Paracoccidioides* spp. within the host [26], [27], as occurs in *C. albicans* and *C. neoformans* [9], [12]. Transcriptional analysis of *Paracoccidioides* spp. upon internalization by macrophages, as determined by a DNA microarray, consisting of 1,152 cDNA clones, showed that the fungus responds to the glucose-depleted environment found in the macrophage phagosome, by the expression of 119 classified genes, differentially transcribed. Genes involved in methionine biosynthesis (cystathionine β-lyase), oxidative stress response (superoxide dismutase and heat shock protein 60), and cytochrome electron transport system (cytochrome oxidase c) were induced by the fungus. Moreover, *Paracoccidioides* spp. reduced the expression of genes that are involved in the glycolysis pathway such as the key regulatory phosphofructokinase (*pfkA*) and genes related to cell wall polysaccharides such as β-glucan synthase (*fks*) [27]. In addition, studies of the transcriptome profiling from yeast cells of *Paracoccidioides* spp. derived from mouse liver revealed that the fungus most likely uses multiple carbon sources during liver infection. Genes encoding enzymes, regulators, and transporters in carbohydrate and lipid metabolism were significantly overexpressed. Ethanol production was also detected, indicating that it may be particularly important during infection [28].

Here, we described the response of *Paracoccidioides* facing carbon starvation using a high-throughput RNA Illumina sequencing (RNAseq) and quantitative proteome NanoUPLC-MS^E^, a two-dimensional liquid chromatography-tandem mass spectrometry approach. RNAseq is a developed approach to transcriptome profiling that uses deep-sequencing technologies and has already been applied to organisms such as *Saccharomyces cerevisiae*, *Arabidopsis thaliana*, mouse, and human cells [29]–[33]. With regard to proteomic analysis, our group has developed detailed proteome maps of the process of the fungus dimorphism, the response to iron and zinc deprivation, the fungus exoproteome, and the response to oxidative stress as well as comparative proteome maps of members of *Paracoccidioides* phylogenetic species [34]–[39]. In this study, a comprehensive response of *Paracoccidioides*, isolate *Pb*01, under carbon starvation was performed by transcriptional and proteomic approaches. To the best of our knowledge, this is the first description of high-resolution transcriptomics and proteomics applied to study the response of *Paracoccidioides* spp. to carbon starvation. We believe that the obtained data can be relevant in the understanding of the fungal establishment in the host.

## Materials and Methods

### *Paracoccidioides* maintenance and carbon starvation

*Paracoccidioides*, *Pb*01 (ATCC MYA-826), was used in the experiments. The yeast phase was cultivated for 7 days, at 36°C in BHI semisolid medium added to 4% (w/v) glucose. When required, the cells were grown for 72 h at 36°C in liquid BHI, washed with PBS 1×, and incubated at 36°C in a McVeigh/Morton (MMcM) medium with the following composition per 100 mL: KH_2_PO_4_ 0.15 g; MgSO_4_.7H_2_0 0.05 g; CaCl_2_.2H_2_0 0.015 g; (NH_4_)_2_SO_4_ 0.2 g; vitamin 1 mL and trace element supplements 0.1 mL). The stock vitamin solution contained, also per 100 mL: thiamine hydrochloride, 6.0 mg; niacin, 6.0 mg; calcium pantothenate, 6.0 mg; inositol, 1.0 mg; biotin, 0.1 mg; riboflavin, 1.0 mg; folic acid, 10 mg; choline chloride, 10 mg; and pyridoxine hydrochloride, 10 mg. The trace element solution contained, per 100 mL: H_3_BO_3_, 5.7 mg; CuSO_4_.5H_2_0, 15.7 mg; Fe(NH_4_)_2_(SO_4_)_2_.6H_2_O, 140.4 mg; MnSO_4_.14H_2_O, 8.1 mg; (NH_4_)6Mo_7_O_24_.4H_2_O, 3.6 mg; ZnSO_4_.7H_2_O, 79.2 mg, as described previously [40], except for removal of the amino acids. All components except the vitamin supplement were mixed, and the pH was adjusted to 7.0 with 1N NaOH. The vitamin solution was filter sterilized and added after the remainder of the medium had been autoclaved at 121°C for 15 min and cooled.

*Paracoccidioides* yeast cells were subjected to carbon starvation as following. The *Pb*01 yeast cells were grown for 72 h at 36°C in liquid BHI added to 4% (w/v) glucose. The cells were harvest and washed three times with PBS 1×. A total of 10^6^ cells/mL were inoculated in modified MMcM medium [40] with 4% (glucose, carbon source) or 0% of glucose (carbon starvation). The cells were incubated at 36°C.

### Quantitative real time PCR (qRT-PCR) analysis

Following *Paracoccidioides* incubation in carbon starving condition, cells were centrifuged at 1,500× *g*, frozen in liquid nitrogen, and disrupted by maceration as described in [38]. Briefly, cells were treated with TRIzol reagent (Invitrogen, Carlsbad, CA, USA). The manufacturer's protocol was followed to extract total RNA. The RNA was reversibly transcribed using the high capacity RNA-to-cDNA kit (Applied Biosystems, Foster City, CA, USA). We confirmed the specificity of each primer pairs for the target cDNA by the visualization of a single PCR product following agarose gel electrophoresis and melting curve analysis. The cDNA was quantified by qRT-PCR using a SYBR green PCR master mix (Applied Biosystems Step One Plus PCR System). qRT-PCR analysis was performed in biological triplicate for each cDNA sample as previously described [38]. The data were normalized using the constitutive gene encoding the *60S ribosomal L34* as the endogenous control. In order to analyze the reliability of the normalizer used in our qRT-PCRs we used data obtained from three different housekeeping genes and the software NormFinder (Aarhus University, Aarhus, Denmark). The software identify the most suitable reference genes as previously described in [41]. We used the *actin* (PAAG_00564), *tubulin alpha-1 chain* (PAAG_01647) and *60S ribosomal protein L34* (PAAG_00746) genes and the results show that the *L34* is the best gene to be used as normalizer in our qRT-PCRs. It was demonstrated by the lower stability value by comparing with *actin* and *tubulin* genes after two different runs (Table S1). The *60S ribosomal L34* gene was amplified in each set of qRT-PCR experiments and was presented as relative expression in comparison to the experimental control cells value set at 1. Data were expressed as the mean ± standard deviation of the biological triplicates of independent experiments. Standard curves were generated by diluting the cDNA solution 1∶5. Relative expression levels of genes of interest were calculated using the standard curve method for relative quantification [42]. Statistical comparisons were performed using the student's t test and p-values≤0.05 were considered statistically significant. The specific primers, both sense and antisense, are described in Table S2.

### Western blot analysis

Proteins were fractionated by 12% SDS-polyacrylamide gel electrophoresis, and stained with Coomassie Blue R or transferred to Hybond ECL membrane (GE Healthcare). Membranes were blocked for 1 h at room temperature in a solution containing 10% (w/v) skim milk powder and 0.1% Tween 20 in Tris-buffered saline (TBS-T). The primary polyclonal antibody anti-isocitrate lyase [43] was diluted in blocking solution and incubated with the membrane for 1 h at room temperature. Membranes were washed in Tris-buffered saline and then incubated with alkaline phosphatase conjugated secondary antibodies for another hour at room temperature. Labeled bands were revealed with 5-bromo-4-chloro-3-indolylphosphate/nitroblue tetrazolium and negative controls were obtained with rabbit preimmune. Images from western blots were acquired with ImageMaster 2D Platinum 6.0 (Geneva Bioinformatics, GeneBio). Raw Tiff images were analyzed by densitometric analysis of immunoblotting bands using the software AphaEaseFC (Genetic technologies Inc.). Pixel intensity for the analyzed bands was generated and expressed as Integrated Density Values (IDV).

### High-throughput mRNA sequencing (RNA-seq)

Following *Paracoccidioides* growth in the presence or not of carbon for 6 h, cells were treated with TRIzol reagent (Invitrogen, Carlsbad, CA, USA) to obtain RNA molecules, from biological replicates. The cDNAs libraries were prepared from poly(A)-fragment selected mRNA and processed on the Illumina HiSeq2000 Sequencing System (http://www.illumina.com/). As a result, approximately 40 million of reads of 100 bp paired-end sequencing were obtained for each sample. The sequencing reads were mapped to reference the *Paracoccidioides* genome (*Pb*01), (http://www.broadinstitute.org/annotation/genome/paracoccidioides_brasiliensis/MultiHome.html), using the Bowtie 2 tool [44]. Mapped reads data were analyzed by the DEGseq package [45]. Briefly, each read was allowed to alignment in just one site of the genome and the reads were counted. The default parameters were used to perform the alignment. The number of mismatches allowed in seed alignment (-N) is 0, and the length of each seed (-L) is 20. The fold change selection method was used for differentially expressed genes selection using a Fisher exact test, and a p-value of 0.001 was considered to select the genes. From the selected genes, the 2-fold change cut-off was considered. Genes with log2 (fold change) higher than 1 or less than −1 were selected and classified as up- and down-regulated genes, respectively. Gene's identifications and annotations were determined from the *Paracoccidioides* genome database (http://www.broadinstitute.org/annotation/genome/paracoccidioides_brasiliensis/MultiHome.html). The biological processes were obtained using the Pedant on MIPS (http://pedant.helmholtz-muenchen.de/pedant3htmlview/pedant3view?Method=analysis&Db=p3_r48325_Par_brasi_Pb01) which provides a tool to browse and search the Functional Categories (FunCat) of proteins. All scripts can be obtained on request.

### Sample preparation and NanoUPLC-MS^E^ data acquisition

Following *Paracoccidioides* cell incubation in carbon-starved media for up to 48 h, the cells were centrifuged at 1,500× *g*, resuspended in a 50 mM ammonium bicarbonate pH 8.5 solution and disrupted using glass beads and bead beater apparatus (BioSpec, Oklahoma, USA) in 5 cycles of 30 sec, while on ice. The cell lysate was centrifuged at 10,000× *g* for 15 min at 4°C and the supernatant was quantified using the Bradford reagent (Sigma-Aldrich) [46]. The samples were analyzed using nanoscale liquid chromatography coupled with tandem mass spectrometry. Sample aliquots (50 µg) were prepared for NanoUPLC-MS^E^ as previously described [47], [48]. Briefly, 50 mM ammonium bicarbonate was added and was followed by addition of 25 µL of RapiGEST (0.2% v/v) (Waters Corp, Milford, MA). The solution was vortexed and then incubated at 80°C for 15 min; 2.5 µL of a 100 mM DTT solution was then added and incubated for 30 min at 60°C. The sample was cooled at room temperature and 2.5 µL of 300 mM iodoacetamide was added followed by sample incubation in a dark room for 30 min. A 10 µL aliquot of trypsin (Promega, Madison, WI, USA) prepared with 50 mM ammonium bicarbonate to 50 ng/uL, was added. The sample was vortexed slightly and digested at 37°C overnight. Following the digestion, 10 µL of 5% (v/v) trifluoroacetic acid was added to hydrolyze the RapiGEST, followed by incubation at 37°C for 90 min. The sample was centrifuged at 18,000× *g* at 6°C for 30 min, and the supernatant was transferred to a Waters Total Recovery vial (Waters Corp). A solution of one pmol.ul^−1^ MassPREP Digestion Standard [rabbit phosphorilase B (PHB)] (Waters Corp) was used to prepare the final concentration of 150 fmol.ul^−1^ of the PHB. The buffer solution of 20 mM ammonium formate (AF) was used to increase the pH. The digested peptides were separated further via NanoUPLC-MS^E^ and analyzed using a nanoACQUITY system (Waters Corporation, Manchester, UK).

### Data processing and protein identification

Mass spectrometry data obtained from NanoUPLC-MS^E^ were processed and searched using ProteinLynx Global Server (PLGS) version 3.0 (Waters Corp) as previously described [49]. Protein identifications and quantitative data packaging were performed using dedicated algorithms [50], [51] and a search against the *Paracoccidioides* database (http://www.broadinstitute.org/annotation/genome/paracoccidioides_brasiliensis/MultiHome.html). The ion detection, clustering, and log-scale parametric normalizations were performed in PLGS with an Expression^E^ license installed (Waters, Manchester, UK). The intensity measurements were typically adjusted for these components, i.e., the deisotoped and charge state reduced EMRTs that were replicated throughout the entire experiment for the analysis at the EMRT cluster level. STY phosphorylations were set as variable modification. Components were typically clustered with a 10 ppm mass precision and a 0.25 min time tolerance against the database-generated theoretical peptide ion masses with a minimum of one matched peptide. The alignment of elevated-energy ions with low-energy precursor peptide ions was performed with an approximate precision of 0.05 min. One missed cleavage site was allowed. The precursor and fragmention tolerances were determined automatically.

The protein identification criteria also included the detection of at least three fragment ions per peptide, 7 fragments per protein and the determination of at least one peptide per protein. The maximum protein mass was set to 600 kDa and trypsin was chosen as the primary digest reagent. The identification of the protein was allowed with a maximum 4% false positive discovery rate in at least two out of three technical replicate injections. Using protein identification replication as a filter, the false positive rate was minimized because false positive protein identifications, i.e., chemical noise, have a random nature and do not tend to replicate across injections. For the analysis of the protein identification and quantification level, the observed intensity measurements were normalized to the intensity measurement of the identified peptides of the digested internal standard. Protein and peptides tables generated by PLGS were merged and the dynamic range of the experiments, peptides detection type, and mass accuracy were determined for each condition as described in [47] by setting the minimum repeat rate for each protein in all replicates to 2. Normalization was performed with a protein that showed no significant difference in abundance in all injections [52] to accurately compare the expression protein level to carbon and carbon-starved samples.

### *Paracoccidioides* cell dry weight assay

*Paracoccidioides* yeast cells were grown for 72 h at 36°C in liquid BHI, washed with PBS 1×, and filtered using a nylon mesh filter to yield small and non-aggregated cells. A total of 5×10^7^ cells/50 mL were inoculated in modified MMcM medium [40] with carbon source (4% glucose) or under carbon starvation (absence of glucose) and were incubated at 36°C. In each time-point, 10 mL of culture were centrifuged at 1,500× *g* and the supernatants were carefully removed. The cells were ressuspended in PBS 1× up to 500 µl and subjected to 95°C heating for 1 h. The cells were centrifuged, frozen in liquid nitrogen and lyophilized for 24 h. Dry weight was determined. Data are expressed as the mean ± standard deviation of the triplicates of independent experiments. Statistical comparisons were performed using the Student's t test and p-values≤0.05 were considered statistically significant.

### *Paracoccidioides* cell viability analysis

The viability was determined by membrane integrity analysis using propidium iodide as dead cells marker as previously described [34], [35]. Briefly, cell suspension (10^6^ yeast cells/mL) were centrifuged and the supernatant was discarded. The cells were stained by addition of the propidium iodide solution (1 µg/mL) for 20 min in the dark at room temperature. After dye incubation, stained cell suspension was immediately analyzed in a C6 Accuri flow cytometer (Accuri Cytometers, Ann Arbor, MI, USA). A minimal of 10,000 events per sample was acquired with the FL3-H channel. Data was collected and analyzed using FCS Express 4 Plus Research Edition software (Denovo Software, Los Angeles, CA, USA).

### Ethanol measurement assay

The concentration of ethanol was quantified by enzymatic detection kit according to the manufacturer's instruction (UV-test for ethanol, RBiopharm, Darmstadt, Germany). Ethanol is oxidized to acetaldehyde by the enzyme alcohol dehydrogenase, in the presence of nicotinamide-adenine dinucleotide (NAD). Acetaldehyde is quantitatively oxidized to acetic acid in the presence of aldehyde dehydrogenase, releasing NADH, which is determined by means of its absorbance at 340 nm. *Paracoccidioides Pb*01 yeast cells were subjected or not to carbon starvation, and 10^6^ cells were used to assay. Briefly, cells were counted, centrifuged, and lysed using glass beads and bead beater apparatus (BioSpec, Oklahoma, USA) in 5 cycles of 30 sec, keeping the samples on ice. The cell lysate was centrifuged at 10,000× *g* for 15 min at 4°C and the supernatant was used for enzymatic assay according to the manufacturer's instructions. The concentrations of ethanol were obtained in triplicate.

### Enzyme activity assays

Following *Paracoccidioides* growth under carbon source (4% glucose) or carbon starvation (absence of glucose) the cells were centrifuged at 1,500× *g*, resuspended in a solution containing 20 mM Tris-HCl, pH 8.8, 2 mM CaCl_2_ [53] and disrupted using glass beads and bead beater apparatus (BioSpec, Oklahoma, USA) in 5 cycles of 30 sec, while on ice. The cell lysate was centrifuged at 10,000× *g* for 15 min at 4°C and the supernatant was quantified using the Bradford reagent (Sigma-Aldrich) [46]. Formamidase activity was determined by measuring the amount of ammonia formation as previously described [38], [54]. One µg of *Paracoccidioides* total protein extract prepared as described above was added to 200 µl of a 100 mM formamide substrate solution in 100 mM phosphate buffer containing 10 mM of EDTA, pH 7.4. Samples were incubated at 37°C for 30 min. A total of 400 µl of phenol-nitroprusside and the same volume of alkaline hypochlorite (Sigma Aldrich, Co.) were added on the tube. The samples were then incubated for 6 min at 50°C and the absorbance was read at 625 nm. The amount of ammonia released for each sample was determined by comparing to a standard curve. One unit (U) of formamidase specific activity was defined as the amount of enzyme required to hydrolyze 1 µmol of formamide (corresponding to the formation of 1 µmol of ammonia) per min per mg of total protein.

Isocitrate lyase activity was determined by measuring the formation of glyoxylate as its phenylhydrazone derivative [55]. Glyoxylate-phenylhydrazone formation was determined by measuring the absorbance at 324 nm, using an extinction coeficient of 16.8 mM^−1^ cm^−1^, in a reaction mixture containing 2 mM threo-D,L-isocitrate (Sigma Aldrich, Co.), 2 mM MgCl_2_, 10 mM phenylhydrazine HCl (Sigma Aldrich, Co.), 2 mM dithiothreitol and 50 mM potassium phosphate at pH 7.0. Specific activity was determined as the amount of enzyme required to form 1 µmol of glyoxylate-phenylhydrazone per min per mg of total protein. For both assays, the statistical comparisons were performed using the Student's t test and p-values≤0.05 were considered statistically significant.

### Macrophage infection assays

To evaluate whether carbon starvation influenced the internalization and survival of *Paracoccidioides*, the survival rate (viable fungi after co-cultivation) was determined by quantifying the number of colony-forming units (CFUs) recovered from macrophage infection. Macrophages, cell line J774 A.1 (Rio de Janeiro Cell Bank – BCRJ/UFRJ, accession number: 0121), maintained in RPMI medium (RPMI 1640, Vitrocell, Brazil), with 10% FBS (fetal bovine serum, [v/v]) were used in assays. A total of 10^6^ macrophages were seeded into each well of a 24-well tissue culture plate and 100 U. mL^−1^ of IFN-gamma (murine IFN-γ, PeproTech, Rocky Hill, New Jersey, USA) was used for 24 h at 36°C with 5% CO_2_ for activation of macrophages [56]. Prior to co-cultivation, *Paracoccidioides* yeast cells were grown in BHI liquid medium (4% [w/v] glucose, 3.7% [w/v] brain heart infusion, pH 7.2) for 72 h and subjected to both conditions, a carbon source (4% [w/v] of glucose) or carbon starvation (no glucose) at 36°C, in McVeigh/Morton medium (MMcM) for 48 h. The same ratio of 1∶2.5 macrophage∶yeast was used for infection of both, carbon and carbon-starved yeast cells. The cells were co-cultivated for 24 h with 5% CO_2_ at 36°C to allow fungal internalization. Prior to macrophages lysis, dilutions of the supernatant (culture from co-cultivation) removed by aspiration, were plated in BHI medium supplemented with 5% FBS (fetal bovine serum, [v/v]) incubated in 5% CO_2_ at 36°C for 10 days. The number of viable cells was determined based on the number of CFUs. Infected macrophages were lysed with distilled water and dilutions of the lysates were plated in BHI medium supplemented with 5% FBS (fetal bovine serum, [v/v]). Colony-forming units (CFU) were determined after growth at 36°C, in 5% CO_2_, for 10 days. For both experiments, the CFUs data were expressed as the mean value ± the standard deviation from triplicates and the statistical analyses were performed using Student's t test.

To analyze the expression of genes from *Paracoccidioides* yeast cells infecting macrophages, the same cell line described above was used. *Paracoccidioides* yeast cells were cultivated in BHI liquid medium (4% [w/v] glucose, 3.7% [w/v] brain hearth infusion, pH 7.2) for 72 h and incubated with macrophages for 24 h, followed by washing with distilled water to promote macrophages lysis. RNAs and cDNAs were obtained as previously described. Specific oligonucleotides were used to amplify the *fructose-1,6-biphosphatase*, *isocitrate lyase* and *3-ketoacyl-CoA thiolase* genes from *Pb*01 yeast cells. The negative amplification was obtained when cDNAs were used only from macrophages.

To determine the number of adhered/internalized fungi cells by macrophages, the same macrophage cell line described above was used. A total of 10^6^ macrophages were plated on glass coverslips per 12-well tissue culture plate and 100 U. mL^−1^ of IFN-gamma (murine IFN-γ, PeproTech, Rocky Hill, New Jersey, USA) was used for 24 h at 36°C with 5% CO_2_ for its activation, as described above [56]. Prior to co-cultivation, *Paracoccidioides* yeast cells were grown in BHI liquid medium (4% [w/v] glucose, 3.7% [w/v] brain heart infusion, pH 7.2) for 72 h, and subsequently transferred to McVeigh/Morton medium (MMcM), containing or not a carbon source, for 48 h, at 36°C. The same ratio of 1∶2.5 macrophage∶yeast was used for infection in both conditions. The cells were co-cultivated for 6 and 24 h with 5% CO_2_ at 36°C, to allow fungal adhesion/internalization. The supernatants were then aspirated, the monolayer was gently washed twice with PBS 1× to remove any non-adhered/internalized yeast cells, and the samples were processed for light microscopy. The glass coverslips were fixed with methanol and stained with Giemsa (Sigma). The cells were observed using the Axio Scope A1 microscope and digital images were acquired using the software AxioVision (Carl Zeiss AG, Germany). A total of 300 macrophages were counted to determine the average number of adhered/internalized fungal cells, as described before [37], [57]. For both experiments, the number of adhered/internalized fungal cells was shown in percentage of the total as the mean value ± the standard deviation from triplicates. The statistical analyses were performed using Student's t test.

## Results

### Effect of carbon starvation on transcripts and proteins of *Paracoccidioides*

**Figure 1:**
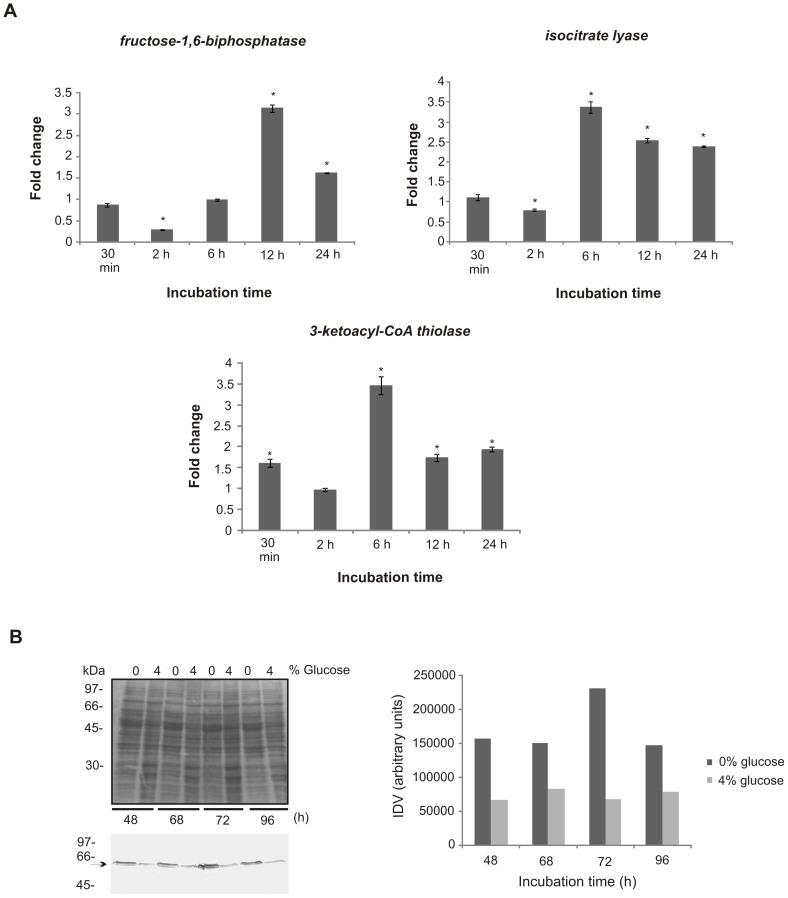
Effect of carbon starvation in transcripts and protein expression in *Paracoccidioides* yeast cells. (**A**) The expression of *fructose-1,6-biphosphatase, isocitrate lyase* and *3-ketoacyl-CoA thiolase* genes in *Pb*01 yeast cells grown in MMcM medium with 4% (carbon source) or absence of glucose (carbon starvation) were analyzed. The cells were incubated at 36°C for several time intervals. The data were normalized using the constitutive gene encoding the 60S ribosomal L34 gene as the endogenous control and are presented as relative expression in comparison to the experimental control cells value set at 1. Data are expressed as the mean ± standard deviation of the triplicates of independent experiments. *, significantly different from the control, at a p value of ≤0.05. (**B**) Proteins (50 µg) of *Pb*01 yeast cells were incubated at 36°C in MMcM medium with 4% or 0% of glucose and the abundance of *Pb*Icl was analyzed by western blotting. The proteins were fractionated by one-dimensional gel electrophoresis. The proteins were blotted onto a nitrocellulose membrane and the ∼60 kDa protein species was detected by using the rabbit polyclonal antibody anti-*Pb*Icl [43]. Densitometric analysis of immunoblotting bands was performed using the software AphaEaseFC.

We first sought to set up a time-point to analyze the fungus response to carbon starvation at transcriptional and proteomic levels. Hence, we analyzed gene and protein expression of genes known to be regulated under carbon-limited microenvironments in other fungi [9], [12], by using qRT-PCR and western blot assays, in *Pb*01 (Fig. 1A). Changes in the expression of genes encoding fructose-1,6-biphosphatase, isocitrate lyase, and 3-ketoacyl-CoA thiolase, which are representatives of gluconeogenesis, the glyoxylate cycle, and β-oxidation, respectively, were analyzed in the *Paracoccidioides*, *Pb*01, yeast cells subjected to carbon starvation. As depicted in Fig. 1A, carbon starvation promoted the increase of gene expression at 6 h and at 12 h for the treatments. At protein level, the differential expression of isocitrate lyase suggested that *Paracoccidioides*, *Pb*01, up-regulated the glyoxylate cycle after 48 h of carbon starvation (Figs. 1B and S1). In this way, the 6 h and 48 h treatments were considered in further transcriptional and proteomic analysis, using a high-throughput RNA Illumina sequencing (RNAseq) and NanoUPLC-MS^E^, respectively.

### Transcriptomic analysis

The transcriptome analysis was performed using next generation sequencing and the *Paracoccidioides*, isolate *Pb*01, genome database (http://www.broadinstitute.org/annotation/genome/paracoccidioides_brasiliensis/MultiHome.html) was used as a reference genome for mapping the reads which were analyzed by DEGseq package [45]. For the global analysis, plotting graphs were performed (Fig. S2).The number of the reads counted for each transcript in carbon and carbon-starved conditions was represented by scattered dots (Fig. S2).The transcripts are represented by dots, which could present a different number of reads in each condition (Fig. S2A). We applied a statistical test to identify differentially expressed transcripts, represented by red dots (Fig. S2B). A significant number of genes were regulated during carbon starvation.

Although 1.5-fold change can be statistically significant [58], a cut-off of 2-fold change was applied to determine the up- and down-regulated transcripts (Tables S3 and S4, respectively) totaling 1,063 differentially expressed transcripts in *Pb*01 yeast cells under carbon starvation. A biological process classification was performed to gain a general understanding of the functional categories affected by carbon starvation. A total of 64.6% (687 transcripts) were represented by miscellaneous and unclassified categories, and the other 35.4% (376 transcripts) were represented by classified biological categories. The functional classifications and the percentage of up- and down-regulated transcripts in each classified category are shown in Fig. S3.

**Figure 2:**
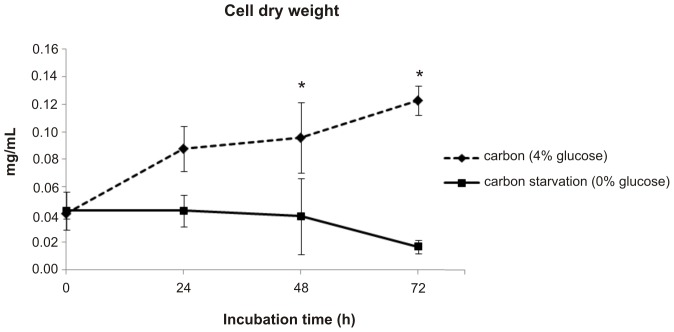
Growth of *Paracoccidioides* under carbon starvation. A total of 5.10^7^ cells/50 mL of *Paracoccidioides* yeast cells were incubated in minimal medium (MVM) with carbon (4% glucose) or not (0% glucose) up to 72 h. At time points 0, 24, 48 and 72 h, cells were collected, killed by heat, and lyophilized to determine the cell dry weight. Data are expressed as the mean ± standard deviation of the triplicates of independent experiments.*, significantly different from the carbon condition, at a p-value of ≤0.05.

The transcriptome analysis showed that transcripts associated with metabolism were the most represented during 6 h of carbon starvation in *Pb*01 (Fig. S3A). From these, approximately 27% were represented by up-regulated and 17% by down-regulated transcripts (Fig. S3B). Subcategories of metabolism related to amino acid, nitrogen/sulfur, C-compound/carbohydrate, lipid/fatty acid, purines, secondary, and phosphate metabolisms were regulated under carbon starvation stress, and all of them showed a higher number of up- than down-regulated transcripts (Tables S3 and S4). Other categories were also regulated in *Pb*01 under carbon starvation. The categories associated with protein fate, cell cycle/DNA processing, transcription, and cellular transport were largely represented in the transcriptome (Fig. S3A). The number of transcripts with increased or reduced expression was also investigated for these categories and the results show that, in contrast to metabolism, the number of down-regulated transcripts was generally higher than that of up-regulated transcripts for each category (Fig. S3B). Down-regulated transcripts associated with cell cycle, transcription, cell growth morphogenesis, and signal transduction could reflect the reduced growth of this fungus subjected to carbon starvation, as demonstrated in Fig. 2. Although the reduced growth of *Paracoccidioides* during carbon starvation, the cells viability, assayed using propidium iodide, was not significantly different from those cultivated with a carbon source (Fig. S4). In the same way, the cells are metabolically active as demonstrated by the high activity of the enzyme formamidase in *Paracoccidioides* grown under glucose deprivation (Fig. S5).

The cellular transport process was also representative in our transcriptome analysis. The abundance of specific transporters was elevated such as those of copper, hexoses, and monosaccharides (Table S3) indicating that carbohydrate, amino acid and metal-uptake processes are required for *Pb*01 cells to survival under carbon starvation. Additionally, the abundance of transcripts related to cellular response against ROS (reactive oxygen species) such as superoxide dismutases, catalase and cytochrome c peroxidase were also elevated (Table S3) indicating that *Paracoccidioides* possibly has evolved the ability to respond to oxidative stress also under carbon starvation.

### Proteomic analysis

The proteomic approach was performed using NanoUPLC-MS^E^ as previously described [47], [48]. This method has been shown to improve protein and proteome coverage compared to the conventional LC-MS/MS approach [48]. The resulting NanoUPLC-MS^E^ protein and peptide data generated by PLGS process are shown in Fig. S6, S7 and S8. First, the false positive rates of proteins from carbon and carbon starvation data were 0.34 and 0.27%, respectively. The experiments resulted in 3,327 and 3,842 identified peptides, where 45 and 57% of these were obtained from peptide match type data in the first pass, and 19 and 14% from the second pass [50] to carbon and carbon-starving conditions, respectively (Fig. S6). A total of 17 and 14% of total peptides were identified by a missed trypsin cleavage in carbon and carbon-starving conditions, respectively, whereas an in-source fragmentation rate of the same 4% was obtained for both (Fig. S6). Fig. S7 shows the peptide parts per million error (ppm) indicating that the majority, 94.8 and 95.7%, from identified peptides were detected with an error of less than 15 ppm for carbon and carbon starvation conditions, respectively. Fig. S8 depicts the results obtained from dynamic range detection indicating that a 3-log range concentration and a good detection distribution of high and low molecular weights were obtained for the both conditions.

A total of 421 differentially expressed proteins were identified in our proteomic analysis. As previously described [59], a 1.5-fold change was used as a threshold to determine the up- and down-regulated proteins (Tables S5 and S6, respectively). Approximately 20% of them (86 proteins) were represented by miscellaneous and unclassified categories, and the remaining 80% (335 proteins) were represented by classified biological categories. The biological processes and the percentage of up- and down- regulated proteins in each classified category are shown in Fig. S9.

The proteome analysis showed that proteins associated with metabolism were also the most represented during 48 h of carbon starvation in *Pb*01 (Fig. S9A). The metabolism was represented by amino acid, nitrogen/sulfur, C-compound/carbohydrate, lipid/fatty acid, purines, secondary, and phosphate metabolisms. All of these subcategories showed more up- than down-regulated proteins (Tables S5 and S6). Interestingly, the nitrogen/sulfur metabolism was detected as up-regulated only at protein level (Table S5). Other categories presented a high number of regulated proteins such as translation, protein fate, energy and cell defense. On the other hand, processes involved with transcription, cellular transport, cell growth/morphogenesis, and signal transduction presented a lower number of regulated proteins in which the majority was down-regulated (Fig. S9A and B).

Thus, a large part of the proteomic response to carbon starvation in *Pb*01 is involved in an increase of proteins associated with metabolism and reduction of those involved with core cellular processes, in agreement with transcriptome analysis.

### An overview of the *Paracoccidioides* responses to carbon starvation

**Figure 3:**
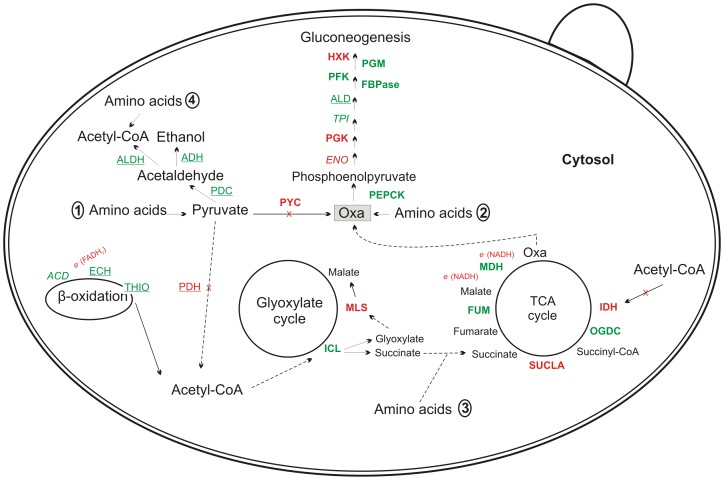
Overview of metabolic responses of *Paracoccidioides* to carbon starvation. The figure summarizes the data from transcriptome and proteomic analyses and suggests the mechanism and the first flow of carbon used by this fungus to overcome the carbon starvation stress. HXK: hexokinase; PGM: phosphoglucomutase; PFK: 6-phosphofructokinase; FBPase: fructose-1,6-biphosphatase; ALD: fructose-bisphosphate aldolase; TPI: triosephosphate isomerase; PGK: phosphoglycerate kinase; ENO: enolase; PEPCK: phosphoenolpyruvate carboxykinase; PYC: pyruvate carboxylase; PDC: pyruvate decarboxylase; ADH: alcohol dehydrogenase; ALDH: aldehyde dehydrogenase; ACD: acyl-CoA dehydrogenase; ECH: enoyl-CoA hydratase; THIO: 3-ketoacyl-CoA thiolase; PDH: pyruvate dehydrogenase; ICL: isocitrate lyase; MLS: malate synthase; IDH: isocitrate dehydrogenase; OGDC: 2-oxoglutarate dehydrogenase E1; SUCLA: succinyl-CoA ligase; FUM: fumarate hydratase (fumarase) and MDH: malate dehydrogenase. Enzymes were colored according to their differences in expression and labeled to indicate whether the data were obtained from transcriptome or proteomics. Italic, bold, and underlined labels indicate that the data were obtained from transcriptome, proteome, or both, respectively. Green or red indicate up- or down-regulated proteins, respectively. The numbers 1, 2, 3, and 4 indicate up-regulated amino acids involved in pyruvate, oxaloacetate, succinate, and acetyl-CoA production, respectively. 1) pyruvate production: *tryptophan* and **cysteine**. 2) oxaloacetate production: phenylalanine, **glutamate** and tyrosine. 3) succinate production: threonine. 4) acetyl-CoA production: threonine, *tryptophan*, tyrosine and **leucine**. Italic, bold, and underlined labels indicate that the amino acids accumulations were obtained from transcriptome, proteome, or both analyzes, respectively. OXA: oxaloacetate; *e^−^*: released electrons from enzymatic reaction.

The responses of the *Paracoccidioides, Pb*01, to carbon starvation, as revealed by transcriptome and proteomic analysis, are summarized in Fig. 3, which depicts the metabolic and energy adaptation of the fungus to this stress. Pathways associated with ethanol, acetyl-CoA, oxaloacetate, and consequently glucose production were induced. Moreover, amino acid degradation supply precursors such as pyruvate, oxaloacetate, succinate and also acetyl-CoA for glucose production pathways (Fig. 3).

**Figure 4:**
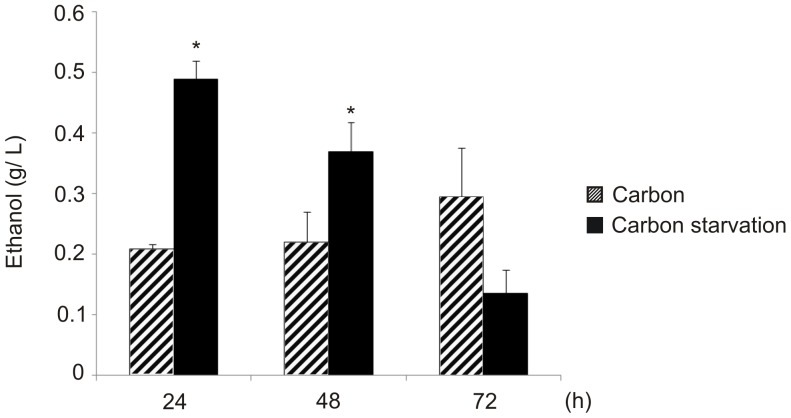
Ethanol detection in *Paracoccidioides*, *Pb*01 yeast cells, under carbon starvation. The concentration of ethanol (g/L) in *Paracoccidioides* yeast cells under carbon or carbon-starved conditions was determined. A total of 10^6^ cells were used for each sample, and the ethanol compound was quantified using the enzymatic detection kit (UV-test for ethanol, RBiopharm, Darmstadt, Germany). Data are expressed as the mean ± standard deviation of the biological triplicates of independent experiments. Student's *t*-test was used.*, significantly different from the carbon condition, at a p-value of ≤0.05.

**Figure 5:**
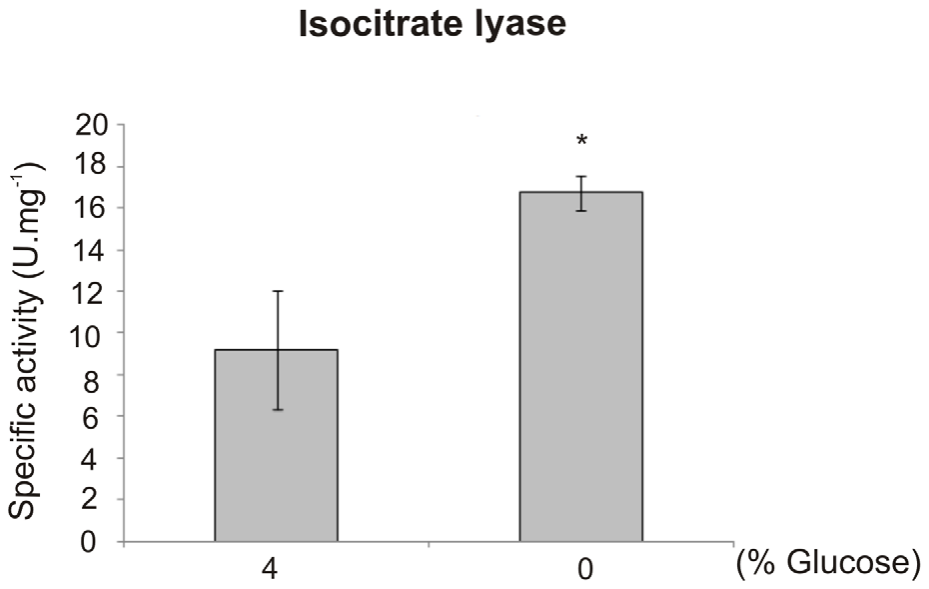
Isocitrate lyase activity assay. The activity was determined by measuring the formation of glyoxylate as its phenylhydrazone derivative in each condition. A total of 50 µg of each total protein extracts of *Paracoccidioides* under carbon and carbon-starvation (0% glucose) conditions for 48 h in MMcM medium was used. The specific activities were determined as the amount of enzyme required to form 1 µmol of glyoxylate-phenylhydrazone per minute per mg of total protein and are represented as U.mg^−1^. Errors bars represent standard deviation from three biological replicates while * represents p≤0.05.

Specific enzymes related to ethanol production were up-regulated in the absence of carbon sources. The ethanol molecule is derived from pyruvate that, in turn, is not involved directly in oxaloacetate production because the pyruvate carboxylase (PYC) enzyme is down-regulated (Fig. 3). Ethanol measurement was performed, and the results showed that after up to 48 h under carbon starvation, a significantly higher level of ethanol was produced compared with glucose-rich cells (Fig. 4). Regarding the acetyl-CoA molecule, several enzymes associated with its production from pyruvate, *via* the acetaldehyde precursor, and β-oxidation were also up-regulated. Once produced, acetyl-CoA may be used by the glyoxylate shunt to generate glyoxylate and succinate molecules. This is reinforced by fact that the TCA cycle enzyme isocitrate dehydrogenase is repressed, so the acetyl-CoA pool should be consumed by the glyoxylate cycle. Additionally, succinate can be converted in oxaloacetate by enzymes from the tricarboxylic acid cycle (Fig. 3). The activity of isocitrate lyase, a representative enzyme of the glyoxylate cycle, was also determined confirming our proteomic data and reinforcing our suggested carbon flow. The analysis revealed that a significant higher specific isocitrate lyase activity was obtained after *Paracoccidioides* yeast cells were subjected to carbon starvation for 48 h (Fig. 5).

The oxaloacetate molecule is a key intermediate of gluconeogenesis. Once produced, gluconeogenic enzymes convert it into glucose. We detected up-regulated specific enzymes that support this suggestion in *Pb*01 under carbon starvation, such as phosphoenolpyruvate carboxykinase (PEPCK), fructose-1,6-biphosphatase (FBPase), and phosphoglucomutase (PGM)(Fig. 3).

### The most up-regulated proteins in *Paracoccidioides* upon carbon starvation

In order to perform additional analysis, we applied a highly stringent criterion (≥5×-fold) to analyze the most induced or repressed proteins in yeast cells upon carbon starvation (Tables 1 and 2). Proteins which were detected only in carbon or carbon starved conditions were considered as down and up-regulated proteins at a high level (Table 1 and 2), respectively [52]. Even at this high cut-off, up-regulated proteins involved in amino acids degradation, in β-oxidation, in ethanol production, among others are present using this high stringent criterion (Table 1), in agreement with the metabolic overview presented in Fig. 3. In the same way, down regulated proteins such as pyruvate dehydrogenase and enzymes involved in fatty acids biosynthesis were detected using this highly stringent fold change criteria (Table 2). Proteins related to cell defense such as thioredoxin reductase, superoxide dismutase and cytochrome c oxidase were also detected among the up-regulated proteins.

**Table 1:** The most abundant up-regulated proteins of *Paracoccidioides* (*Pb*01) yeast cells under carbon starvation detected using NanoUPLC-MS^E^.

ID^a^	Annotation^b^	Fold change (log2)^c^	Biological process^d^
**METABOLISM**			
**Amino acid metabolism**			
PAAG_08166	4-hydroxyphenylpyruvate dioxygenase	#	glycine biosynthesis
PAAG_03138	alanine-glyoxylate aminotransferase	#	glycine biosynthesis
PAAG_01969	arginase	#	glutamate biosynthesis
PAAG_01144	aspartate aminotransferase	#	arginine biosynthesis
PAAG_01365	choline dehydrogenase	#	glycine biosynthesis
PAAG_08649	cysteine dioxygenase	#	cysteine degradation
PAAG_07954	gamma-glutamyl phosphate reductase	#	proline biosynthesis
PAAG_03506	glutamate decarboxylase	#	glutamate degradation
PAAG_07998	glutamate synthase small chain	#	glutamate biosynthesis
PAAG_07317	PENR2 protein	#	methionine biosynthesis
PAAG_08162	maleylacetoacetate isomerase	#	phenylalanine degradation
PAAG_04099	methylcrotonoyl-CoA carboxylase subunit alpha	#	leucine degradation
PAAG_02693	saccharopine dehydrogenase	#	lysine biosynthesis
PAAG_02901	S-adenosylmethionine synthetase	#	L-methionine biosynthesis
PAAG_06416	alanine racemase family protein	#	D- alanine biosynthethic process
**Nitrogen and sulfur metabolism**			
PAAG_07811	sulfite oxidase	#	nitrogen, sulfur and selenium metabolism
**C-compound and carbohydrate metabolism**			
PAAG_06953	short chain dehydrogenase/reductase family	2.96	carbohydrate metabolism_sugar epimerase family
PAAG_04181	sorbitol utilization protein SOU2	#	C-compound and carbohydrate metabolism
PAAG_02653	acetyl-coenzyme A synthetase	#	C-compound and carbohydrate metabolism
PAAG_00685	alpha-mannosidase	#	polysaccharide
PAAG_01935	formyl-coenzyme A transferase	#	oxalate catabolic process
PAAG_05984	glutaryl-CoA dehydrogenase	#	aromatic hydrocarbons catabolism
PAAG_03765	NADP-dependent glycerol dehydrogenase	#	Sugar, glucoside, polyol and carboxylate catabolism
PAAG_05416	NADP-dependent leukotriene B4 12-hydroxydehydrogenase	#	C-compound and carbohydrate metabolism
PAAG_06057	aldose 1-epimerase	#	hexose metabolic process
PAAG_02162	lactam utilization protein LamB	#	C-compound and carbohydrate metabolism
PAAG_01870	short-chain dehydrogenase/reductase SDR	#	Carbohydrate metabolism_sugar epimerase family
PAAG_03316	polysaccharide export protein	#	surface polysaccharide biosynthesis process
**Lipid, fatty acid and isoprenoid metabolism**			
PAAG_02163	acetyl-/propionyl-coenzyme A carboxylase alpha chain	3.03	lipid metabolism
PAAG_05249	aldehyde dehydrogenase	2.45	lipid and fatty acid metabolism_oxidation
PAAG_01928	peroxisomal dehydratase	#	Lipid, fatty acid and isoprenoid metabolism
PAAG_06099	glycerol-3-phosphate dehydrogenase	#	lipid metabolism
PAAG_01833	2-succinylbenzoate-CoA ligase	#	fatty acid metabolism
PAAG_06224	carnitine O-acetyltransferase	#	Lipid, fatty acid and isoprenoid metabolism
PAAG_07279	farnesyl pyrophosphate synthetase	#	lipid metabolism
PAAG_04142	NAD-dependent 15-hydroxyprostaglandin dehydrogenase	#	lipid metabolism
PAAG_04030	short-chain-fatty-acid-CoA ligase	#	lipid and fatty acid metabolism
**Purin nucleotide/nucleoside/nucleobase metabolism**			
PAAG_09072	mitochondrial nuclease	#	polynucleotide degradation
PAAG_04974	adenylosuccinate lyase	#	purine biosynthesis
PAAG_05803	inosine-5′-monophosphate dehydrogenase IMD2	#	purine nucleotide/nucleoside/nucleobase anabolism
PAAG_08856	nicotinate-nucleotide pyrophosphorylase	#	biosynthesis of the pyridine nucleotides NAD and NADP
PAAG_02633	ribose-phosphate pyrophosphokinase	#	nucleotide biosynthesis
PAAG_01751	cytidine deaminase	#	UMP synthesis
**Secundary metabolism**			
PAAG_00851	6,7-dimethyl-8-ribityllumazine synthase	#	biosynthesis of riboflavin
PAAG_01324	folic acid synthesis protein	#	folic acid-containing compound
PAAG_01934	riboflavin synthase alpha chain	#	biosynthesis of riboflavin
PAAG_04443	spermidine synthase	#	B complex vitamins
PAAG_02656	NADH-ubiquinone oxidoreductase 51 kDa subunit	#	ubiquinone
**ENERGY**			
**Glycolysis and gluconeogenesis**			
PAAG_01583	6-phosphofructokinase subunit beta	#	glycolysis
PAAG_06172	glucokinase	#	glycolysis
**Tricarboxylic-acid pathway**			
PAAG_00588	fumarate hydratase	#	TCA cycle
PAAG_04597	malate dehydrogenase	#	TCA cycle
**Electron transport and membrane-associated energy conservation**			
PAAG_02656	NADH-ubiquinone oxidoreductase 51 kDa subunit	#	electron transport
PAAG_04570	ATP synthase D chain, mitochondrial	#	respiration
PAAG_05605	ATP synthase delta chain	#	respiration
PAAG_08088	cytochrome b-c1 complex subunit 2	#	respiration
PAAG_06268	cytochrome c	#	electron transport
PAAG_00173	electron transfer flavoprotein subunit alpha	#	electron transport
PAAG_03599	formate dehydrogenase	#	electron transport
PAAG_01265	cytochrome b5	#	electron transport
PAAG_06796	cytochrome c oxidase polypeptide IV	#	electron transport
**Ethanol production**			
PAAG_02512	pyruvate decarboxylase	#	alcohol fermentation
PAAG_08248	alcohol dehydrogenase	#	alcohol fermentation
**Pentose Phosphate pathway**			
PAAG_05621	6-phosphogluconolactonase	#	pentose-phosphate shunt
PAAG_05146	ribose 5-phosphate isomerase A	#	pentose-phosphate shunt
**CELL CYCLE and DNA PROCESSING**			
PAAG_07608	DNA helicase	#	DNA repair
PAAG_08917	histone H2a	#	DNA processing
PAAG_07098	histone H4,1	#	DNA processing
PAAG_00126	histone H4,2	#	DNA processing
PAAG_08918	late histone H2B,L4	#	DNA processing
PAAG_05147	mitotic checkpoint protein BUB3	#	cell cycle control
PAAG_03054	G2/M phase checkpoint control protein Sum2	#	cell cycle control
**TRANSCRIPTION**			
PAAG_02467	Transcription initiation factor TFIID subunit 14	3.22	transcription regulation
PAAG_06250	nuclear cap-binding protein	#	mRNA processing
PAAG_05609	C2H2 type zinc finger domain-containing protein	#	transcriptional control
PAAG_02268	DNA-directed RNA polymerase I ssubunit RPA12	#	rRNA synthesis
PAAG_05397	DNA-directed RNA polymerase II subunit RPB1	#	rRNA synthesis
PAAG_02255	mRNA decapping hydrolase	#	mRNA processing
PAAG_02437	U2 small nuclear ribonucleoprotein B	#	splicing
**TRANSLATION**			
PAAG_06886	zinc finger protein GIS2	5.54	positive regulation of translation
PAAG_02865	translation initiation factor RLI1	3.92	translation
PAAG_05882	translation factor SUI1	#	translation
PAAG_01834	60S ribosomal protein L16	#	ribosome biogenesis
PAAG_04425	60S ribosomal protein L22	#	ribosome biogenesis
PAAG_05233	60S ribosomal protein L26	#	ribosome biogenesis
PAAG_01939	60S ribosomal protein L27-A	#	ribosome biogenesis
PAAG_08847	60S ribosomal protein L28	#	ribosome biogenesis
PAAG_06627	60S ribosomal protein L32	#	ribosome biogenesis
PAAG_00648	60S ribosomal protein L33-B	#	ribosome biogenesis
PAAG_06569	60S ribosomal protein L43	#	ribosome biogenesis
PAAG_03019	60S ribosomal protein L6-B	#	ribosome biogenesis
PAAG_04998	60S ribosomal protein L8-B	#	ribosome biogenesis
PAAG_08285	50S ribosomal protein L12	#	ribosome biogenesis
PAAG_01435	40S ribosomal protein S16	#	ribosome biogenesis
PAAG_03322	40S ribosomal protein S20	#	ribosome biogenesis
PAAG_00385	40S ribosomal protein S23	#	ribosome biogenesis
PAAG_03816	40S ribosomal protein S4	#	ribosome biogenesis
PAAG_01050	cytosolic large ribosomal subunit protein L30	#	ribosome biogenesis
PAAG_07028	histidyl-tRNA synthetase	#	translation
PAAG_08172	lysyl-tRNA synthetase	#	translation
PAAG_03951	prolyl-tRNA synthetase	#	translation
PAAG_08702	seryl-tRNA synthetase	#	translation
**PROTEIN FATE**			
PAAG_07802	proteasome component PRE6	2.8	protein degradation
PAAG_07500	xaa-Pro aminopeptidase	#	proteolysis
PAAG_07319	xaa-Pro aminopeptidase	#	proteolysis
PAAG_03719	Thimet oligopeptidase	#	proteolysis
PAAG_01472	ubiquitin-conjugating enzyme	#	protein degradation
PAAG_00852	proteasome component C1	#	proteasomal degradation (ubiquitin/proteasomal pathway)
PAAG_00868	proteasome component PRE4	#	proteasomal degradation (ubiquitin/proteasomal pathway)
PAAG_03536	proteasome component PRE5	#	proteasomal degradation (ubiquitin/proteasomal pathway)
PAAG_02720	proteasome-activating nucleotidase	#	proteasomal degradation (ubiquitin/proteasomal pathway)
PAAG_03279	aminopeptidase	#	proteolysis
PAAG_00664	aspartyl aminopeptidase	#	protein/peptide degradation
PAAG_03464	bleomycin hydrolase	#	proteolysis
PAAG_05583	cysteine protease PalB	#	proteolysis
PAAG_00768	peptidase family protein	#	proteolysis
PAAG_05417	mitochondrial-processing peptidase subunit beta	#	proteolysis
PAAG_00739	peptidyl-prolyl cis-trans isomerase B	#	protein folding
PAAG_02155	peroxisomal targeting signal 2 receptor (PTS2)	#	protein fate
PAAG_04555	sarcosine oxidase	#	protein modification
PAAG_00472	serine/threonine-protein phosphatase	#	protein dephosphorilation
PAAG_03573	vacuolar protein sorting-associated protein	#	protein fate
**BINDING**			
PAAG_07038	APAF1-interacting protein	#	metal binding
PAAG_08026	MYB DNA-binding domain-containing protein	#	DNA binding
PAAG_07753	RNA-binding La domain-containing protein	#	RNA binding
**TRANSPORT**			
PAAG_03577	ABC drug exporter AtrF	#	drug/toxin transport
PAAG_05425	Golgi membrane protein (Coy1)	#	Golgi vesicle transport
PAAG_04571	nascent polypeptide-associated complex subunit alpha	#	protein transport
PAAG_08082	plasma membrane ATPase	#	hydrogen ion transport
**SIGNAL TRANSDUCTION**			
PAAG_02973	diploid state maintenance protein chpA	#	cellular signalling
PAAG_04261	signal recognition particle 54 kDa protein	#	GTP catabolic process
PAAG_02377	rho GDP-dissociation inhibitor	#	regulator of G-protein signaling
**CELL RESCUE, DEFENSE and VIRULENCE**			
PAAG_07020	thioredoxin reductase	3.78	cell redox homeostase
PAAG_02926	superoxide dismutase	#	detoxification
PAAG_03502	cytochrome c peroxidase	#	oxidative stress response
PAAG_00566	aflatoxin B1 aldehyde reductase member 2	#	detoxification
PAAG_06606	cyanate hydratase	#	stress response/cyanate metabolic process
PAAG_06947	gamma-glutamyltranspeptidase	#	cell redox homeostase
PAAG_02548	hydroxyacylglutathione hydrolase	#	glutathione biosynthetic process/stress response
PAAG_08277	nitroreductase family protein	#	detoxification
**CELL GROWTH/MORPHOGENESIS**			
PAAG_00997	actin-interacting protein	#	actin depolymerization
PAAG_00875	Arp2/3 actin-organizing complex subunit Sop2	#	actin filament organization
PAAG_03624	Arp2/3 complex subunit Arc16	#	actin filament organization
PAAG_04602	mannosyl-oligosaccharide glucosidase	#	cell wall biogenesis
PAAG_02186	nuclear segregation protein Bfr1	#	meiosis
PAAG_01931	phosphoacetylglucosamine mutase	#	cell wall processing
**MISCELLANEOUS**			
PAAG_02139	methyltransferase family	#	methyltransferase
PAAG_01302	phosphorylase family protein	#	phosphoprotein
PAAG_03233	oxidoreductase	#	oxidation-reduction process
PAAG_00712	N-acetyltransferase	#	acetyltransferase
PAAG_06955	thiol methyltransferase	#	thiol methyltransferase activity

a Identification of differentially regulated proteins from *Paracoccidioides* genome database (http://www.broadinstitute.org/annotation/genome/paracoccidioides_brasiliensis/MultiHome.html) using the ProteinLynx Global Server (PLGS) version 3.0 (Waters Corporation. Manchester. UK);

b Proteins annotation from *Paracoccidioides* genome database or by homology from NCBI database (http://www.ncbi.nlm.nih.gov/);

c Protein expression profiles in **log2**-fold change (**5×-threshold**) obtained from ProteinLynx Global Server (PLGS) analysis normalized with internal standard.

d Biological process of differentially expressed proteins from MIPS (http://pedant.helmholtz-muenchen.de/pedant3htmlview/pedant3view?Method=analysis&Db=p3_r48325_Par_brasi_Pb01) and Uniprot database (http://www.uniprot.org/).

# : identified only in carbon starvation condition.

**Table 2:** The most abundant down-regulated proteins of *Paracoccidioides* (*Pb*01) yeast cells under carbon starvation detected using NanoUPLC-MS^E^.

ID^a^	Annotation^b^	Fold change (log2)^c^	Biological process^d^
**METABOLISM**			
**Amino acid metabolism**			
PAAG_03569	1,2-dihydroxy-3-keto-5-methylthiopentene dioxygenase	*	methionine biosynthesis
PAAG_05328	3-isopropylmalate dehydrogenase A	*	leucine biosynthesis
PAAG_07605	acetolactate synthase small subunit	*	L-isoleucine biosynthesis
PAAG_03043	adenylyl-sulfate kinase	*	methionine biosynthesis
PAAG_07563	asparagine synthetase	*	asparagine biosynthesis
PAAG_05198	chorismate mutase	*	cysteine biosynthesis and aromatic groups
PAAG_07813	cysteine synthase	*	cysteine biosynthesis
PAAG_07089	homocitrate synthase	*	lysine biosynthesis
PAAG_04348	homoserine kinase	*	threonine biosynthesis
PAAG_07102	pentafunctional AROM polypeptide	*	aromatic group biosynthesis
PAAG_05929	sulfate adenylyltransferase	*	cysteine and methionine biosynthesis
**C-compound and carbohydrate metabolism**			
PAAG_02975	2,4-dihydroxyhept-2-ene-1,7-dioic acid aldolase	*	phenylacetate catabolic process
PAAG_02769	pyruvate dehydrogenase protein X component	*	carbohydrate metabolism
PAAG_03774	S-(hydroxymethyl)glutathione dehydrogenase	*	C-compound and carbohydrate metabolism/metane metabolism
PAAG_00799	uroporphyrinogen decarboxylase	*	porphyrin biosynthesis
**Lipid, fatty acid and isoprenoid metabolism**			
PAAG_02211	GDSL Lipase/Acylhydrolase	*	lipid metabolism
PAAG_07013	enoyl-CoA hydratase/carnithine racemase	*	lipid, fatty acid and isoprenoid metabolism
PAAG_01525	fatty acid synthase subunit alpha reductase	*	lipid and fatty acid biosynthesis
PAAG_01524	fatty acid synthase subunit beta dehydratase	*	lipid and fatty acid biosynthesis
PAAG_00869	fumarylacetoacetate hydrolase domain-containing protein	*	lipid, fatty acid and isoprenoid metabolism
PAAG_06215	hydroxymethylglutaryl-CoA lyase	*	lipid, fatty acid and isoprenoid metabolism
**Purin nucleotide/nucleoside/nucleobase metabolism**			
PAAG_06906	adenine phosphoribosyltransferase	*	purin nucleotide/nucleoside/nucleobase metabolism
PAAG_07529	orotidine 5′-phosphate decarboxylase	*	pyrimidine biosynthesis
PAAG_01437	uricase	*	purine metabolism
**Secundary metabolism**			
PAAG_00047	siroheme synthase	*	cobalamin (vitamin B12) biosynthesis
PAAG_01519	inositol monophosphatase	*	secondary metabolism
**ENERGY**			
**Glycolysis and gluconeogenesis**			
PAAG_01015	hexokinase	*	glycolysis
**Electron transport and membrane-associated energy conservation**			
PAAG_07285	vacuolar ATP synthase catalytic subunit A	*	ATP hydrolysis coupled proton transport
PAAG_06288	vacuolar ATP synthase subunit B	*	ATP hydrolysis coupled proton transport
PAAG_06155	vacuolar ATP synthase subunit E	*	ATP hydrolysis coupled proton transport
**Pentose Phosphate pathway**			
PAAG_00633	glucose-6-phosphate 1-dehydrogenase	*	glucose metabolism
**CELL CYCLE and DNA PROCESSING**			
PAAG_03834	vacuolar sorting-associated protein	*	protein sorting in cell division
PAAG_07773	cyclin-dependent kinases regulatory subunit	*	mitotic cell cycle and cell cycle control
PAAG_00106	histone acetyltransferase type B catalytic subunit	*	DNA repair
PAAG_02055	histone chaperone asf1	*	DNA replication
PAAG_01943	spindle pole body component alp6	*	cell cycle
**TRANSCRIPTION**			
PAAG_01695	arsenite resistance protein Ars2	*	transcription
PAAG_04161	transcription factor	*	transcription control
PAAG_08234	transcription factor RfeF	*	transcription control
PAAG_01733	28 kDa ribonucleoprotein	*	transcriptional control
PAAG_00101	small nuclear ribonucleoprotein	*	splicing
PAAG_01630	small nuclear ribonucleoprotein LSM2	*	splicing
PAAG_02329	U2 small nuclear ribonucleoprotein A	*	splicing
PAAG_07983	ribonuclease H	*	RNA processing
PAAG_06966	RNA methyltransferase	*	RNA processing
**TRANSLATION**			
PAAG_00765	60S ribosomal protein L36	*	ribosome biogenesis
PAAG_00689	ATP-dependent RNA helicase eIF4A	*	protein biosynthesis
PAAG_07283	ATP-dependent RNA helicase FAL1	*	ribossome biogenesis
PAAG_06140	eukaryotic translation initiation factor 1A	*	translation
PAAG_00747	eukaryotic translation initiation factor 2 subunit gamma	*	translation
PAAG_01330	eukaryotic translation initiation factor 3 RNA-binding subunit	*	translation
PAAG_00815	eukaryotic translation initiation factor 3 subunit A	*	translation
PAAG_02837	eukaryotic translation initiation factor 3 subunit H	*	translation
PAAG_04958	eukaryotic translation initiation factor 6	*	translation
PAAG_02071	glutamyl-tRNA synthetase	*	aminoacyl-tRNA-synthetases
PAAG_01786	phenylalanyl-tRNA synthetase beta chain	*	translation
PAAG_08025	tRNA (uracil-5-)-methyltransferase TRM9	*	tRNA modification
**PROTEIN FATE**			
PAAG_02497	WD repeat domain 5B	*	Ubl conjugation pathway
PAAG_01926	26S protease regulatory subunit 6A	*	ubiquitin-dependent protein proteolysis
PAAG_05943	26S proteasome non-ATPase regulatory subunit 12	*	assembly of proteasome
PAAG_01706	26S proteasome regulatory subunit RPN10	*	protein/peptide degradation
PAAG_08020	26S proteasome regulatory subunit rpn-8	*	protein/peptide degradation
PAAG_07037	calnexin	*	protein folding and stabilization
PAAG_01962	proteasome 26S subunit	*	protein/peptide degradation
PAAG_08184	T-complex protein 1 epsilon subunit	*	protein folding
PAAG_07165	T-complex protein 1 subunit gamma	*	protein folding
PAAG_01588	SNARE Ykt6	*	protein targeting, sorting and translocation
PAAG_04327	ubiquitin carboxyl-terminal hydrolase	*	protein deubiquitination
PAAG_03932	ubiquitin-activating enzyme E1 Y	*	protein ubiquitination
**BINDING**			
PAAG_03941	G4 quadruplex nucleic acid binding protein	*	RNA binding
**TRANSPORT**			
PAAG_08487	MIT family metal ion transporter	*	ion transport (cobalt)
PAAG_05135	tetratricopeptide repeat protein 1	*	potassium transport
PAAG_04904	ATP-binding cassette sub-family F member 2	*	ABC transport
PAAG_03644	mitochondrial import receptor subunit tom-40	*	protein transport
PAAG_02306	vacuolar H+\/Ca2+ exchanger	*	ion transport
PAAG_07900	phosphatidylinositol-phosphatidylcholine transfer protein (SEC14)	*	protein transport
**SIGNAL TRANSDUCTION**			
PAAG_08028	GTP-binding protein ypt1	*	protein transport/signal transduction process
PAAG_02458	GTP-binding protein ypt7	*	protein transport/signal transduction process
PAAG_08992	type 2A phosphatase activator tip41	*	signal transduction
**CELL RESCUE, DEFENSE AND VIRULENCE**			
PAAG_05679	heat shock protein	−3.45	stress response_protein folding
PAAG_07990	tetracycline transporter	*	antibiotic resistence
PAAG_01465	carbonic anhydrase	*	stress oxidative response/carbon utilization
PAAG_03216	mitochondrial peroxiredoxin PRX1	*	oxidative stress response
**CELL GROWTH/MORPHOGENESIS**			
PAAG_06370	sphingolipid long chain base-responsive protein LSP1	*	endocytosis
**MISCELLANEOUS**			
PAAG_04908	NAD binding Rossmann fold oxidoreductase	*	oxidoreductase
PAAG_02354	serine 3-dehydrogenase	*	serine 3-dehydrogenase activity

a Identification of differentially regulated proteins from *Paracoccidioides* genome database (http://www.broadinstitute.org/annotation/genome/paracoccidioides_brasiliensis/MultiHome.html) using the ProteinLynx Global Server (PLGS) version 3.0 (Waters Corporation. Manchester. UK);

b Proteins annotation from *Paracoccidioides* genome database or by homology in NCBI database (http://www.ncbi.nlm.nih.gov/);

c Protein expression profiles in **log2**-fold change (**5×-threshold**) obtained from ProteinLynx Global Server (PLGS) analysis normalized with internal standard.

d Biological process of differentially expressed proteins from MIPS (http://pedant.helmholtz-muenchen.de/pedant3htmlview/pedant3view?Method=analysis&Db=p3_r48325_Par_brasi_Pb01) and Uniprot database (http://www.uniprot.org/).

*: identified only in the presence of glucose (carbon condition).

### A comparative analysis of the transcriptome and proteome of *Paracoccidioides* under carbon starvation

To compare similar aspects between transcriptome and proteome data, we sought the same transcripts and proteins detected by both analyses. The transcripts and proteins identities (ID), from the *Paracoccidioides*, *Pb*01, database, were shown including their levels of abundance (Tables S7, S8, S9 and S10). Fifty seven identities (IDs) were matched of which 32 and 17 of them presented the same abundance profile, up- or down-regulated in both data, respectively (Tables S7 and S8). In this way, approximately 86% of the matches showed the same pattern of transcript and protein levels. On the other hand, the minority of IDs showed discrepancy in their abundance. Several of the transcripts in these groups were decreased in abundance, while the protein levels were increased and *vice – versa* (Tables S9 and S10).

**Figure 6:**
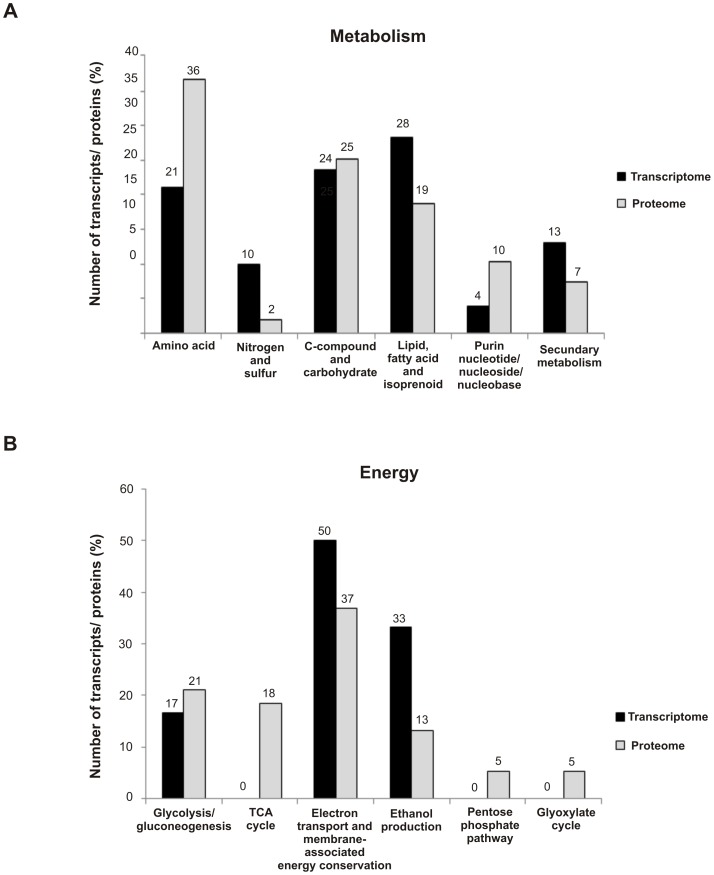
Number of transcripts and proteins related to metabolism and energy subcategories regulated in *Paracoccidioides*, *Pb*01, under carbon starvation. The number of transcripts and proteins, in percentage (%), regulated in *Paracoccidioides*, *Pb*01, under carbon starvation was calculated based on number of transcripts/proteins in each category shown in Figures S3 and S9, panels A. (A) Metabolism. The subcategories were represented by: amino acid; nitrogen/sulfur; C- compound and carbohydrates; lipid/fatty acid, and isoprenoid; purin nucleotide/nucleoside/nucleobase; secondary and phosphate metabolism. (B) Energy. The subcategories were represented by glycolysis/gluconeogenesis; TCA cycle; electron transport and membrane associated energy; ethanol production; pentose phosphate pathway and glyoxylate cycle. Black and gray bars indicate genes and proteins, respectively.

A comparative analysis including all transcripts and proteins for metabolism and energy categories from RNAseq and NanoUPLC-MS^E^ analysis was also performed (Fig. 6). Metabolism, which was the most regulated category in our data and energy are considered essential categories for understanding the carbon flow used by *Pb*01during carbon starvation. The results show that the amino acid, carbohydrate/C-compound, and lipid metabolism were similarly regulated in both approaches, showing the consistency with the suggested carbon flow in *Paracoccidioides*, *Pb*01, under carbon starvation (Figs. 3 and 6A). Amino acids and lipids are supposed to be intensively degraded (Tables S3 and S5) suggesting the production of precursors during carbon starvation, which include acetyl-CoA, pyruvate, oxaloacetate and succinate. Furthermore, the percentage of transcripts and proteins related to energy categories such as glycolysis/gluconeogenesis, electron transport/membrane associated energy conservation, and TCA cycle are also similar, in accordance with suggested responses of *Pb*01 to carbon starvation (Fig. 6B). Thus, the induction of gluconeogenesis, β-oxidation, part of TCA, and glyoxylate cycles was required to compensate for the absence of glucose and depicts the rearrangement of pathways when a carbon source condition is changed.

### Fungus-macrophage interaction

**Figure 7:**
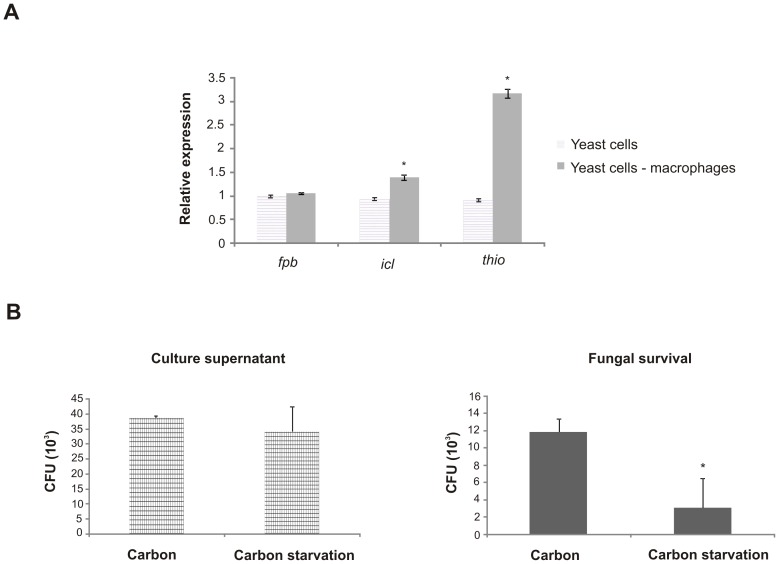
Expression of *Paracoccidioides fbp*, *icl* and *thio* genes and susceptibility of yeast cells to macrophages killing during infection. (**A**) *Pb*01 yeast cells were grown without (yeast cells) and with macrophages (yeast cells-macrophages) for 24 h in RPMI medium, and the relative expression of genes *fbp* (fructose-1,6-biphosphatase), *icl* (isocitrate lyase), and *thio* (3-ketoacyl-CoA thiolase) was determined. The data were normalized using the constitutive gene encoding the 60S ribosomal L34 gene as the endogenous control and are presented as relative expression in comparison to the experimental control cells value set at 1. (**B**) *Pb*01 yeast cells were previously grown in MMcM medium with carbon (4% of glucose) or absence of glucose (carbon starvation) up to 48 h and then were incubated with macrophages at a 1∶2.5 macrophages: yeast ratio, for both conditions. As demonstrated, the number of viable cells was determined by quantifying the number of colony forming units/mL (CFUs/mL) during infection from culture supernatant (non-internalized cells removed by aspiration prior to macrophages lysis) and after internalization. Data are expressed as the mean ± standard deviation of the biological triplicates of independent experiments. Student's *t*-test was used. *, significantly different from the control, at a p-value of ≤0.05.

We investigated the response to macrophages in *Paracoccidioides, Pb*01, under carbon starvation. We analyzed whether the fungus differentially expresses genes involved in gluconeogenesis, glyoxylate cycle, and β-oxidation pathways after internalization by the J744 A.1 macrophages. The relative expression analysis of transcripts encoding fructose-1,6-biphosphatase, isocitrate lyase and 3-ketoacyl CoA thiolase was performed using qRT-PCR. The Fig. 7A demonstrates that genes encoding isocitrate lyase and 3-ketoacyl CoA thiolase were induced (p≤0.05), suggesting a response of *Paracoccidioides* to carbon starvation in phagosomes. Furthermore, whether yeast cells under carbon starvation were more susceptible to macrophage killing than cells growing in plentiful glucose was analyzed. Firstly, plating of recovered yeast cells by aspiration of culture supernatant (non-internalized yeast cells) and from lysis of macrophages (internalized yeast cells) was performed (Fig. 7B). The result showed that the number of yeast cells recovered from culture supernatant was not significantly different between carbon and carbon starved yeast cells (Fig. 7B, on the left). On the contrary, the *Paracoccidioides*, *Pb*01, yeast cells pre-exposed to carbon starvation were recovered in a lower number than those grown under carbon source (Fig. 7B, on the right). In addition, to verify if the yeast cells were, in fact, more susceptible to macrophage killing we evaluated the average number of adhered/internalized *Paracoccidioides*, *Pb*01, using the light microscopy after 6 and 24 h of infection (Fig. S10 and S11). After 24 h of infection, it was observed, in carbon-starved condition, a significant decrease in the number of yeast cells adhered/internalized by macrophages (Fig. S10). Overall, the data suggest that yeast cells pre-exposed to carbon starvation were more susceptible to macrophage killing, reinforcing our suggestion that the carbon starvation can affect the survival of the *Pb*01 yeast cells inside of the macrophages.

## Discussion

The major focus of this work is directed towards a global view on the responses of *Paracoccidioides*, *Pb*01, to carbon starvation using both, high-throughput transcriptome and proteomic analysis. *Pb*01 yeast cells were able to adapt to carbon starving conditions. The data presented in this study reflected how carbon-starved cells modulate the metabolism by induction or repression of cellular activities. We show that the fungus regulates pathways that lead to glucose production to compensate the effect of stress. The fungus regulates transcripts and proteins that are mainly associated with gluconeogenesis and ethanol production *via* precursors from β-oxidation, glyoxylate and tricarboxylic acid cycles. Our study presents a detailed response of *Paracoccidioides* spp. facing carbon starvation and contributes to investigations of the importance of alternative carbon adaptation during fungus pathogenesis.

Changes in the kinetics of expression of representatives of gluconeogenesis, the glyoxylate cycle, and β-oxidation as well as the differential expression of isocitrate lyase at the protein level could establish a better time-point for our transcriptional (6 h) and proteomic (48 h) analysis, using RNAseq and NanoUPLC-MS^E^, respectively (Fig. 1). The transcriptome and proteomic analysis demonstrated that general metabolism and energy were the most represented regulated categories. Transcripts/proteins classified in energy and cell rescue, defense, and virulence categories were also induced in both approaches although less representative than metabolism (Fig. S3 and S9). Categories involved in the cell cycle, transcription, cellular transport, growth/morphogenesis, and signal transduction were predominantly down-regulated at transcription and protein levels. Although *Pb*01yeast cells displayed no significant difference in cell viability until 72 h under carbon starvation (Fig. S4), the yeast biomass was significantly reduced in this condition (Fig. 2). In addition, the abundance of specific transporters was elevated such as those related to copper, hexose, and monosaccharide uptake, suggesting a nutrient limitation and a hostile environment as detected in other fungi [60].

*Paracoccidioides*, *Pb*01, presented a complex mechanism to respond to nutrient deprivation. The suggestion is that the fungus uses a carbon flow (Fig. 3) through classical biochemical pathways such as glyoxylate cycle, β-oxidation, and gluconeogenesis which is in accordance with previous data on other fungi facing nutrient deprivation [8], [9], [12]. Fungi such as *C. albicans* and *C. neoformans* appear to experience a nutrient limited and stressful environment in the context of interaction with host cells. The elevated expression of the glyoxylate pathway and gluconeogenesis genes during *Cryptococcus* interactions with host tissue and phagocytic cells is similar to the regulation observed in *C. albicans*. These pathogenic fungi present a niche dependent metabolism, with activation of an alternative carbon source consuming process and the up-regulation of transcripts for enzymes of the glyoxylate cycle, β-oxidation, and gluconeogenesis [9], [12] which was also detected in *Paracoccidioides*, *Pb*01. Our data suggest that *Pb*01 yeast cells facing carbon starvation use the oxaloacetate molecule as a key intermediate of gluconeogenesis. It is possibly supported by β-oxidation and, in part, by glyoxylate and TCA cycles activation. The glyoxylate cycle can allow the fungus to assimilate two-carbon compounds, a relevant aspect to the viability and growth inside macrophages [61], [62]. Studies involving the isocitrate lyase gene, representative of glyoxylate cycle, displayed that this gene is important as a marker for gluconeogenic carbon source utilization and starvation rather than a marker for lipid metabolism [63], [64]. Despite induction of isocitrate lyase and genes required for fatty acid utilization especially after phagocytosis by macrophages [9], this induction may derive from a general stress response due to nutrient or glucose limitation rather than a specific induction from fatty acid utilization [64]. In fact, null mutants to isocitrate lyase in *C. albicans*, *A. fumigatus* and *C. neoformans* and for β-oxidation genes in *C. albicans* revealed no virulence defects, showing that fatty acids do not provide an essential nutrient source during infection [2], [64]–[67]. In addition, isocitrate lyase activity increased when *C. albicans* was subjected to carbon starvation or other carbon sources such as acetate, glutamate and peptone as solely carbon source [63] reinforcing its importance as a marker for gluconeogenic carbon source utilization and starvation. Moreover, the isocitrate dehydrogenase enzyme was detected as down-regulated in our proteomic data (Table S6). The activities of isocitrate dehydrogenase and isocitrate lyase enzymes, regulate the flow of isocitrate into either the tricarboxylic acid cycle or the glyoxylate cycle [68], [69]. Here, while the isocitrate dehydrogenase is down, the isocitrate lyase is up-regulated, in accordance with suggested carbon flow in *Pb*01 through glyoxylate shunt. As the same importance, the increased expression of protein phosphoenolpyruvate carboxykinase confirms that gluconeogenesis process is ongoing (Table S5). Then, the global characterization of the responses of *Pb*01 to carbon starvation becomes relevant in this context especially by flow of carbon used by the fungus during this stress.

The same transcripts and proteins detected by both analyses were identified (Tables S7, S8, S9 and S10). Approximately 86% of the matches (a total of 57) showed the same pattern of transcript and protein levels. In this way, we believe that the transcriptional and proteome time-points were enough to characterize the global responses of the *Paracoccidioides* to carbon starvation conditions. Similarly to previous study in *A. fumigatus*, few times, transcripts and proteins do not follow the same trend of expression that could be explained, for example, by mRNA stabilization process or by active post-transcriptional and translational regulatory mechanisms [70]. In our data, in many metabolic aspects, transcripts and protein levels were correlated. The identities to amino acid degradation, β-oxidation and ethanol synthesis were increased in expression while processes involving the pyruvate molecule were down-regulated (Table S7 and S8) corroborating our hypothesis of a well established response to carbon starved environments (Fig. 3). The pyruvate-acetyl-CoA conversion, for example, is diminished by repression of pyruvate dehydrogenase enzyme. In addition, pyruvate carboxylase is repressed and it is possibly not converted in oxaloacetate. These observations strongly suggest that the available pyruvate would end up in ethanol *via* acetaldehyde (Fig. 3).

In addition, comparison of regulated molecules in transcriptome and proteome data with focus in metabolism and energy categories can support the use of alternative carbon sources by *Pb*01 under carbon starvation (Fig. 6). Metabolism of amino acids, lipids, and carbohydrate/C-compound was the most regulated in both used approaches (Fig. 6A). The amino acids and lipids likely are been used as precursors to important molecules involved in alternative carbon metabolism in *Pb*01 as depicted in Fig. 3. In fact, *Paracoccidioides* spp. can use a relatively wide range of amino acids and peptides rather than carbohydrates [21]. In addition to the structural importance of lipids, these molecules provide an energy-rich nutrient source. β-oxidation is a common pathway for the utilization of fatty acids [71] in which of the 3-ketoacyl-CoA thiolases enzymes are important [64]. Recent studies have highlighted the relevance of the β-oxidation in response to nutrient or glucose limitation rather than a specific induction from fatty acid utilization. In fact, fatty acids do not provide an essential nutrient source during infection in *C. albicans* but is important for coupling the glyoxylate cycle and fatty acid β-oxidation during host-pathogen interactions, regulating responses related to carbon starvation [63], [64]. Here, the induction of β-oxidation pathway in *Pb*01 likely reflects the requirement of this metabolic pathway for carbon starving adaptation, which is consistent with previous data in *C. albicans* subjected to a poor-nutrition environment [9]. Regarding energy producing pathways, the gluconeogenesis, tricarboxylic acid and glyoxylate cycles are well represented, which reinforces our model of adaptation to carbon starvation conditions (Fig. 6B). Moreover, the electron transport and ethanol subcategories were also shown. Ethanol metabolism was previously described in *Pb*01 showing evidence for a more anaerobic metabolism of this fungus compared with other isolates of *Paracoccidioides* [35], [36], [72], and this metabolite has also been described as relevant to pathogenic fungi such as *A. fumigatus* [70], [73], [74].

The general response to oxidative stress mediated by enzymes provides multiple resistance strategies to *Paracoccidioides* yeast cells [34]. There was a high increase in production of antioxidant proteins such as thioredoxin reductase, superoxide dismutase and cytochrome c peroxidase that are possibly involved in defense against reactive oxygen species (ROS) during carbon starvation (Tables S5 and 1). In low amounts, ROS are generated continuously as side products of aerobic respiration in the mitochondria and, although potentially cytotoxic, function as signal molecules in cellular processes [75], [76]. In this way, the production of ROS during carbon starvation could be related to the increase in electron transport activity in respiratory chain (Table S5 and 1) as well as to the production of endogenous free radicals in β-oxidation.

The response of *Pb*01 to macrophage infection shows that the fungus most likely faces carbon starvation in macrophages, because a significant higher expression of genes encoding isocitrate lyase and 3-ketoacyl-CoA thiolase was detected. It is important to note the similarities in the transcriptional profile in inducing alternate carbon metabolism between *C. albicans* phagocytosed cells and those submitted to carbon starvation (39). In fact, in terms of usable nutrients, the phagosome has been reported to not have a rich environment evidenced by the unsubstantial quantities of glucose, other sugars and amino acids [9], [11], [12], [27], [77]. Here, we showed that *Pb*01 yeast cells were more susceptible to macrophage killing when were previously starved of carbon (Fig. 7, S10 and S11). This conclusion is based on the fact that the multiplication of *Paracoccidioides* inside activated macrophages is inhibited [23]. In this sense, we suggested that *Paracoccidioides*, *Pb*01, yeast cells pre-exposed to carbon starvation, were more susceptible to macrophage killing, reinforcing that carbon deprivation affects the survival of the *Pb*01 yeast cells inside of the macrophages.

Taken together, our data suggest that *Pb*01 changes its metabolism under carbon starvation reprogramming several biological processes to facilitate its maintenance under this condition. These programs are mainly related to gluconeogenesis, β-oxidation and the glyoxylate cycle to compensate for the starved carbon environment. Considering a new perspective, the transcriptome and proteome data could reinforce the responses of this fungus, which is able to survive in the hostile environment during macrophage infection. This study may elucidate potential molecules involved in host-fungus interactions, an important factor related to pathogenic organisms.

## Supporting Information

Figure S1 **Initial effects of carbon starvation in** ***Paracoccidioides*** **yeast cells protein expression.** (**A**) Proteins (50 µg) of *Pb*01 yeast cells were incubated at 36°C in MMcM medium with (4%) or without (0%) of glucose for 0, 12, 24, 30 and 48 h. The abundance of *Pb*Icl was analyzed by western blotting. The proteins were fractionated by one-dimensional gel electrophoresis. The proteins were blotted onto a nitrocellulose membrane and the ∼60 kDa protein species was detected by using the rabbit polyclonal antibody anti-*Pb*Icl [43]. (**B**) Densitometric analysis of immunoblotting bands was performed using the software AphaEaseFC. The difference in *Pb*Icl expression was just observed in 48 h under carbon starvation.
(TIF)

Figure S2 **Global analysis of RNAseq data.** Mapped reads data were analyzed by DEGseq package and plotting graphs were obtained. The transcripts are represented by dots. (A) Scatter plot shows the number of reads (log2) counts for each transcript in CS (carbon-starved) and C (carbon) conditions. (B) MA-plot of CS *versus* C conditions shows the intensity of expression of identified transcripts (log2 of fold change) in the *y* axe [M] and the read counts (log2) for each transcript in the *x* axe [A]. In addition, the graph shows the number of differentially expressed transcripts obtained from FET (Fisher Exact Test) using a p-value of 0.001 in red color.
(TIF)

Figure S3 **Functional classification and abundance levels of transcripts regulated in** ***Paracoccidioides*** **under carbon starvation obtained by RNAseq.** (**A**) Biological processes of differentially expressed transcripts in *Paracoccidioides*, *Pb*01, under carbon starvation are shown. The biological processes were obtained using the Pedant on MIPS (http://pedant.helmholtz-muenchen.de/pedant3htmlview/pedant3view?Method=analysis&Db=p3_r48325_Par_brasi_Pb01) and Uniprot database (http://www.uniprot.org/). A total of 1,063 transcripts are shown represented by the percentage (%) of regulated unclassified (64.6%) and classified (35.4%) transcripts for each category. (**B**) The number of classified up- and down-regulated transcripts in *Paracoccidioides*, *Pb*01, under carbon starvation stress is shown for each category depicted in (A). A total of 190 and 186 transcripts were up- and down-regulated and are depicted by light and dark gray colors bars, respectively. The percentages (%) show the number of up- and down-regulated transcripts for each category based on a total of them.
(TIF)

Figure S4 **Cell viability of** ***Paracoccidioides*** **subjected to carbon and carbon starvation conditions.** The effect of carbon (4% glucose) and carbon starvation (0% glucose) conditions in viability of *Pb*01 yeast cells was investigated using flow citometry. The viability was determined by a membrane integrity analysis using propidium iodide (1 µg/mL) as a dead cell marker in a C6 Accuri flow cytometer (Accuri Cytometers, Ann Arbor, MI, USA). The experiments were performed in triplicate. Statistical analyses was performed using Student's t-test; all of the samples showed p-values<0.05 (*) and were considered statistically significant. The errors bars represent the standard deviation of three biological replicates.
(TIF)

Figure S5 **Formamidase activity assay.** The activity was determined by the measuring the amount of ammonia formation at 37°C. A total of 1 µg of each total protein extract of *Paracoccidioides* under carbon (4% glucose) and carbon starvation (0% glucose) conditions for 6, 24, 48 and 72 h in MMcM medium was used. One unit (U) of formamidase specific activity was defined as the amount of enzyme required to hydrolyze 1 µmol of formamide (corresponding to the formation of 1 µmol of ammonia) per min per mg of total protein and are represented as U.mg^−1^. Errors bars represent standard deviation from three biological replicates while * represents p≤0.05.
(TIF)

Figure S6 **Peptide detection type to carbon and carbon-starved samples.** The pie graph show the percentage of peptides matched against the *Paracoccidioides* (*Pb*01) database by PLGS (PepFrag 1 and PepFrag 2), variables modifications (VarMod), fragmentation that occurred on ionization source (InSource), missed cleavage performed by trypsin (Missed Cleavage) and Neutral loss H_2_O and NH_3_ correspondent to water and ammonia precursor losses to carbon (A) and carbon starvation (B) conditions. The SpotFire Decision Site 8.0 v program was used. The PepFrag parameters should be predominant, in contrast to insource and missed cleavage which should not reach 20%.
(TIF)

Figure S7 **Peptide mass accuracy analyzes.** Peptides data were used to make the bar graph showing the accuracy of mass for peptides in carbon and carbon starvation samples. A total of 94.9 and 95.7% of identified peptides were detected in a 15 ppm error range in both samples, carbon (A) and carbon starvation (B), respectively.
(TIF)

Figure S8 **Detection of dynamic range of proteomic analyzes.** The dynamic range the proteomic experiments for each condition was determined. Graphs for carbon (A) and carbon-starved (B) are shown. Regular, reverse, and standard proteins were indicated by gray/square, blue/circle and yellow/triangle colors/shape, respectively. The regular and reverse proteins indicate identified proteins using regular and reverse genomic database from *Paracoccidioides*, *Pb*01, respectively. The standard protein was used to normalize the expression data and compare the carbon and carbon-starved proteins. Our data showed an acceptable quantification to standard protein between the both conditions.
(TIF)

Figure S9 **Functional classification and abundance levels of proteins regulated in** ***Paracoccidioides*** **under carbon starvation obtained by NanoUPLC-MS^E^ data.** (**A**) Biological processes of differentially expressed proteins in *Paracoccidioides*, *Pb*01, under carbon starvation are shown. The biological processes were obtained using the Pedant on MIPS (http://pedant.helmholtz-muenchen.de/pedant3htmlview/pedant3view?Method=analysis&Db=p3_r48325_Par_brasi_Pb01) and Uniprot databases (http://www.uniprot.org/). Three hundred and thirty-five proteins are represented by the percentage (%) in each category. (**B**) The number of up- and down-regulated proteins in *Paracoccidioides*, *Pb*01, under carbon starvation is shown for each category depicted in (A). Two hundred and three proteins were up- regulated (dark gray) and one hundred and thirty-two proteins were down-regulated and are depicted by the light gray color bar.
(TIF)

Figure S10 **Number of internalized/adhered** ***Paracoccidioides*** **yeast cells.** The average number of adhered/internalized *Paracoccidioides* cells by macrophages was determined. Macrophages were infected with *Paracoccidioides* yeast cells which were pre-cultivated under carbon and carbon starvation conditions by 6 and 24 h. A total of 300 macrophages were counted for each time and condition and the number of adhered/internalized fungal cells was shown in percentage of the total as the mean value ± the standard deviation from triplicates. The statistical analyses were performed using Student's t test.
(TIF)

Figure S11 **Microscopy of adhered/internalized** ***Paracoccidioides*** **yeast cells by macrophages.** The *Paracoccidioides* yeast cells pre-cultivated in the presence of carbon and under carbon starvation were co-incubated with macrophages for 6 and 24 h. The arrows and double arrows indicate internalized and adhered yeast cells, respectively. The cells were fixed with methanol, stained by Giemsa and visualized via light microscopy (magnification 100×, oil immersion), as detailed in the Materials and Methods section.
(TIF)

Table S1 **Candidate reference genes for normalization according to expression stability calculated by Normfinder.**
(DOC)

Table S2 **Specifics primers used in qRT-PCR.**
(DOC)

Table S3 **Up-regulated transcripts of** ***Paracoccidioides*** **(** ***Pb*** **01) yeast cells under carbon starvation detected by RNAseq analysis.**
(DOC)

Table S4 **Down-regulated transcripts of** ***Paracoccidioides*** **(** ***Pb*** **01) yeast cells under carbon starvation detected by RNAseq analysis.**
(DOC)

Table S5 **Up-regulated proteins of** ***Paracoccidioides*** **(** ***Pb*** **01) yeast cells under carbon starvation detected using NanoUPLC-MS^E^.**
(DOC)

Table S6 **Down-regulated proteins of** ***Paracoccidioides*** **(** ***Pb*** **01) yeast cells under carbon starvation detected using NanoUPLC-MS^E^.**
(DOC)

Table S7 **Up-regulated proteins and transcripts of** ***Paracoccidioides*** **(** ***Pb*** **01) yeast cells under carbon starvation detected by NanoUPLC-MS^E^ and RNAseq analysis.**
(DOC)

Table S8 **Down-regulated proteins and transcripts of** ***Paracoccidioides*** **(** ***Pb*** **01) yeast cells under carbon starvation detected by NanoUPLC-MS^E^ and RNAseq analysis.**
(DOC)

Table S9 **Up- and down-regulated proteins and transcripts, respectively, of** ***Paracoccidioides*** **(** ***Pb*** **01) yeast cells under carbon starvation detected by NanoUPLC-MS^E^ and RNAseq analysis.**
(DOC)

Table S10 **Down- and up-regulated proteins and transcripts, respectively, of** ***Paracoccidioides*** **(** ***Pb*** **01) yeast cells under carbon starvation detected by NanoUPLC-MS^E^ and RNAseq analysis.**
(DOC)
