# Supplementary figures and images for: Transcriptional and Proteomic Responses to Carbon Starvation in Paracoccidioides
Source: PLoS Negl Trop Dis. 2014 May 8;8(5):e2855. doi: 10.1371/journal.pntd.0002855 (PMC4014450; doi:10.1371/journal.pntd.0002855)

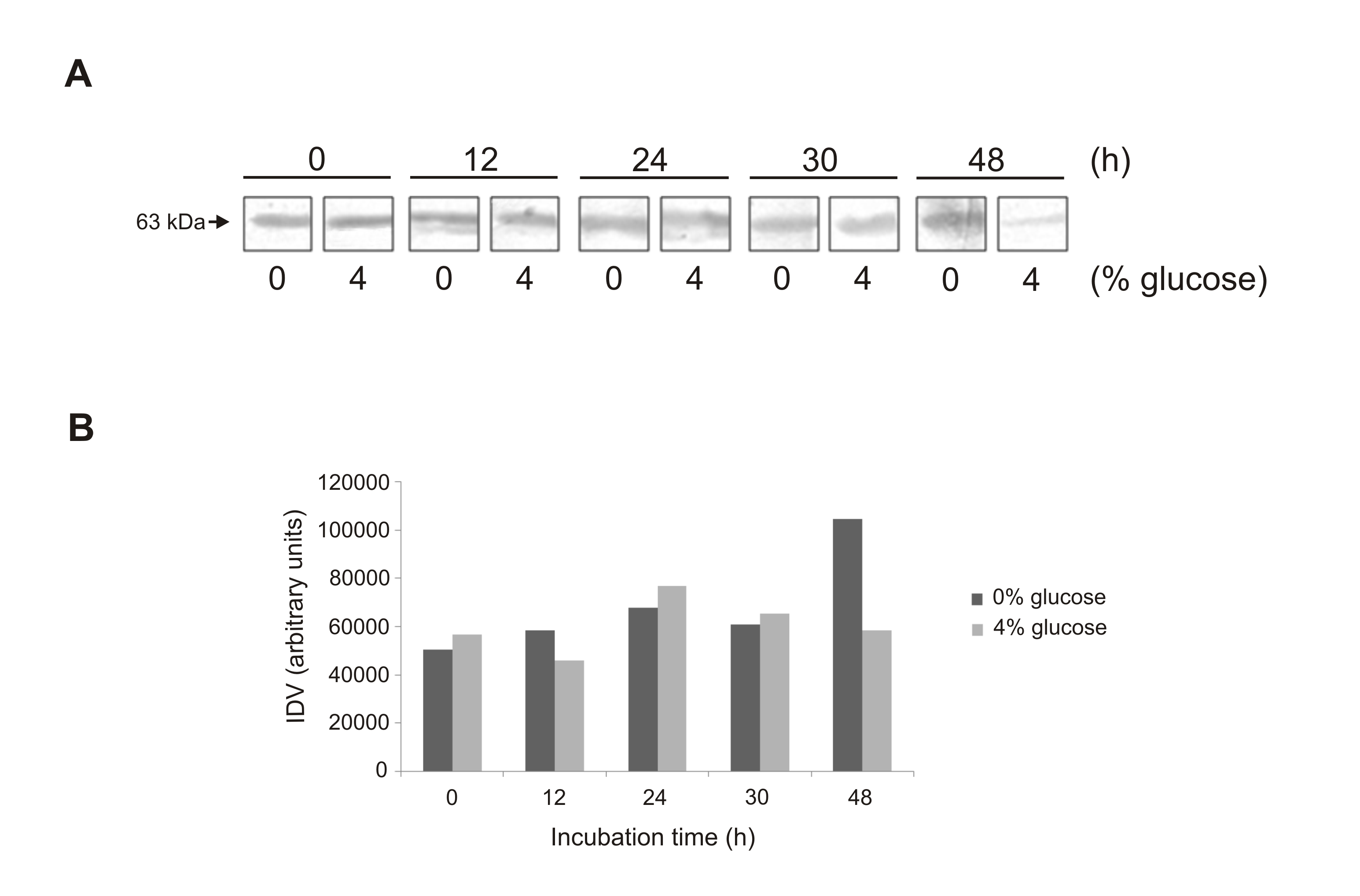

Supplement: Figure S1 — Initial effects of carbon starvation in Paracoccidioides yeast cells protein expression. (A) Proteins (50 µg) of Pb01 yeast cells were incubated at 36°C in MMcM medium with (4%) or without (0%) of glucose for 0, 12, 24, 30 and 48 h. The abundance of PbIcl was analyzed by western blotting. The proteins were fractionated by one-dimensional gel electrophoresis. The proteins were blotted onto a nitrocellulose membrane and the ∼60 kDa protein species was detected by using the rabbit polyclonal antibody anti-PbIcl [43]. (B) Densitometric analysis of immunoblotting bands was performed using the software AphaEaseFC. The difference in PbIcl expression was just observed in 48 h under carbon starvation. (TIF) [file pntd.0002855.s001.tif]

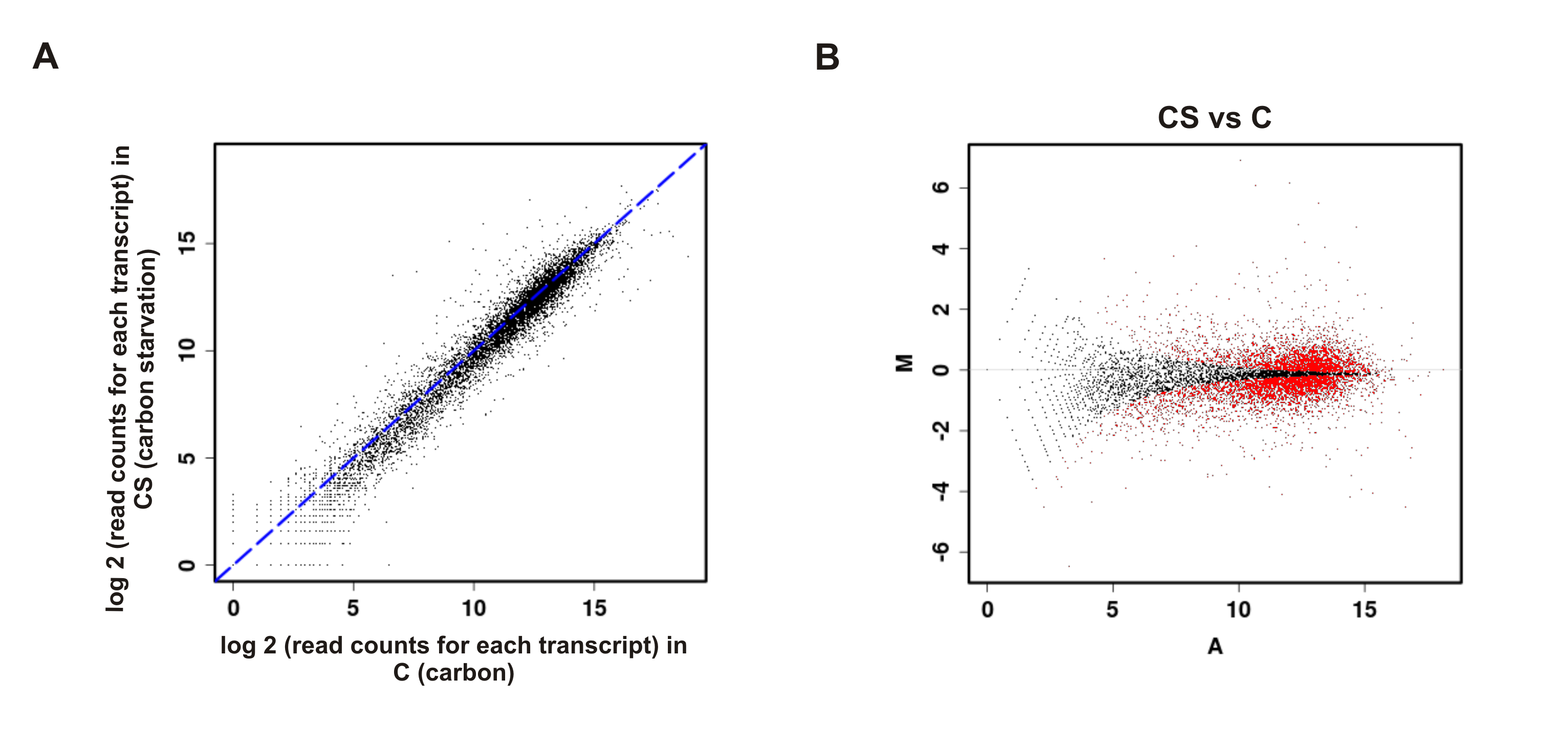

Supplement: Figure S2 — Global analysis of RNAseq data. Mapped reads data were analyzed by DEGseq package and plotting graphs were obtained. The transcripts are represented by dots. (A) Scatter plot shows the number of reads (log2) counts for each transcript in CS (carbon-starved) and C (carbon) conditions. (B) MA-plot of CS versus C conditions shows the intensity of expression of identified transcripts (log2 of fold change) in the y axe [M] and the read counts (log2) for each transcript in the x axe [A]. In addition, the graph shows the number of differentially expressed transcripts obtained from FET (Fisher Exact Test) using a p-value of 0.001 in red color. (TIF) [file pntd.0002855.s002.tif]

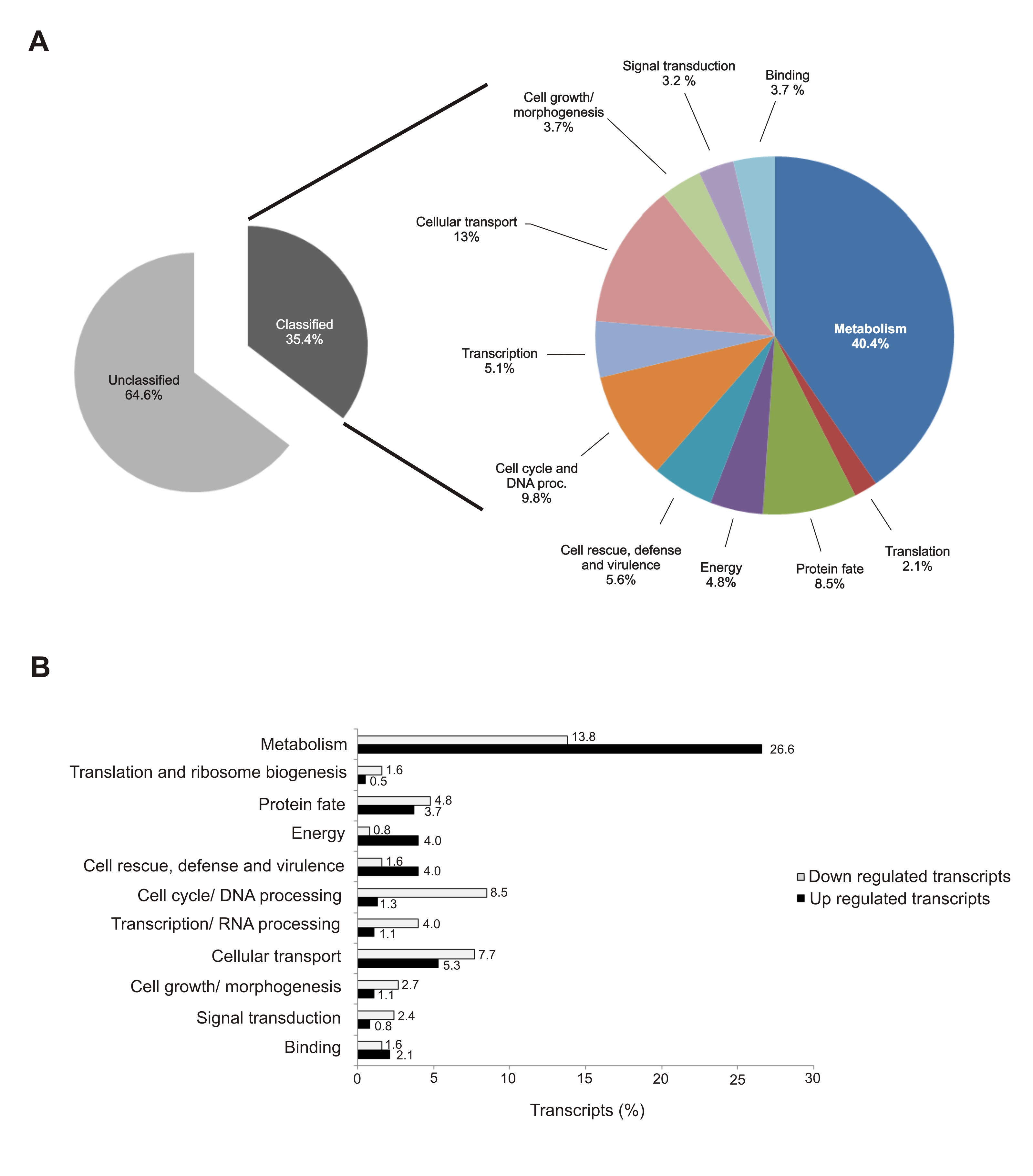

Supplement: Figure S3 — Functional classification and abundance levels of transcripts regulated in Paracoccidioides under carbon starvation obtained by RNAseq. (A) Biological processes of differentially expressed transcripts in Paracoccidioides, Pb01, under carbon starvation are shown. The biological processes were obtained using the Pedant on MIPS (http://pedant.helmholtz-muenchen.de/pedant3htmlview/pedant3view?Method=analysis&Db=p3_r48325_Par_brasi_Pb01) and Uniprot database (http://www.uniprot.org/). A total of 1,063 transcripts are shown represented by the percentage (%) of regulated unclassified (64.6%) and classified (35.4%) transcripts for each category. (B) The number of classified up- and down-regulated transcripts in Paracoccidioides, Pb01, under carbon starvation stress is shown for each category depicted in (A). A total of 190 and 186 transcripts were up- and down-regulated and are depicted by light and dark gray colors bars, respectively. The percentages (%) show the number of up- and down-regulated transcripts for each category based on a total of them. (TIF) [file pntd.0002855.s003.tif]

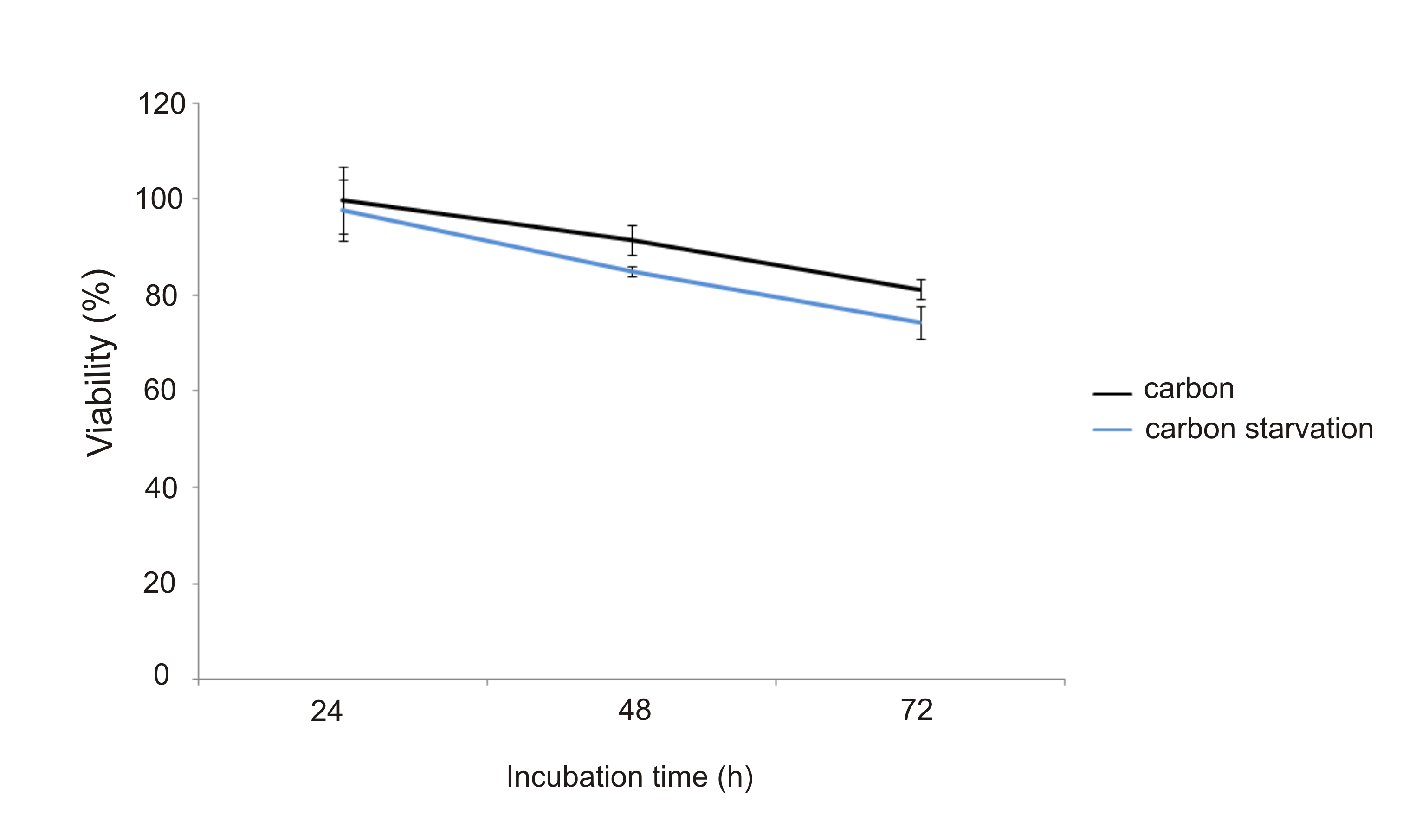

Supplement: Figure S4 — Cell viability of Paracoccidioides subjected to carbon and carbon starvation conditions. The effect of carbon (4% glucose) and carbon starvation (0% glucose) conditions in viability of Pb01 yeast cells was investigated using flow citometry. The viability was determined by a membrane integrity analysis using propidium iodide (1 µg/mL) as a dead cell marker in a C6 Accuri flow cytometer (Accuri Cytometers, Ann Arbor, MI, USA). The experiments were performed in triplicate. Statistical analyses was performed using Student's t-test; all of the samples showed p-values<0.05 (*) and were considered statistically significant. The errors bars represent the standard deviation of three biological replicates. (TIF) [file pntd.0002855.s004.tif]

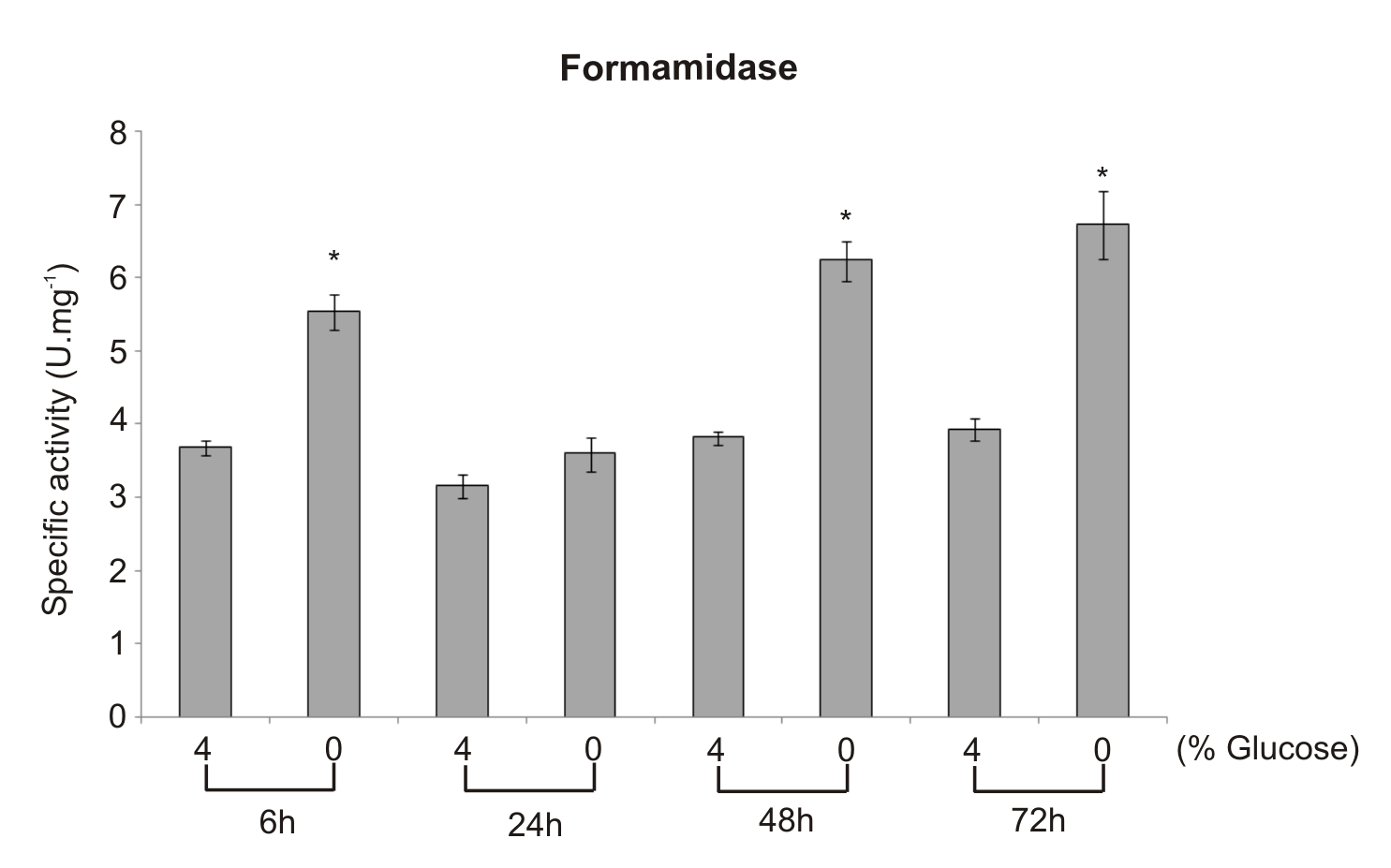

Supplement: Figure S5 — Formamidase activity assay. The activity was determined by the measuring the amount of ammonia formation at 37°C. A total of 1 µg of each total protein extract of Paracoccidioides under carbon (4% glucose) and carbon starvation (0% glucose) conditions for 6, 24, 48 and 72 h in MMcM medium was used. One unit (U) of formamidase specific activity was defined as the amount of enzyme required to hydrolyze 1 µmol of formamide (corresponding to the formation of 1 µmol of ammonia) per min per mg of total protein and are represented as U.mg−1. Errors bars represent standard deviation from three biological replicates while * represents p≤0.05. (TIF) [file pntd.0002855.s005.tif]

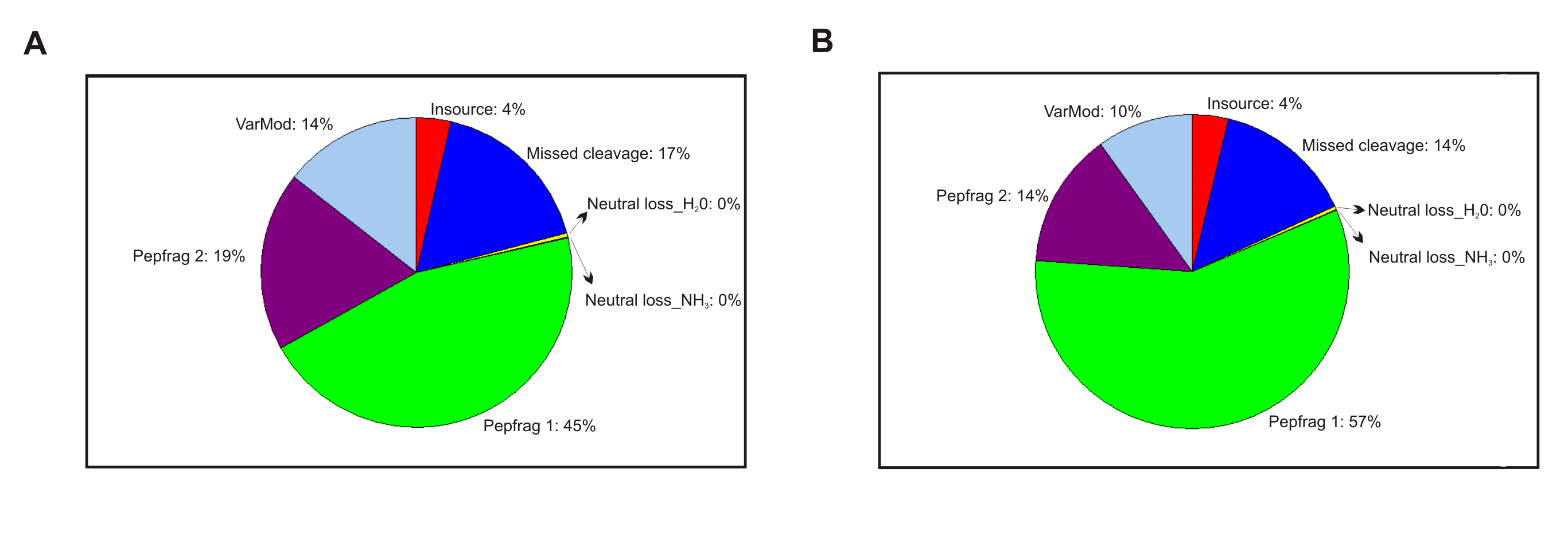

Supplement: Figure S6 — Peptide detection type to carbon and carbon-starved samples. The pie graph show the percentage of peptides matched against the Paracoccidioides (Pb01) database by PLGS (PepFrag 1 and PepFrag 2), variables modifications (VarMod), fragmentation that occurred on ionization source (InSource), missed cleavage performed by trypsin (Missed Cleavage) and Neutral loss H2O and NH3 correspondent to water and ammonia precursor losses to carbon (A) and carbon starvation (B) conditions. The SpotFire Decision Site 8.0 v program was used. The PepFrag parameters should be predominant, in contrast to insource and missed cleavage which should not reach 20%. (TIF) [file pntd.0002855.s006.tif]

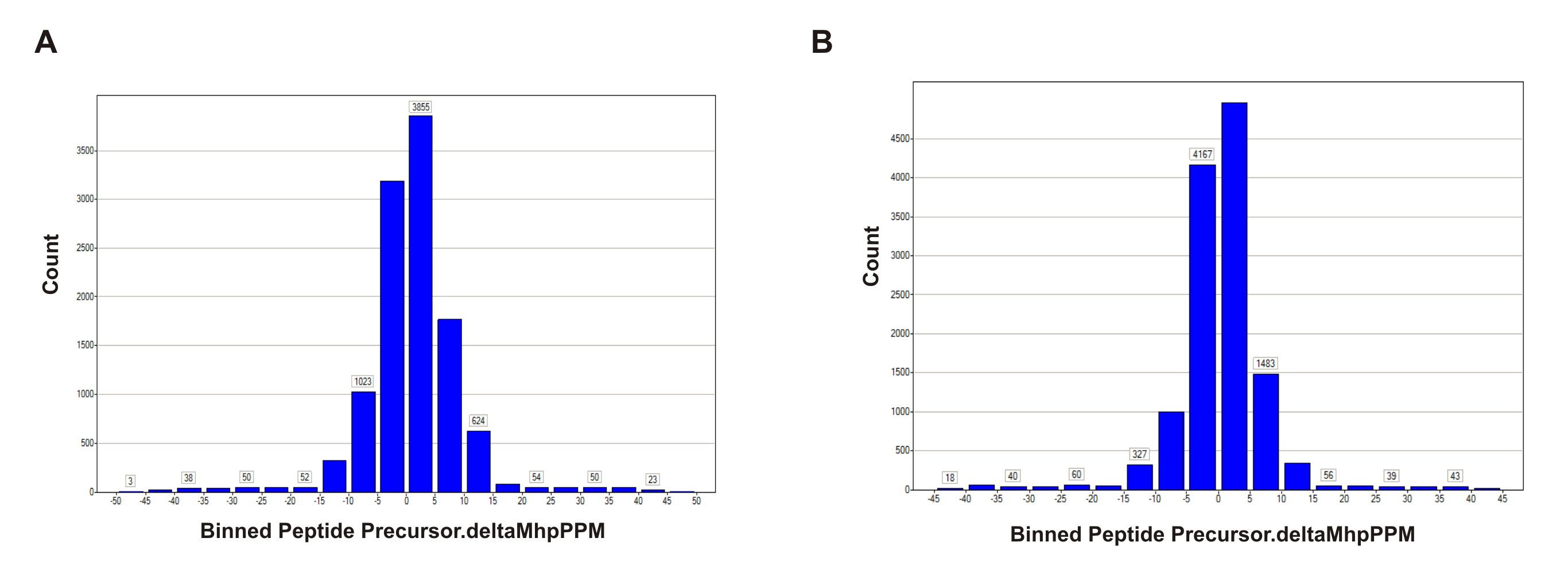

Supplement: Figure S7 — Peptide mass accuracy analyzes. Peptides data were used to make the bar graph showing the accuracy of mass for peptides in carbon and carbon starvation samples. A total of 94.9 and 95.7% of identified peptides were detected in a 15 ppm error range in both samples, carbon (A) and carbon starvation (B), respectively. (TIF) [file pntd.0002855.s007.tif]

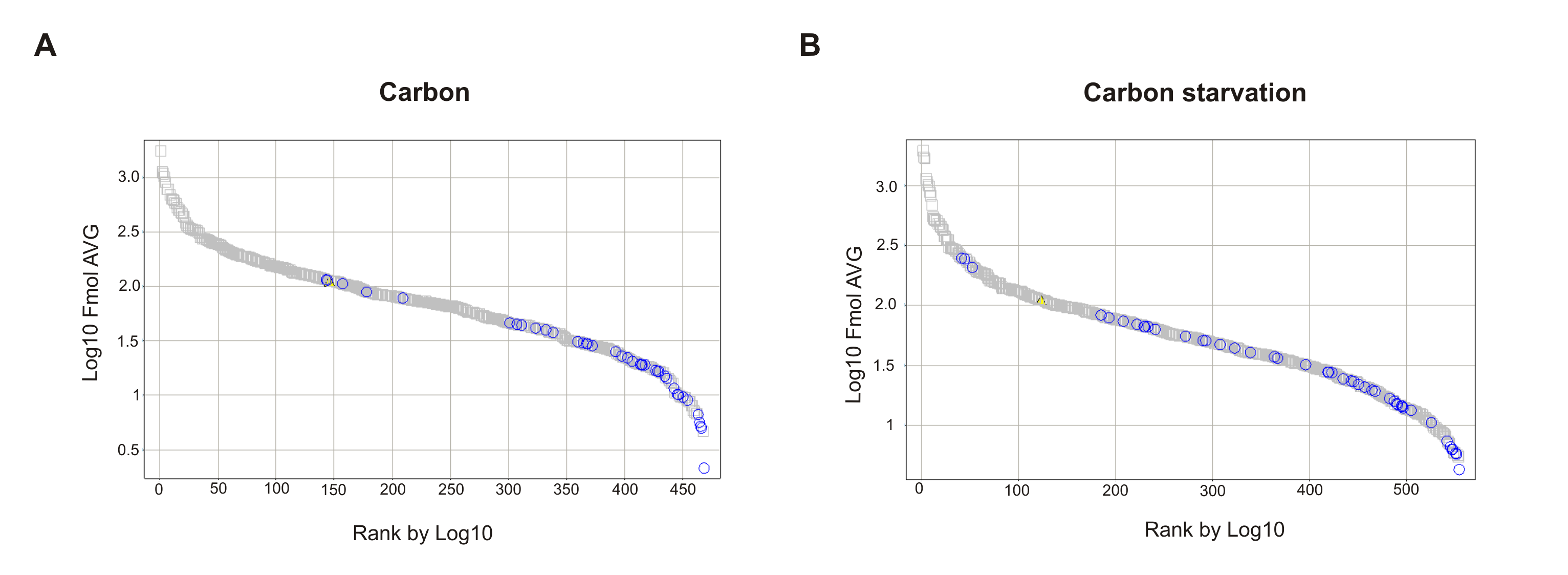

Supplement: Figure S8 — Detection of dynamic range of proteomic analyzes. The dynamic range the proteomic experiments for each condition was determined. Graphs for carbon (A) and carbon-starved (B) are shown. Regular, reverse, and standard proteins were indicated by gray/square, blue/circle and yellow/triangle colors/shape, respectively. The regular and reverse proteins indicate identified proteins using regular and reverse genomic database from Paracoccidioides, Pb01, respectively. The standard protein was used to normalize the expression data and compare the carbon and carbon-starved proteins. Our data showed an acceptable quantification to standard protein between the both conditions. (TIF) [file pntd.0002855.s008.tif]

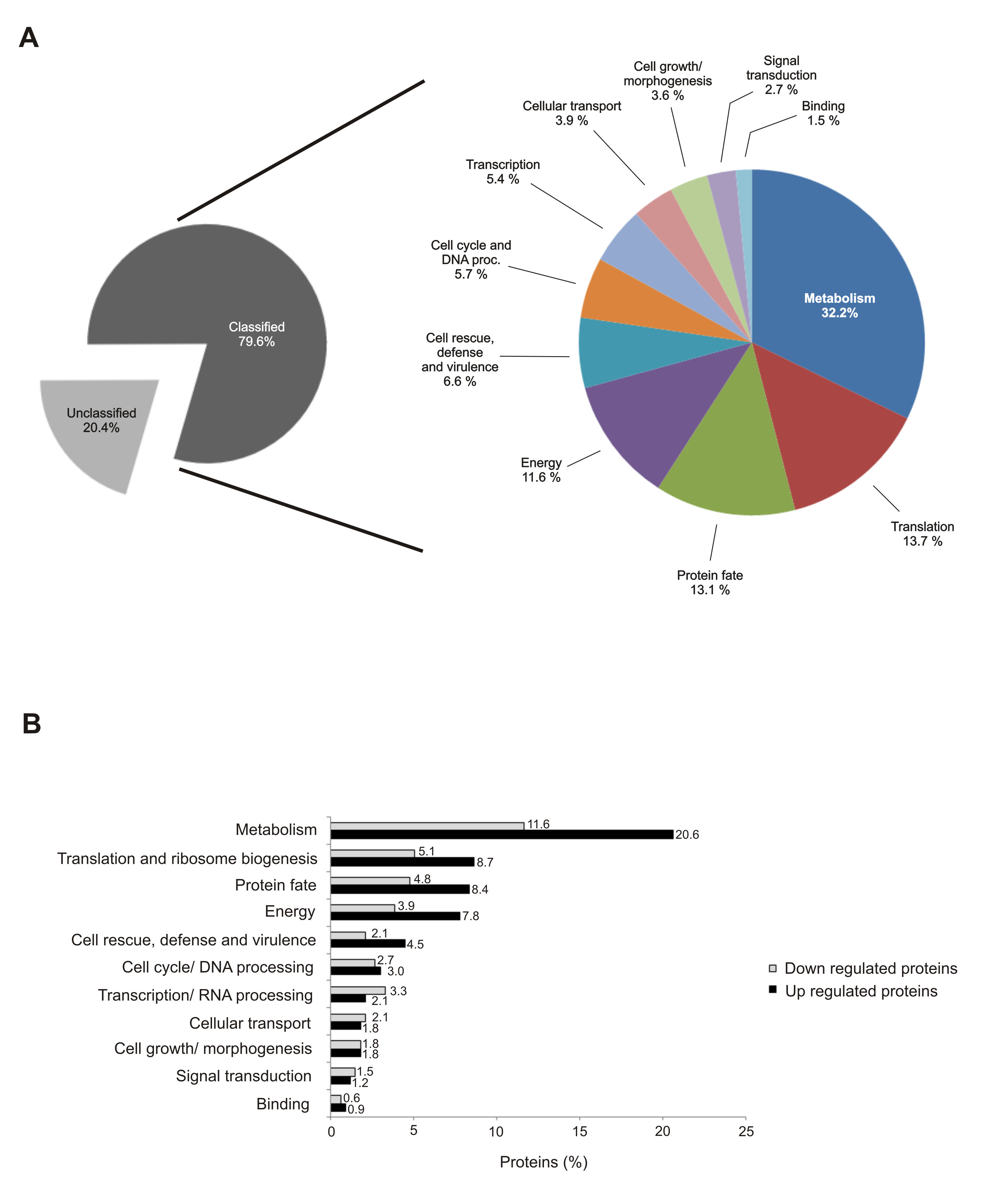

Supplement: Figure S9 — Functional classification and abundance levels of proteins regulated in Paracoccidioides under carbon starvation obtained by NanoUPLC-MSE data. (A) Biological processes of differentially expressed proteins in Paracoccidioides, Pb01, under carbon starvation are shown. The biological processes were obtained using the Pedant on MIPS (http://pedant.helmholtz-muenchen.de/pedant3htmlview/pedant3view?Method=analysis&Db=p3_r48325_Par_brasi_Pb01) and Uniprot databases (http://www.uniprot.org/). Three hundred and thirty-five proteins are represented by the percentage (%) in each category. (B) The number of up- and down-regulated proteins in Paracoccidioides, Pb01, under carbon starvation is shown for each category depicted in (A). Two hundred and three proteins were up- regulated (dark gray) and one hundred and thirty-two proteins were down-regulated and are depicted by the light gray color bar. (TIF) [file pntd.0002855.s009.tif]

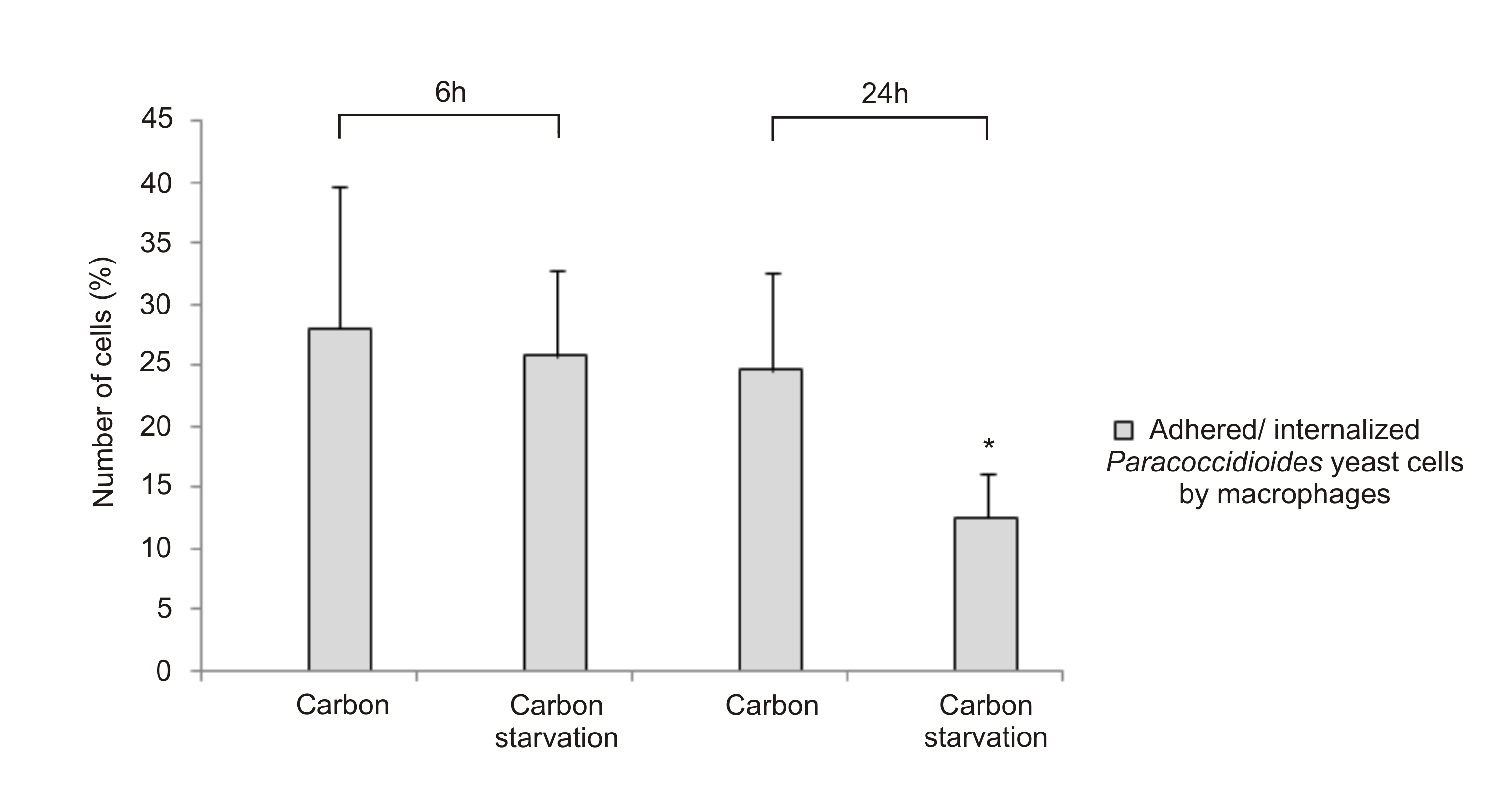

Supplement: Figure S10 — Number of internalized/adhered Paracoccidioides yeast cells. The average number of adhered/internalized Paracoccidioides cells by macrophages was determined. Macrophages were infected with Paracoccidioides yeast cells which were pre-cultivated under carbon and carbon starvation conditions by 6 and 24 h. A total of 300 macrophages were counted for each time and condition and the number of adhered/internalized fungal cells was shown in percentage of the total as the mean value ± the standard deviation from triplicates. The statistical analyses were performed using Student's t test. (TIF) [file pntd.0002855.s010.tif]

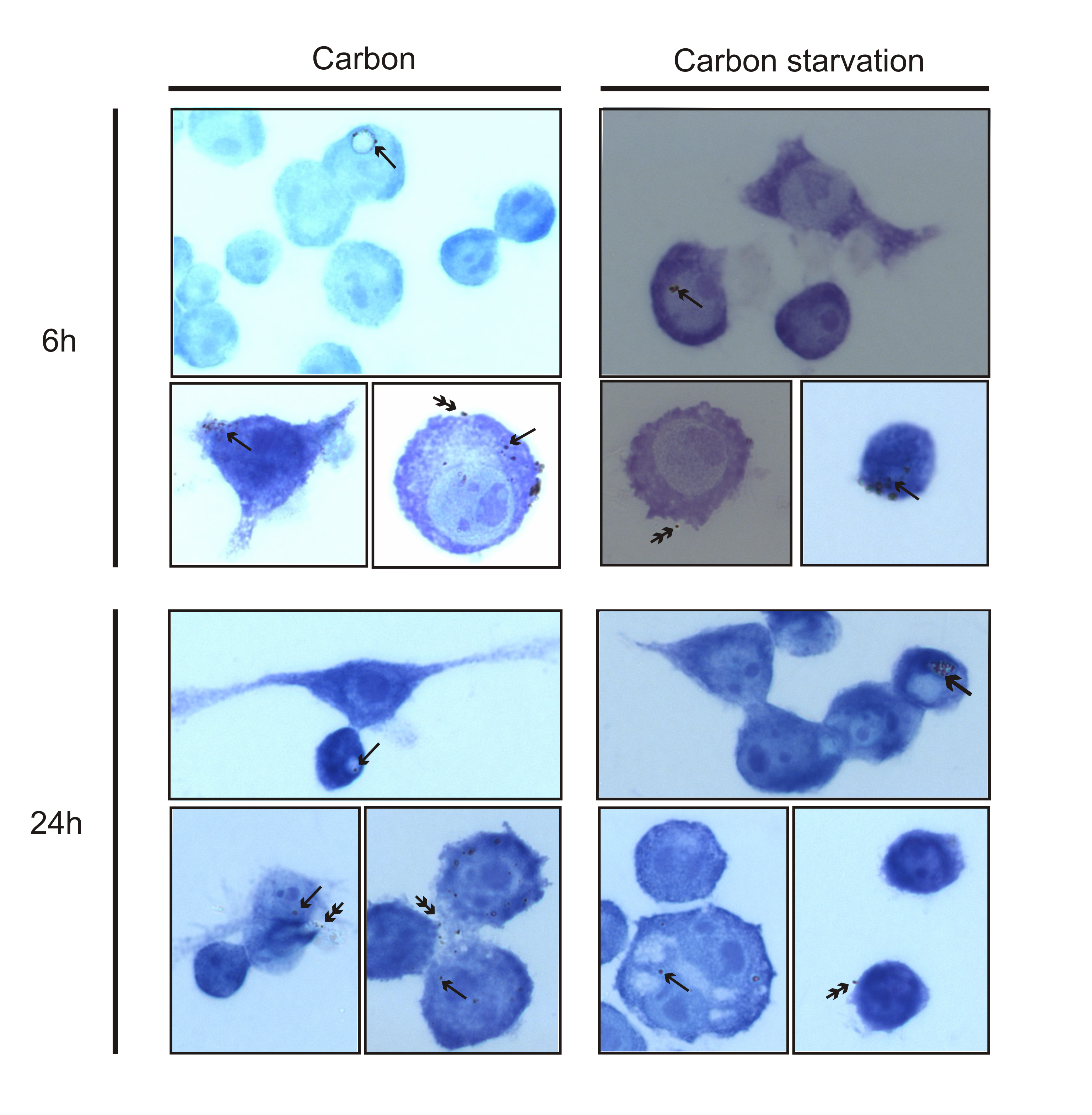

Supplement: Figure S11 — Microscopy of adhered/internalized Paracoccidioides yeast cells by macrophages. The Paracoccidioides yeast cells pre-cultivated in the presence of carbon and under carbon starvation were co-incubated with macrophages for 6 and 24 h. The arrows and double arrows indicate internalized and adhered yeast cells, respectively. The cells were fixed with methanol, stained by Giemsa and visualized via light microscopy (magnification 100×, oil immersion), as detailed in the Materials and Methods section. (TIF) [file pntd.0002855.s011.tif]
