# Supplementary material for: Transcriptional and Proteomic Responses to Carbon Starvation in Paracoccidioides
Source: PLoS Negl Trop Dis. 2014 May 8;8(5):e2855. doi: 10.1371/journal.pntd.0002855 (PMC4014450; doi:10.1371/journal.pntd.0002855)
Supplement: Table S1 — Candidate reference genes for normalization according to expression stability calculated by Normfinder. (DOC) [file pntd.0002855.s012.doc]

**Table S1. Candidate reference genes for normalization according to expression stability calculated by Normfinder.**

| **Rank order** | **Gene^a^** | **Stability value^b^**  **first run** | **Stability value^b^**  **second run** |
| --- | --- | --- | --- |
| **1** | L34 | 0.279 | 0.319 |
| **2** | TUB | 0.803 | 0.710 |
| **3** | ACT | 0.636 | 0.697 |

^a^ L34, 60S ribosomal protein (PAAG_00746); TUB, tubulin alpha-1 chain (PAAG_01647) and ACT, actin (PAAG_00564) were choosed to find the best candidate for use as normalizer in our qRT-PCRs analysis. The accession numbers are from *Paracoccidioides* genome database (<http://www.broadinstitute.org/annotation/genome/paracoccidioides_brasiliensis/MultiHome.html>).

^b^ Estimated by combination of the intragroup and intergroup expression variation given a stability value that enables the ranking of genes by expression stability. The best normalizer gene shows a low stability value.
