# Supplementary material for: Transcriptional and Proteomic Responses to Carbon Starvation in Paracoccidioides
Source: PLoS Negl Trop Dis. 2014 May 8;8(5):e2855. doi: 10.1371/journal.pntd.0002855 (PMC4014450; doi:10.1371/journal.pntd.0002855)
Supplement: Table S2 — Specifics primers used in qRT-PCR. (DOC) [file pntd.0002855.s013.doc]

**Table S2.** **Specifics primers used in qRT-PCR.**

| **Gene/ Accession number^a^** | **Forward primer (5´→3´)** | **Reverse primer (5´→3´)** |
| --- | --- | --- |
| *fructose-1,6-biphosphatase* / PAAG_02682 | GCCACTGGTGACTTTACCCT | CATCTCCGGTGGTATTTGCG |
| *isocitrate lyase* / PAAG_06951 | ATGGGAACCGACCTCCTGG | CGTTCTTGCCTGCTTGCTCA |
| *3-ketoacyl-CoA thiolase* / PAAG_07746 | CAGATTACAGATGGAGCCGC | GGTTGTACATCGAGAGCAGC |
| *60S ribosomal protein L34/* PAAG_00746 | TCAATCTCTCCCGCGAATCC | AGTTGGCGATTGTTGTGCGG |
| *tubulin alpha-1 chain* / PAAG_01647 | ACAGTGCTTGGGAACTATACC | GGGACATATTTGCCACTGCC |
| *actin* / PAAG_00564 | CACAAGGTCTCAGCAGTGGT | GGGCGATTAGGTTACGAGTG |

^a^ Accession number of genes used in quantitative real time PCR from *Paracoccidioides* genome database (<http://www.broadinstitute.org/annotation/genome/paracoccidioides_brasiliensis/MultiHome.html>).
