# Supplementary material for: Transcriptional and Proteomic Responses to Carbon Starvation in Paracoccidioides
Source: PLoS Negl Trop Dis. 2014 May 8;8(5):e2855. doi: 10.1371/journal.pntd.0002855 (PMC4014450; doi:10.1371/journal.pntd.0002855)
Supplement: Table S3 — Up-regulated transcripts of Paracoccidioides ( Pb 01) yeast cells under carbon starvation detected by RNAseq analysis. (DOC) [file pntd.0002855.s014.doc]

**Table S3. Up-regulated transcripts of *Paracoccidioides* (*Pb*01) yeast cells under carbon starvation detected by RNAseq analysis.**

|  | **ID^a^** | **Annotation^b^** | **Fold change (log2)^c^** | **Biological process^d^** |
| --- | --- | --- | --- | --- |
| **METABOLISM** | | | | |
| **Amino acid metabolism** | | | | |
|  | PAAG_03032 | delta-1-pyrroline-5-carboxylate dehydrogenase | 1.67 | glutamate biosynthesis |
|  | PAAG_01365 | choline dehydrogenase | 1.64 | glycine biosynthesis |
|  | PAAG_08900 | 2,2-dialkylglycine decarboxylase | 1.64 | metabolism of alanine |
|  | PAAG_03402 | pyridoxal-5'-phosphate-dependent enzyme | 1.57 | threonine biosynthesis |
|  | PAAG_02924 | asparagine synthase | 1.51 | asparagine biosynthesis |
|  | PAAG_00580 | tyrosinase central domain-containing protein | 1.41 | tyrosine degradation |
|  | PAAG_03143 | dihydrodipicolinate synthase | 1.40 | lysine biosynthesis |
|  | PAAG_04757 | 3-hydroxyanthranilate 3,4-dioxygenase | 1.39 | tryptophan degradation |
|  | PAAG_08162 | maleylacetoacetate isomerase | 1.35 | phenylalanine and tyrosine degradation |
|  | PAAG_05743 | aromatic amino acid aminotransferase | 1.29 | methionine biosynthesis |
|  | PAAG_04525 | glutamine synthetase | 1.26 | glutamine biosynthesis |
|  | PAAG_05253 | delta-1-pyrroline-5-carboxylate dehydrogenase | 1.26 | glutamate biosynthesis |
|  | PAAG_00966 | L-threonine 3-dehydrogenase | 1.20 | threonine degradation |
|  | PAAG_08649 | cysteine dioxygenase | 1.15 | cysteine degradation |
|  | PAAG_06404 | aspartate aminotransferase | 1.12 | arginine biosynthesis |
|  | PAAG_02229 | Guanidinobutyrase | 1.11 | proline biosynthesis |
|  | PAAG_06387 | homoisocitrate dehydrogenase | 1.03 | isoleucine biosynthesis |
| **Nitrogen and sulfur metabolismo** | | | | |
|  | PAAG_03333 | Formamidase | 4.39 | nitrogen, sulfur and selenium metabolism |
|  | PAAG_08904 | nitrogen metabolic regulation protein | 2.82 | regulation of nitrogen, sulfur and selenium metabolism |
|  | PAAG_04596 | Acetamidase | 1.96 | nitrogen, sulfur and selenium metabolism |
|  | PAAG_03626 | Acetamidase | 1.79 | nitrogen, sulfur and selenium metabolism |
|  | PAAG_05769 | succinate-semialdehyde dehydrogenase | 1.78 | nitrogen metabolism |
|  | PAAG_06693 | 2-nitropropane dioxygenase | 1.47 | nitrogen, sulfur and selenium metabolism |
|  | PAAG_01692 | copper-containing nitrite reductase | 1.44 | nitrogen metabolism |
|  | PAAG_05161 | Acetamidase | 1.17 | nitrogen, sulfur and selenium metabolism |
|  | PAAG_04233 | 2-nitropropane dioxygenase | 1.07 | nitrogen, sulfur and selenium metabolism |
| **C-compound and carbohydrate metabolism** | | | | |
|  | PAAG_02509 | 2-amino-3-carboxymuconate-6-semialdehyde decarboxylase | 2.81 | C-compound and carbohydrate metabolism |
|  | PAAG_02162 | lactam utilization protein LamB | 2.54 | C-compound and carbohydrate metabolism |
|  | PAAG_03765 | NADP-dependent glycerol dehydrogenase | 2.53 | sugar, glucoside, polyol and carboxylate catabolism |
|  | PAAG_05416 | NADP-dependent leukotriene B4 12-hydroxydehydrogenase | 2.43 | C-compound and carbohydrate metabolism |
|  | PAAG_03249 | high-affinity hexose transporter HXT6 | 1.87 | C-compound and carbohydrate metabolism |
|  | PAAG_06863 | mannitol dehydrogenase | 1.78 | C-compound and carbohydrate metabolism |
|  | PAAG_02653 | acetyl-coenzyme A synthetase | 1.67 | C-compound metabolism/ Acetyl-coA synthesis |
|  | PAAG_05254 | alpha-galactosidase | 1.47 | C-compound and carbohydrate metabolism |
|  | PAAG_03625 | 3-oxoacyl-[acyl-carrier-protein] reductase | 1.34 | C-compound and carbohydrate metabolism |
|  | PAAG_02103 | aldose 1-epimerase | 1.33 | C-compound and carbohydrate metabolism |
|  | PAAG_05649 | endochitinase | 1.30 | aminosaccharide anabolism |
|  | PAAG_05995 | glucose oxidase | 1.22 | C-compound and carbohydrate metabolism |
|  | PAAG_07909 | carboxylic acid transport protein | 1.22 | C-compound and carbohydrate metabolism |
|  | PAAG_04888 | 4-coumarate-CoA ligase | 1.21 | C-compound and carbohydrate metabolism |
|  | PAAG_02770 | thermoresistant gluconokinase | 1.19 | C-compound and carbohydrate metabolism |
|  | PAAG_05124 | endoplasmic reticulum mannosyl-oligosaccharide 1,2-alpha-mannosidase | 1.17 | C-compound and carbohydrate metabolism |
|  | PAAG_05377 | xylitol dehydrogenase | 1.15 | C-compound and carbohydrate metabolism |
|  | PAAG_07735 | PfkB family carbohydrate kinase (Mak32) | 1.12 | carbohydrate metabolism |
|  | PAAG_06843 | alpha-1,2-mannosyltransferase | 1.10 | C-compound and carbohydrate metabolism |
|  | PAAG_01060 | acetate non-utilizing protein | 1.07 | C-compound and carbohydrate metabolism |
|  | PAAG_01136 | beta-hexosaminidase | 1.03 | polysaccharide metabolism |
|  | PAAG_02350 | salicylate hydroxylase | 1.02 | C-compound catabolism |
|  | PAAG_00543 | alpha-mannosidase | 1.00 | C-compound and carbohydrate metabolism |
| **Lipid, fatty acid and isoprenoid metabolism** | | | | |
|  | PAAG_05782 | acyl-CoA dehydrogenase | 2.52 | lipid, fatty acid and isoprenoid metabolism |
|  | PAAG_05249 | aldehyde dehydrogenase | 2.42 | fatty acid metabolism/ acetate |
|  | PAAG_07721 | 2,5-dichloro-2,5-cyclohexadiene-1,4-diol dehydrogenase | 2.36 | fatty acid metabolism |
|  | PAAG_04313 | fatty acid transporter protein | 2.10 | lipid, fatty acid and isoprenoid metabolism |
|  | PAAG_02163 | acetyl-/propionyl-coenzyme A carboxylase alpha chain | 2.09 | lipid metabolism |
|  | PAAG_04615 | 3,2-trans-enoyl-CoA isomerase ,Delta(2)-enoyl-CoA isomerase | 1.96 | lipid, fatty acid and isoprenoid metabolism |
|  | PAAG_07014 | acyl-CoA dehydrogenase | 1.96 | lipid, fatty acid and isoprenoid metabolism |
|  | PAAG_01928 | peroxisomal dehydratase | 1.93 | lipid, fatty acid and isoprenoid metabolism |
|  | PAAG_04293 | lipase/esterase | 1.89 | lipid, fatty acid and isoprenoid metabolism |
|  | PAAG_07271 | carnitinyl-CoA dehydratase | 1.87 | lipid, fatty acid and isoprenoid metabolism |
|  | PAAG_02664 | 3-ketoacyl-CoA thiolase peroxisomal A | 1.77 | fatty acid metabolism |
|  | PAAG_03190 | short-chain specific acyl-CoA dehydrogenase | 1.73 | lipid, fatty acid and isoprenoid metabolism |
|  | PAAG_04894 | sporulation protein SPS19 | 1.49 | fatty acid catabolism |
|  | PAAG_06629 | thioesterase family protein | 1.43 | fatty acid metabolism |
|  | PAAG_07746 | 3-ketoacyl-CoA thiolase | 1.40 | lipid, fatty acid and isoprenoid metabolism |
|  | PAAG_08052 | long-chain-fatty-acid-CoA ligase | 1.37 | lipid, fatty acid and isoprenoid metabolism |
|  | PAAG_01683 | peroxisomal dehydratase | 1.37 | lipid, fatty acid and isoprenoid metabolism |
|  | PAAG_07347 | phytanoyl-CoA dioxygenase family protein | 1.31 | fatty acid metabolism |
|  | PAAG_08859 | peroxisomal multifunctional enzyme | 1.28 | lipid, fatty acid and isoprenoid metabolism |
|  | PAAG_09117 | long chain fatty acid oxidase | 1.25 | lipid, fatty acid and isoprenoid metabolism |
|  | PAAG_05376 | 3-oxoacyl-[acyl-carrier-protein] reductase | 1.25 | lipid, fatty acid and isoprenoid metabolism |
|  | PAAG_06031 | esterase | 1.23 | lipid, fatty acid and isoprenoid metabolism |
|  | PAAG_01273 | cholinesterase | 1.18 | lipid, fatty acid and isoprenoid metabolism |
|  | PAAG_03123 | 3-oxoacyl-(acyl-carrier-protein) reductase | 1.17 | fatty acid metabolism |
|  | PAAG_06392 | enoyl-CoA hydratase/isomerase family protein | 1.16 | lipid, fatty acid and isoprenoid metabolism |
|  | PAAG_04963 | oleate-induced peroxisomal protein | 1.14 | lipid, fatty acid and isoprenoid metabolism |
|  | PAAG_01387 | 3-ketoacyl-CoA thiolase peroxisomal B | 1.11 | lipid, fatty acid and isoprenoid metabolism |
|  | PAAG_05533 | GDSL lipase/acylhydrolase family protein | 1.07 | lipid and fatty acid metabolism |
|  | PAAG_07793 | enoyl-CoA hydratase | 1.03 | lipid, fatty acid and isoprenoid metabolism |
|  | PAAG_03618 | S-adenosylmethionine-dependent methyltransferase | 1.03 | lipid and fatty acid metabolism |
| **Purin nucleotide, nucleoside and nucleobase**  **metabolism** | | | | |
|  | PAAG_00523 | adenosine deaminase | 1.30 | nucleotide metabolism |
|  | PAAG_08856 | nicotinate-nucleotide pyrophosphorylase | 1.20 | biosynthesis of vitamins, cofactors, and prosthetic groups |
|  | PAAG_02333 | GMP synthase | 1.13 | purin nucleotide/nucleoside/nucleobase metabolism |
| **Secondary metabolism** | | | | |
|  | PAAG_04897 | 4-coumarate-CoA ligase | 2.67 | metabolism of phenylpropanoids |
|  | PAAG_08658 | copper amine oxidase | 2.32 | metabolism of amines |
|  | PAAG_01031 | riboflavin kinase | 1.72 | vitamin metabolism |
|  | PAAG_08664 | phenylacetate 2-hydroxylase | 1.61 | metabolism of phenylpropanoids |
|  | PAAG_07075 | hexaprenyldihydroxybenzoate methyltransferase | 1.35 | metabolism of ubiquinone |
|  | PAAG_00673 | NUDIX domain-containing protein | 1.31 | metabolism of vitamins, cofactors, and prosthetic groups |
|  | PAAG_02189 | Class II Aldolase family protein | 1.29 | metabolism of aminoglycoside antibiotics |
|  | PAAG_08831 | 2-dehydropantoate 2-reductase | 1.25 | biosynthesis of vitamins |
|  | PAAG_05427 | hydroxyquinol 1,2-dioxygenase | 1.24 | secondary metabolism |
|  | PAAG_03226 | 3-phytase A | 1.14 | secondary metabolism |
|  | PAAG_03755 | copper amine oxidase | 1.12 | metabolism of amines |
|  | PAAG_03208 | pantetheine-phosphate adenylyltransferase family protein | 1.08 | biosynthesis of vitamins, cofactors and prosthetic groups |
|  | PAAG_02336 | nudix hydrolase | 1.07 | metabolism of vitamins, cofactors and prosthetic groups |
|  | PAAG_06924 | glutamate-1-semialdehyde 2,1-aminomutase | 1.05 | biosynthesis of vitamins, cofactors and prosthetic groups |
|  | PAAG_05841 | nicotinamide n-methyltransferase | 1.02 | metabolism of vitamins, cofactors and prosthetic groups |
|  | PAAG_04981 | 3,4-dihydroxy-2-butanone 4-phosphate synthase | 1.01 | biosynthesis of vitamins, cofactors and prosthetic groups |
|  | PAAG_07164 | amidohydrolase | 1.01 | regulation of phosphate metabolism |
|  | PAAG_06485 | phosphomethylpyrimidine kinase | 1.00 | biosynthesis of vitamins, cofactors and prosthetic groups |
| **ENERGY** | | | | |
| **Glycolysis and gluconeogenesis** | | | | |
|  | PAAG_01995 | fructose-bisphosphate aldolase | 1.28 | glycolysis and gluconeogenesis |
|  | PAAG_06460 | triosephosphate isomerase | 1.06 | glycolysis and gluconeogenesis |
| **Electron transport and membrane-associated**  **energy conservation** | | | | |
|  | PAAG_01378 | cytochrome P450 52A3 | 2.48 | energy conversion and regeneration |
|  | PAAG_06351 | bifunctional P-450:NADPH-P450 reductase | 1.70 | electron transport |
|  | PAAG_02515 | cytochrome P450 55A1 | 1.67 | electron transport |
|  | PAAG_06984 | NADPH dehydrogenase | 1.64 | energy conversion and regeneration |
|  | PAAG_07984 | cytochrome b2 | 1.36 | energy generation |
|  | PAAG_06595 | 3-demethylubiquinone-9 3-methyltransferase | 1.04 | energy generation |
|  | PAAG_01137 | cytochrome P450 52A11 | 1.00 | electron transport |
| **Ethanol production** | | | | |
|  | PAAG_00403 | alcohol dehydrogenase | 3.92 | alcohol fermentation |
|  | PAAG_04541 | alcohol dehydrogenase | 2.77 | alcohol fermentation |
|  | PAAG_08903 | alcohol dehydrogenase | 2.30 | alcohol fermentation |
|  | PAAG_06715 | alcohol dehydrogenase | 1.65 | alcohol fermentation |
|  | PAAG_00243 | alcohol dehydrogenase IV | 1.48 | alcohol fermentation |
|  | PAAG_02512 | pyruvate decarboxylase | 1.33 | alcohol fermentation |
| **CELL CYCLE and DNA PROCESSING** | | | | |
|  | PAAG_00427 | DNA repair and transcription factor Ada | 2.03 | DNA repair |
|  | PAAG_06573 | endonuclease/exonuclease/phosphatase family protein | 1.77 | DNA repair |
|  | PAAG_04562 | C-5 cytosine methyltransferase DmtA | 1.21 | meiosis |
|  | PAAG_00897 | DNAJ domain-containing protein | 1.05 | mitotic cell cycle and cell cycle control |
|  | PAAG_02411 | pps1 dual specificity phosphatase | 1.00 | mitotic cell cycle and cell cycle control |
| **TRANSCRIPTION and RNA PROCESSING** | | | | |
|  | PAAG_06845 | CCCH zinc finger DNA binding protein | 2.02 | transcription |
|  | PAAG_00070 | RING finger domain-containing protein | 1.19 | transcription control |
|  | PAAG_03958 | C2H2 type zinc finger containing protein | 1.09 | transcriptional control |
|  | PAAG_00645 | SRF-type transcription factor RlmA | 1.02 | transcriptional control |
| **TRANSLATION and RIBOSSOME BIOGENESIS** | | | | |
|  | PAAG_02220 | eukaryotic translation initiation factor 5 | 1.07 | translation initiation |
|  | PAAG_02930 | translation initiation factor 4E | 1.03 | translation initiation |
| **PROTEIN FATE** | | | | |
|  | PAAG_05736 | ubiquitin-conjugating enzyme | 1.79 | modification by ubiquitination, deubiquitination |
|  | PAAG_08275 | phosphotransferase enzyme family protein | 1.38 | protein modification |
|  | PAAG_05661 | zinc ion binding | 1.32 | modification by ubiquitin-related proteins |
|  | PAAG_00526 | dual specificity protein phosphatase 3 | 1.30 | modification by phosphorylation, dephosphorylation, autophosphorylation |
|  | PAAG_00121 | two-component system protein A | 1.27 | modification by phosphorylation, dephosphorylation, autophosphorylation |
|  | PAAG_03619 | candidapepsin-4 | 1.24 | protein/peptide degradation |
|  | PAAG_02376 | serine/threonine-protein phosphatase PP-X isozyme 1 | 1.22 | modification by phosphorylation, dephosphorylation, autophosphorylation |
|  | PAAG_05466 | xaa-Pro dipeptidase | 1.19 | protein/peptide degradation |
|  | PAAG_04977 | ubiquitin-conjugating enzyme | 1.17 | modification by ubiquitination, deubiquitination |
|  | PAAG_06501 | superoxide dismutase 1 copper chaperone | 1.13 | protein folding and stabilization |
|  | PAAG_00258 | extracelular serine carboxypeptidase | 1.11 | protein processing (proteolytic) |
|  | PAAG_07131 | arginine N-methyltransferase | 1.09 | protein modification |
|  | PAAG_07315 | N-terminal amidase | 1.07 | protein modification |
|  | PAAG_01329 | carboxypeptidase S1 | 1.04 | protein/peptide degradation |
| **BINDING** | | | | |
|  | PAAG_07806 | benzoate 4-monooxygenase cytochrome P450 | 1.48 | metal binding |
|  | PAAG_06541 | erythrocyte band 7 integral membrane protein | 1.36 | protein binding |
|  | PAAG_00587 | dimethylaniline monooxygenase | 1.34 | FAD/FMN binding |
|  | PAAG_03261 | metallo-beta-lactamase family protein | 1.32 | metal binding |
|  | PAAG_07038 | APAF1-interacting protein | 1.24 | metal binding |
|  | PAAG_06909 | yippee zinc-binding protein Moh1 | 1.22 | heavy metal binding (Cu, Fe, Zn) |
|  | PAAG_03944 | cytosolic Fe-S cluster assembling factor NBP35 | 1.12 | nucleotide/nucleoside/nucleobase binding |
|  | PAAG_06446 | transcriptional regulatory protein pro-1 | 1.04 | DNA binding |
| **TRANSPORT** | | | | |
|  | PAAG_05251 | high affinity copper transporter | 3.81 | heavy metal ion transport (Cu+, Fe3+, etc,) |
|  | PAAG_03112 | MFS transporter (Mch2) | 2.82 | C-compound and carbohydrate transport |
|  | PAAG_03624 | Arp2/3 complex subunit Arc16 | 2.46 | endocytosis |
|  | PAAG_00811 | MFS monocarboxylate transporter | 1.90 | transport |
|  | PAAG_05916 | aquaporin-7 | 1.70 | channel / pore class transport |
|  | PAAG_00486 | transporter of Nicotinic Acid | 1.60 | vitamine/cofactor transport |
|  | PAAG_01453 | high-affinity glucose transporter | 1.57 | C-compound and carbohydrate transport |
|  | PAAG_09034 | general amino-acid permease GAP1 | 1.52 | amino acid/amino acid derivatives transport |
|  | PAAG_03452 | carnitine/acyl carnitine carrier | 1.47 | lipid/fatty acid transport |
|  | PAAG_07313 | zinc-regulated transporter 1 | 1.44 | heavy metal ion transport (Cu+, Fe3+, etc,) |
|  | PAAG_02046 | sugar transporter | 1.39 | C-compound and carbohydrate transport |
|  | PAAG_08896 | peptide transporter PTR2 | 1.37 | peptide transport |
|  | PAAG_06357 | MFS sugar transporter | 1.34 | C-compound and carbohydrate transport |
|  | PAAG_07383 | vacuolar amino acid transporter 1 | 1.31 | amino acid/amino acid derivatives transport |
|  | PAAG_04976 | inner membrane transport protein yeiJ | 1.25 | nucleotide/nucleoside/nucleobase transport |
|  | PAAG_04859 | MFS multidrug transporter | 1.17 | drug/toxin transport |
|  | PAAG_08200 | mitochondrial 2-oxoglutarate/malate carrier protein | 1.11 | C-compound and carbohydrate transport |
|  | PAAG_00426 | N amino acid transport system protein | 1.09 | amino acid/amino acid derivatives transport |
|  | PAAG_02208 | high-affinity nickel permease | 1.06 | heavy metal ion transport (Cu+, Fe3+, etc,) |
|  | PAAG_07197 | MFS transporter | 1.04 | transport |
| **SIGNAL TRANSDUCTION** | | | | |
|  | PAAG_07416 | kinase domain-containing protein | 2.57 | signal transduction |
|  | PAAG_07232 | kinase domain-containing protein | 1.23 | signal transduction |
|  | PAAG_05483 | ser/Thr protein phosphatase family protein | 1.20 | signal transduction |
| **CELL RESCUE, DEFENSE and VIRULENCE** | | | | |
|  | PAAG_03502 | cytochrome c peroxidase | 3.03 | oxidative stress response |
|  | PAAG_06567 | epoxide hydrolase | 2.72 | detoxification |
|  | PAAG_01454 | catalase | 2.68 | oxidative stress response |
|  | PAAG_02926 | superoxide dismutase | 2.49 | detoxification |
|  | PAAG_02971 | cytosolic Cu/Zn superoxide dismutase | 2.01 | stress response |
|  | PAAG_01842 | beta-lactamase family protein | 1.91 | cell defense and virulence |
|  | PAAG_08410 | acyl-coenzyme A:6-aminopenicillanic-acid-acyltransferase 40 kDa form | 1.87 | cell defense and virulence |
|  | PAAG_01704 | SAM-dependent methyltransferase UbiE/COQ5 family protein | 1.78 | detoxification by modification |
|  | PAAG_04544 | sterigmatocystin biosynthesis monooxygenase StcW | 1.76 | detoxification |
|  | PAAG_01368 | benzoate 4-monooxygenase cytochrome P450 | 1.31 | detoxification involving cytochrome P450 |
|  | PAAG_08277 | nitroreductase family protein | 1.30 | detoxification |
|  | PAAG_05061 | AhpC/TSA family protein | 1.16 | oxidative stress response |
|  | PAAG_00549 | acyl-Coenzyme A dehydrogenase family | 1.09 | superoxide metabolism |
|  | PAAG_02012 | streptomycin biosynthesis protein StrI | 1.08 | cell defense and virulence |
|  | PAAG_02548 | hydroxyacylglutathione hydrolase | 1.03 | glutathione biosynthesis/ antioxidant process |
| **CELL GROWTH/ MORPHOGENESIS** | | | | |
|  | PAAG_00569 | tubulin gamma chain | 1.18 | cytoskeleton |
|  | PAAG_06960 | mannan endo-1,6-alpha-mannosidase DCW1 | 1.16 | fungal and other eukaryotic cell type differentiation |
|  | PAAG_06779 | cell wall glucanase (Utr2) | 1.07 | cell wall |
|  | PAAG_01581 | mannan endo-1,6-alpha-mannosidase DCW1 | 1.01 | cell wall |
| **MISCELLANEOUS** | | | | |
|  | PAAG_02510 | oxidoreductase | 2.17 | secondary metabolism |
|  | PAAG_00906 | acetyltransferase | 1.40 | transferase enzyme |
|  | PAAG_02343 | 1-aminocyclopropane-1-carboxylate oxidase | 1.38 | oxidation-reduction process |
|  | PAAG_03096 | thymine dioxygenase | 1.22 | oxidation-reduction process |
|  | PAAG_04785 | DSBA family oxidoreductase | 1.11 | [cell redox homeostasis](http://www.ebi.ac.uk/QuickGO/GTerm?id=GO:0045454) |
|  | PAAG_02360 | FAD dependent oxidoreductase | 1.02 | oxirreductase |
|  | PAAG_08159 | NADPH dehydrogenase | 1.00 | oxirreductase |
| **UNCLASSIFIED** | | | | |
|  | PAAG_08043 | hypothetical protein | 6.35 | - |
|  | PAAG_03274 | hypothetical protein | 4.40 | - |
|  | PAAG_05657 | conserved hypothetical protein | 4.04 | - |
|  | PAAG_02925 | hypothetical protein | 3.99 | - |
|  | PAAG_08586 | predicted protein | 3.79 | - |
|  | PAAG_05424 | conserved hypothetical protein | 3.75 | - |
|  | PAAG_05709 | predicted protein | 3.74 | - |
|  | PAAG_05252 | predicted protein | 3.74 | - |
|  | PAAG_01384 | predicted protein | 3.46 | - |
|  | PAAG_02490 | predicted protein | 3.40 | - |
|  | PAAG_00853 | predicted protein | 3.40 | - |
|  | PAAG_05158 | proline rich antigen 2 | 3.40 | - |
|  | PAAG_08312 | conserved hypothetical protein | 3.37 | - |
|  | PAAG_06083 | dienelactone hydrolase family protein | 3.34 | - |
|  | PAAG_02513 | conserved hypothetical protein | 3.30 | - |
|  | PAAG_04449 | predicted protein | 3.30 | - |
|  | PAAG_08347 | predicted protein | 3.10 | - |
|  | PAAG_01455 | hypothetical protein | 3.07 | - |
|  | PAAG_00625 | conserved hypothetical protein | 2.94 | - |
|  | PAAG_05156 | predicted protein | 2.88 | - |
|  | PAAG_08266 | predicted protein | 2.88 | - |
|  | PAAG_08056 | predicted protein | 2.87 | - |
|  | PAAG_00172 | hypothetical protein | 2.86 | - |
|  | PAAG_04900 | predicted protein | 2.85 | - |
|  | PAAG_02711 | predicted protein | 2.81 | - |
|  | PAAG_04864 | predicted protein | 2.77 | - |
|  | PAAG_03607 | predicted protein | 2.77 | - |
|  | PAAG_05447 | hypothetical protein | 2.76 | - |
|  | PAAG_01920 | predicted protein | 2.75 | - |
|  | PAAG_07701 | conserved hypothetical protein | 2.74 | - |
|  | PAAG_04491 | predicted protein | 2.71 | - |
|  | PAAG_04935 | hypothetical protein | 2.70 | - |
|  | PAAG_06531 | hypothetical protein | 2.62 | - |
|  | PAAG_01619 | predicted protein | 2.56 | - |
|  | PAAG_04490 | expression library immunization antigen 1 | 2.56 | - |
|  | PAAG_02672 | conserved hypothetical protein | 2.45 | - |
|  | PAAG_04450 | conserved hypothetical protein | 2.43 | - |
|  | PAAG_01996 | conserved hypothetical protein | 2.43 | - |
|  | PAAG_01714 | predicted protein | 2.41 | - |
|  | PAAG_03332 | conserved hypothetical protein | 2.40 | - |
|  | PAAG_08580 | hypothetical protein | 2.39 | - |
|  | PAAG_04451 | conserved hypothetical protein | 2.37 | - |
|  | PAAG_07445 | conserved hypothetical protein | 2.35 | - |
|  | PAAG_06240 | predicted protein | 2.34 | - |
|  | PAAG_01809 | predicted protein | 2.31 | - |
|  | PAAG_03288 | predicted protein | 2.29 | - |
|  | PAAG_03022 | conserved hypothetical protein | 2.29 | - |
|  | PAAG_05650 | conserved hypothetical protein | 2.29 | - |
|  | PAAG_07195 | hypothetical protein | 2.26 | - |
|  | PAAG_01033 | predicted protein | 2.26 | - |
|  | PAAG_06185 | predicted protein | 2.23 | - |
|  | PAAG_08055 | predicted protein | 2.22 | - |
|  | PAAG_03437 | predicted protein | 2.21 | - |
|  | PAAG_05808 | predicted protein | 2.20 | - |
|  | PAAG_03145 | predicted protein | 2.20 | - |
|  | PAAG_08122 | conserved hypothetical protein | 2.19 | - |
|  | PAAG_07545 | conserved hypothetical protein | 2.18 | - |
|  | PAAG_05890 | predicted protein | 2.17 | - |
|  | PAAG_04033 | predicted protein | 2.15 | - |
|  | PAAG_03086 | hypothetical protein | 2.12 | - |
|  | PAAG_04301 | predicted protein | 2.12 | - |
|  | PAAG_00579 | conserved hypothetical protein | 2.08 | - |
|  | PAAG_01636 | hypothetical protein | 2.06 | - |
|  | PAAG_07981 | conserved hypothetical protein | 2.05 | - |
|  | PAAG_02968 | PQ loop repeat protein | 2.04 | - |
|  | PAAG_07907 | predicted protein | 2.04 | - |
|  | PAAG_05599 | predicted protein | 2.04 | - |
|  | PAAG_08850 | predicted protein | 2.02 | - |
|  | PAAG_05264 | conserved hypothetical protein | 2.01 | - |
|  | PAAG_06734 | conserved hypothetical protein | 2.01 | - |
|  | PAAG_00646 | predicted protein | 2.00 | - |
|  | PAAG_02715 | predicted protein | 1.99 | - |
|  | PAAG_02741 | predicted protein | 1.97 | - |
|  | PAAG_00628 | conserved hypothetical protein | 1.96 | - |
|  | PAAG_06566 | conserved hypothetical protein | 1.96 | - |
|  | PAAG_03505 | conserved hypothetical protein | 1.95 | - |
|  | PAAG_02160 | predicted protein | 1.94 | - |
|  | PAAG_08953 | predicted protein | 1.94 | - |
|  | PAAG_00614 | predicted protein | 1.94 | - |
|  | PAAG_03339 | conserved hypothetical protein | 1.93 | - |
|  | PAAG_04359 | predicted protein | 1.92 | - |
|  | PAAG_05792 | predicted protein | 1.92 | - |
|  | PAAG_05394 | predicted protein | 1.92 | - |
|  | PAAG_07402 | conserved hypothetical protein | 1.92 | - |
|  | PAAG_04182 | predicted protein | 1.91 | - |
|  | PAAG_03967 | predicted protein | 1.91 | - |
|  | PAAG_05040 | predicted protein | 1.89 | - |
|  | PAAG_01146 | conserved hypothetical protein | 1.89 | - |
|  | PAAG_00164 | conserved hypothetical protein | 1.88 | - |
|  | PAAG_04080 | predicted protein | 1.88 | - |
|  | PAAG_03928 | predicted protein | 1.87 | - |
|  | PAAG_00898 | conserved hypothetical protein | 1.85 | - |
|  | PAAG_02317 | predicted protein | 1.85 | - |
|  | PAAG_04094 | conserved hypothetical protein | 1.83 | - |
|  | PAAG_00097 | predicted protein | 1.82 | - |
|  | PAAG_00428 | hypothetical protein | 1.82 | - |
|  | PAAG_08899 | conserved hypothetical protein | 1.82 | - |
|  | PAAG_04302 | conserved hypothetical protein | 1.81 | - |
|  | PAAG_00912 | prp 4 CRoW domain-containing protein | 1.81 | - |
|  | PAAG_01361 | DUF895 domain-containing protein | 1.80 | - |
|  | PAAG_08552 | conserved hypothetical protein | 1.79 | - |
|  | PAAG_05801 | predicted protein | 1.79 | - |
|  | PAAG_04795 | conserved hypothetical protein | 1.79 | - |
|  | PAAG_05302 | predicted protein | 1.78 | - |
|  | PAAG_03117 | conserved hypothetical protein | 1.77 | - |
|  | PAAG_05223 | predicted protein | 1.77 | - |
|  | PAAG_03343 | conserved hypothetical protein | 1.76 | - |
|  | PAAG_05027 | predicted protein | 1.76 | - |
|  | PAAG_07922 | predicted protein | 1.76 | - |
|  | PAAG_08109 | conserved hypothetical protein | 1.75 | - |
|  | PAAG_00402 | predicted protein | 1.75 | - |
|  | PAAG_03767 | conserved hypothetical protein | 1.75 | - |
|  | PAAG_04513 | predicted protein | 1.75 | - |
|  | PAAG_01837 | hypothetical protein | 1.75 | - |
|  | PAAG_00082 | predicted protein | 1.74 | - |
|  | PAAG_00135 | conserved hypothetical protein | 1.74 | - |
|  | PAAG_01667 | predicted protein | 1.73 | - |
|  | PAAG_02318 | predicted protein | 1.73 | - |
|  | PAAG_04307 | predicted protein | 1.73 | - |
|  | PAAG_00584 | conserved hypothetical protein | 1.73 | - |
|  | PAAG_03119 | predicted protein | 1.72 | - |
|  | PAAG_01638 | predicted protein | 1.72 | - |
|  | PAAG_02216 | predicted protein | 1.71 | - |
|  | PAAG_02514 | predicted protein | 1.71 | - |
|  | PAAG_04404 | predicted protein | 1.71 | - |
|  | PAAG_05567 | conserved hypothetical protein | 1.70 | - |
|  | PAAG_01429 | predicted protein | 1.69 | - |
|  | PAAG_01635 | predicted protein | 1.69 | - |
|  | PAAG_03087 | predicted protein | 1.68 | - |
|  | PAAG_03966 | hypothetical protein | 1.67 | - |
|  | PAAG_02988 | conserved hypothetical protein | 1.67 | - |
|  | PAAG_02670 | predicted protein | 1.67 | - |
|  | PAAG_02565 | hypothetical protein | 1.67 | - |
|  | PAAG_05343 | conserved hypothetical protein | 1.67 | - |
|  | PAAG_06375 | hypothetical protein | 1.67 | - |
|  | PAAG_06374 | conserved hypothetical protein | 1.66 | - |
|  | PAAG_00278 | conserved hypothetical protein | 1.65 | - |
|  | PAAG_06241 | conserved hypothetical protein | 1.65 | - |
|  | PAAG_05479 | hypothetical protein | 1.64 | - |
|  | PAAG_01694 | predicted protein | 1.64 | - |
|  | PAAG_01836 | predicted protein | 1.64 | - |
|  | PAAG_08406 | predicted protein | 1.64 | - |
|  | PAAG_04782 | predicted protein | 1.63 | - |
|  | PAAG_05433 | predicted protein | 1.63 | - |
|  | PAAG_04830 | predicted protein | 1.62 | - |
|  | PAAG_05478 | conserved hypothetical protein | 1.61 | - |
|  | PAAG_06646 | conserved hypothetical protein | 1.61 | - |
|  | PAAG_06346 | predicted protein | 1.61 | - |
|  | PAAG_02315 | predicted protein | 1.59 | - |
|  | PAAG_07848 | conserved hypothetical protein | 1.58 | - |
|  | PAAG_04153 | conserved hypothetical protein | 1.57 | - |
|  | PAAG_05600 | hypothetical protein | 1.57 | - |
|  | PAAG_05334 | hypothetical protein | 1.57 | - |
|  | PAAG_00839 | hypothetical protein | 1.57 | - |
|  | PAAG_00854 | conserved hypothetical protein | 1.57 | - |
|  | PAAG_07331 | hypothetical protein | 1.56 | - |
|  | PAAG_03151 | conserved hypothetical protein | 1.55 | - |
|  | PAAG_07783 | hypothetical protein | 1.55 | - |
|  | PAAG_08161 | predicted protein | 1.55 | - |
|  | PAAG_05656 | conserved hypothetical protein | 1.53 | - |
|  | PAAG_00687 | hypothetical protein | 1.53 | - |
|  | PAAG_01642 | predicted protein | 1.53 | - |
|  | PAAG_01332 | predicted protein | 1.52 | - |
|  | PAAG_02918 | predicted protein | 1.52 | - |
|  | PAAG_00955 | conserved hypothetical protein | 1.52 | - |
|  | PAAG_08393 | predicted protein | 1.52 | - |
|  | PAAG_02491 | conserved hypothetical protein | 1.52 | - |
|  | PAAG_02710 | conserved hypothetical protein | 1.51 | - |
|  | PAAG_05636 | hypothetical protein | 1.51 | - |
|  | PAAG_05445 | predicted protein | 1.51 | - |
|  | PAAG_02156 | conserved hypothetical protein | 1.50 | - |
|  | PAAG_01007 | predicted protein | 1.49 | - |
|  | PAAG_01471 | predicted protein | 1.49 | - |
|  | PAAG_07361 | conserved hypothetical protein | 1.49 | - |
|  | PAAG_04047 | conserved hypothetical protein | 1.49 | - |
|  | PAAG_06182 | predicted protein | 1.48 | - |
|  | PAAG_08877 | hypothetical protein | 1.48 | - |
|  | PAAG_06029 | predicted protein | 1.48 | - |
|  | PAAG_04500 | predicted protein | 1.48 | - |
|  | PAAG_06285 | predicted protein | 1.47 | - |
|  | PAAG_05543 | conserved hypothetical protein | 1.47 | - |
|  | PAAG_06820 | hypothetical protein | 1.46 | - |
|  | PAAG_02118 | conserved hypothetical protein | 1.46 | - |
|  | PAAG_07081 | conserved hypothetical protein | 1.46 | - |
|  | PAAG_05886 | conserved hypothetical protein | 1.46 | - |
|  | PAAG_01182 | hypothetical protein | 1.46 | - |
|  | PAAG_01441 | conserved hypothetical protein | 1.46 | - |
|  | PAAG_07332 | conserved hypothetical protein | 1.45 | - |
|  | PAAG_06119 | conserved hypothetical protein | 1.45 | - |
|  | PAAG_02719 | conserved hypothetical protein | 1.45 | - |
|  | PAAG_01098 | conserved hypothetical protein | 1.45 | - |
|  | PAAG_02952 | predicted protein | 1.44 | - |
|  | PAAG_04035 | conserved hypothetical protein | 1.44 | - |
|  | PAAG_03144 | predicted protein | 1.44 | - |
|  | PAAG_05624 | conserved hypothetical protein | 1.44 | - |
|  | PAAG_08502 | hypothetical protein | 1.43 | - |
|  | PAAG_08878 | conserved hypothetical protein | 1.43 | - |
|  | PAAG_01422 | conserved hypothetical protein | 1.43 | - |
|  | PAAG_05342 | conserved hypothetical protein | 1.43 | - |
|  | PAAG_05011 | predicted protein | 1.43 | - |
|  | PAAG_00407 | predicted protein | 1.42 | - |
|  | PAAG_02373 | predicted protein | 1.42 | - |
|  | PAAG_01469 | tachykinin family protein | 1.42 | - |
|  | PAAG_00942 | conserved hypothetical protein | 1.42 | - |
|  | PAAG_06624 | predicted protein | 1.41 | - |
|  | PAAG_00503 | HAD-superfamily hydrolase | 1.41 | - |
|  | PAAG_04943 | conserved hypothetical protein | 1.41 | - |
|  | PAAG_03588 | conserved hypothetical protein | 1.40 | - |
|  | PAAG_09023 | conserved hypothetical protein | 1.40 | - |
|  | PAAG_08588 | hypothetical protein | 1.40 | - |
|  | PAAG_06413 | predicted protein | 1.39 | - |
|  | PAAG_01121 | predicted protein | 1.39 | - |
|  | PAAG_06184 | predicted protein | 1.39 | - |
|  | PAAG_04492 | conserved hypothetical protein | 1.39 | - |
|  | PAAG_08144 | conserved hypothetical protein | 1.39 | - |
|  | PAAG_05472 | predicted protein | 1.39 | - |
|  | PAAG_02464 | predicted protein | 1.38 | - |
|  | PAAG_00095 | predicted protein | 1.38 | - |
|  | PAAG_04325 | hypothetical protein | 1.38 | - |
|  | PAAG_05799 | conserved hypothetical protein | 1.38 | - |
|  | PAAG_03403 | predicted protein | 1.38 | - |
|  | PAAG_07196 | hypothetical protein | 1.37 | - |
|  | PAAG_07578 | predicted protein | 1.36 | - |
|  | PAAG_02400 | predicted protein | 1.36 | - |
|  | PAAG_05471 | predicted protein | 1.36 | - |
|  | PAAG_03848 | predicted protein | 1.36 | - |
|  | PAAG_01640 | predicted protein | 1.36 | - |
|  | PAAG_07755 | predicted protein | 1.35 | - |
|  | PAAG_04659 | predicted protein | 1.35 | - |
|  | PAAG_02986 | hypothetical protein | 1.35 | - |
|  | PAAG_00776 | hypothetical protein | 1.35 | - |
|  | PAAG_01827 | integral membrane protein | 1.34 | - |
|  | PAAG_06801 | predicted protein | 1.34 | - |
|  | PAAG_00439 | conserved hypothetical protein | 1.34 | - |
|  | PAAG_04915 | predicted protein | 1.34 | - |
|  | PAAG_03273 | conserved hypothetical protein | 1.34 | - |
|  | PAAG_00343 | hypothetical protein | 1.34 | - |
|  | PAAG_01997 | predicted protein | 1.33 | - |
|  | PAAG_00171 | conserved hypothetical protein | 1.33 | - |
|  | PAAG_02193 | conserved hypothetical protein | 1.33 | - |
|  | PAAG_08046 | conserved hypothetical protein | 1.33 | - |
|  | PAAG_03580 | hypothetical protein | 1.32 | - |
|  | PAAG_03341 | hypothetical protein | 1.31 | - |
|  | PAAG_07971 | conserved hypothetical protein | 1.31 | - |
|  | PAAG_07093 | hypothetical protein | 1.31 | - |
|  | PAAG_02167 | predicted protein | 1.31 | - |
|  | PAAG_01406 | conserved hypothetical protein | 1.30 | - |
|  | PAAG_02247 | conserved hypothetical protein | 1.30 | - |
|  | PAAG_02174 | hypothetical protein | 1.30 | - |
|  | PAAG_06553 | conserved hypothetical protein | 1.30 | - |
|  | PAAG_03033 | conserved hypothetical protein | 1.30 | - |
|  | PAAG_03498 | conserved hypothetical protein | 1.30 | - |
|  | PAAG_00444 | hypothetical protein | 1.30 | - |
|  | PAAG_04968 | conserved hypothetical protein | 1.30 | - |
|  | PAAG_01331 | predicted protein | 1.30 | - |
|  | PAAG_02766 | conserved hypothetical protein | 1.30 | - |
|  | PAAG_02087 | conserved hypothetical protein | 1.29 | - |
|  | PAAG_03085 | predicted protein | 1.29 | - |
|  | PAAG_06614 | predicted protein | 1.29 | - |
|  | PAAG_01634 | conserved hypothetical protein | 1.29 | - |
|  | PAAG_05819 | predicted protein | 1.29 | - |
|  | PAAG_01922 | conserved hypothetical protein | 1.29 | - |
|  | PAAG_04724 | conserved hypothetical protein | 1.29 | - |
|  | PAAG_06838 | conserved hypothetical protein | 1.29 | - |
|  | PAAG_05208 | predicted protein | 1.29 | - |
|  | PAAG_00418 | hypothetical protein | 1.28 | - |
|  | PAAG_01284 | predicted protein | 1.28 | - |
|  | PAAG_05274 | predicted protein | 1.28 | - |
|  | PAAG_05568 | conserved hypothetical protein | 1.28 | - |
|  | PAAG_05947 | conserved hypothetical protein | 1.28 | - |
|  | PAAG_06444 | conserved hypothetical protein | 1.28 | - |
|  | PAAG_08054 | predicted protein | 1.28 | - |
|  | PAAG_01652 | predicted protein | 1.28 | - |
|  | PAAG_08106 | conserved hypothetical protein | 1.28 | - |
|  | PAAG_05627 | predicted protein | 1.27 | - |
|  | PAAG_04034 | hypothetical protein | 1.27 | - |
|  | PAAG_05209 | conserved hypothetical protein | 1.27 | - |
|  | PAAG_00332 | conserved hypothetical protein | 1.27 | - |
|  | PAAG_03589 | conserved hypothetical protein | 1.26 | - |
|  | PAAG_07329 | predicted protein | 1.26 | - |
|  | PAAG_06447 | predicted protein | 1.26 | - |
|  | PAAG_04702 | predicted protein | 1.26 | - |
|  | PAAG_04431 | predicted protein | 1.25 | - |
|  | PAAG_07446 | conserved hypothetical protein | 1.25 | - |
|  | PAAG_02187 | hypothetical protein | 1.24 | - |
|  | PAAG_01254 | predicted protein | 1.24 | - |
|  | PAAG_00918 | hypothetical protein | 1.24 | - |
|  | PAAG_07800 | hypothetical protein | 1.24 | - |
|  | PAAG_08963 | hypothetical protein | 1.24 | - |
|  | PAAG_01402 | hypothetical protein | 1.24 | - |
|  | PAAG_00087 | conserved hypothetical protein | 1.24 | - |
|  | PAAG_05470 | predicted protein | 1.24 | - |
|  | PAAG_06640 | predicted protein | 1.24 | - |
|  | PAAG_00210 | conserved hypothetical protein | 1.23 | - |
|  | PAAG_04337 | conserved hypothetical protein | 1.23 | - |
|  | PAAG_06187 | predicted protein | 1.23 | - |
|  | PAAG_00551 | hypothetical protein | 1.23 | - |
|  | PAAG_01564 | conserved hypothetical protein | 1.23 | - |
|  | PAAG_08870 | conserved hypothetical protein | 1.23 | - |
|  | PAAG_02243 | predicted protein | 1.22 | - |
|  | PAAG_07025 | predicted protein | 1.22 | - |
|  | PAAG_08780 | predicted protein | 1.22 | - |
|  | PAAG_07363 | predicted protein | 1.22 | - |
|  | PAAG_04384 | conserved hypothetical protein | 1.22 | - |
|  | PAAG_00913 | conserved hypothetical protein | 1.22 | - |
|  | PAAG_06131 | conserved hypothetical protein | 1.22 | - |
|  | PAAG_00033 | hypothetical protein | 1.22 | - |
|  | PAAG_04944 | predicted protein | 1.22 | - |
|  | PAAG_05492 | conserved hypothetical protein | 1.21 | - |
|  | PAAG_08041 | predicted protein | 1.21 | - |
|  | PAAG_05188 | hypothetical protein | 1.21 | - |
|  | PAAG_01253 | predicted protein | 1.21 | - |
|  | PAAG_08354 | hypothetical protein | 1.21 | - |
|  | PAAG_05856 | conserved hypothetical protein | 1.21 | - |
|  | PAAG_04350 | conserved hypothetical protein | 1.21 | - |
|  | PAAG_04530 | conserved hypothetical protein | 1.21 | - |
|  | PAAG_08212 | predicted protein | 1.21 | - |
|  | PAAG_01008 | conserved hypothetical protein | 1.21 | - |
|  | PAAG_06837 | molybdopterin synthase small subunit CnxG | 1.21 | - |
|  | PAAG_00835 | conserved hypothetical protein | 1.20 | - |
|  | PAAG_02564 | predicted protein | 1.20 | - |
|  | PAAG_06708 | conserved hypothetical protein | 1.20 | - |
|  | PAAG_08523 | conserved hypothetical protein | 1.20 | - |
|  | PAAG_05625 | predicted protein | 1.20 | - |
|  | PAAG_06337 | predicted protein | 1.20 | - |
|  | PAAG_00615 | conserved hypothetical protein | 1.20 | - |
|  | PAAG_04019 | predicted protein | 1.19 | - |
|  | PAAG_07875 | conserved hypothetical protein | 1.19 | - |
|  | PAAG_00539 | predicted protein | 1.19 | - |
|  | PAAG_02144 | conserved hypothetical protein | 1.19 | - |
|  | PAAG_08018 | predicted protein | 1.19 | - |
|  | PAAG_02985 | hypothetical protein | 1.19 | - |
|  | PAAG_07817 | predicted protein | 1.19 | - |
|  | PAAG_00297 | conserved hypothetical protein | 1.19 | - |
|  | PAAG_02305 | conserved hypothetical protein | 1.19 | - |
|  | PAAG_07974 | predicted protein | 1.19 | - |
|  | PAAG_05097 | hypothetical protein | 1.19 | - |
|  | PAAG_00823 | conserved hypothetical protein | 1.18 | - |
|  | PAAG_00223 | predicted protein | 1.18 | - |
|  | PAAG_00624 | conserved hypothetical protein | 1.18 | - |
|  | PAAG_00840 | predicted protein | 1.18 | - |
|  | PAAG_01999 | predicted protein | 1.18 | - |
|  | PAAG_00837 | predicted protein | 1.18 | - |
|  | PAAG_01637 | conserved hypothetical protein | 1.17 | - |
|  | PAAG_05779 | hypothetical protein | 1.17 | - |
|  | PAAG_07718 | predicted protein | 1.17 | - |
|  | PAAG_04861 | predicted protein | 1.16 | - |
|  | PAAG_09050 | conserved hypothetical protein | 1.16 | - |
|  | PAAG_03082 | predicted protein | 1.16 | - |
|  | PAAG_08906 | conserved hypothetical protein | 1.16 | - |
|  | PAAG_04011 | conserved hypothetical protein | 1.15 | - |
|  | PAAG_05514 | conserved hypothetical protein | 1.15 | - |
|  | PAAG_05320 | predicted protein | 1.15 | - |
|  | PAAG_07681 | hypothetical protein | 1.15 | - |
|  | PAAG_06186 | predicted protein | 1.15 | - |
|  | PAAG_02914 | conserved hypothetical protein | 1.14 | - |
|  | PAAG_05655 | hypothetical protein | 1.14 | - |
|  | PAAG_01448 | conserved hypothetical protein | 1.14 | - |
|  | PAAG_03862 | predicted protein | 1.14 | - |
|  | PAAG_05387 | conserved hypothetical protein | 1.14 | - |
|  | PAAG_01430 | integral membrane protein | 1.14 | - |
|  | PAAG_03475 | conserved hypothetical protein | 1.14 | - |
|  | PAAG_05431 | conserved hypothetical protein | 1.13 | - |
|  | PAAG_06717 | conserved hypothetical protein | 1.13 | - |
|  | PAAG_09119 | predicted protein | 1.13 | - |
|  | PAAG_05341 | conserved hypothetical protein | 1.13 | - |
|  | PAAG_01153 | hypothetical protein | 1.13 | - |
|  | PAAG_02623 | predicted protein | 1.13 | - |
|  | PAAG_00973 | predicted protein | 1.13 | - |
|  | PAAG_00931 | hypothetical protein | 1.13 | - |
|  | PAAG_08844 | conserved hypothetical protein | 1.12 | - |
|  | PAAG_04714 | predicted protein | 1.12 | - |
|  | PAAG_04057 | conserved hypothetical protein | 1.12 | - |
|  | PAAG_04732 | conserved hypothetical protein | 1.12 | - |
|  | PAAG_02692 | cupin domain-containing protein | 1.12 | - |
|  | PAAG_03140 | OPA3 domain-containing protein | 1.12 | - |
|  | PAAG_02807 | conserved hypothetical protein | 1.12 | - |
|  | PAAG_02550 | predicted protein | 1.11 | - |
|  | PAAG_00784 | predicted protein | 1.11 | - |
|  | PAAG_08142 | conserved hypothetical protein | 1.11 | - |
|  | PAAG_08962 | predicted protein | 1.10 | - |
|  | PAAG_00137 | hypothetical protein | 1.10 | - |
|  | PAAG_05513 | hypothetical protein | 1.10 | - |
|  | PAAG_09011 | conserved hypothetical protein | 1.10 | - |
|  | PAAG_01544 | conserved hypothetical protein | 1.10 | - |
|  | PAAG_00636 | conserved hypothetical protein | 1.10 | - |
|  | PAAG_05248 | conserved hypothetical protein | 1.10 | - |
|  | PAAG_06193 | conserved hypothetical protein | 1.10 | - |
|  | PAAG_00336 | predicted protein | 1.10 | - |
|  | PAAG_04748 | conserved hypothetical protein | 1.09 | - |
|  | PAAG_03712 | predicted protein | 1.09 | - |
|  | PAAG_04066 | predicted protein | 1.09 | - |
|  | PAAG_01072 | conserved hypothetical protein | 1.09 | - |
|  | PAAG_07870 | predicted protein | 1.09 | - |
|  | PAAG_01079 | hypothetical protein | 1.09 | - |
|  | PAAG_01968 | conserved hypothetical protein | 1.09 | - |
|  | PAAG_06836 | predicted protein | 1.08 | - |
|  | PAAG_07401 | ammecr1 family protein | 1.08 | - |
|  | PAAG_04955 | predicted protein | 1.08 | - |
|  | PAAG_06056 | predicted protein | 1.08 | - |
|  | PAAG_08050 | hypothetical protein | 1.08 | - |
|  | PAAG_06076 | predicted protein | 1.08 | - |
|  | PAAG_02967 | predicted protein | 1.08 | - |
|  | PAAG_08408 | conserved hypothetical protein | 1.08 | - |
|  | PAAG_00055 | predicted protein | 1.08 | - |
|  | PAAG_08068 | conserved hypothetical protein | 1.07 | - |
|  | PAAG_08505 | conserved hypothetical protein | 1.07 | - |
|  | PAAG_01275 | conserved hypothetical protein | 1.07 | - |
|  | PAAG_02780 | predicted protein | 1.07 | - |
|  | PAAG_03630 | predicted protein | 1.07 | - |
|  | PAAG_06492 | predicted protein | 1.07 | - |
|  | PAAG_02389 | conserved hypothetical protein | 1.06 | - |
|  | PAAG_03799 | predicted protein | 1.06 | - |
|  | PAAG_05949 | hypothetical protein | 1.06 | - |
|  | PAAG_04156 | conserved hypothetical protein | 1.06 | - |
|  | PAAG_06181 | predicted protein | 1.06 | - |
|  | PAAG_07745 | conserved hypothetical protein | 1.06 | - |
|  | PAAG_08241 | predicted protein | 1.06 | - |
|  | PAAG_04413 | predicted protein | 1.06 | - |
|  | PAAG_08554 | predicted protein | 1.06 | - |
|  | PAAG_05220 | predicted protein | 1.05 | - |
|  | PAAG_03337 | predicted protein | 1.05 | - |
|  | PAAG_05651 | hypothetical protein | 1.05 | - |
|  | PAAG_01738 | predicted protein | 1.05 | - |
|  | PAAG_03356 | predicted protein | 1.05 | - |
|  | PAAG_04683 | predicted protein | 1.05 | - |
|  | PAAG_00300 | predicted protein | 1.05 | - |
|  | PAAG_06930 | predicted protein | 1.05 | - |
|  | PAAG_02579 | conserved hypothetical protein | 1.05 | - |
|  | PAAG_08190 | predicted protein | 1.05 | - |
|  | PAAG_03272 | predicted protein | 1.05 | - |
|  | PAAG_05219 | predicted protein | 1.04 | - |
|  | PAAG_01688 | predicted protein | 1.04 | - |
|  | PAAG_00522 | conserved hypothetical protein | 1.04 | - |
|  | PAAG_05595 | predicted protein | 1.04 | - |
|  | PAAG_08271 | predicted protein | 1.03 | - |
|  | PAAG_05035 | predicted protein | 1.03 | - |
|  | PAAG_02334 | conserved hypothetical protein | 1.03 | - |
|  | PAAG_05503 | predicted protein | 1.03 | - |
|  | PAAG_04617 | hypothetical protein | 1.03 | - |
|  | PAAG_00917 | conserved hypothetical protein | 1.03 | - |
|  | PAAG_02748 | conserved hypothetical protein | 1.03 | - |
|  | PAAG_06802 | conserved hypothetical protein | 1.03 | - |
|  | PAAG_05955 | predicted protein | 1.03 | - |
|  | PAAG_07193 | predicted protein | 1.02 | - |
|  | PAAG_07035 | conserved hypothetical protein | 1.02 | - |
|  | PAAG_05870 | predicted protein | 1.02 | - |
|  | PAAG_03995 | hypothetical protein | 1.02 | - |
|  | PAAG_09084 | predicted protein | 1.02 | - |
|  | PAAG_07199 | hypothetical protein | 1.02 | - |
|  | PAAG_02849 | predicted protein | 1.02 | - |
|  | PAAG_01687 | hypothetical protein | 1.01 | - |
|  | PAAG_08294 | conserved hypothetical protein | 1.01 | - |
|  | PAAG_07599 | conserved hypothetical protein | 1.01 | - |
|  | PAAG_00421 | predicted protein | 1.01 | - |
|  | PAAG_03769 | predicted protein | 1.01 | - |
|  | PAAG_02559 | Apc13 domain-containing protein | 1.01 | - |
|  | PAAG_05344 | conserved hypothetical protein | 1.00 | - |
|  | PAAG_05152 | hypothetical protein | 1.00 | - |
|  | PAAG_05056 | conserved hypothetical protein | 1.00 | - |
|  | PAAG_05695 | conserved hypothetical protein | 1.00 | - |
|  | PAAG_04733 | hypothetical protein | 1.00 | - |
|  | PAAG_05673 | predicted protein | 1.00 | - |
|  | PAAG_02661 | conserved hypothetical protein | 1.00 | - |
|  | PAAG_05552 | DUF614 domain-containing protein | 1.00 | - |
|  | PAAG_05839 | conserved hypothetical protein | 1.00 | - |
|  | PAAG_00887 | conserved hypothetical protein | 1.00 | - |
|  | PAAG_07758 | predicted protein | 1.00 | - |
|  | PAAG_07418 | conserved hypothetical protein | 1.00 | - |

^a^ Identification of differentially regulated transcripts from *Paracoccidioides* genome database (<http://www.broadinstitute.org/annotation/genome/paracoccidioides_brasiliensis/MultiHome.html>).

^b^ Transcripts annotation from *Paracoccidioides* genome database or by homology from NCBI database (<http://www.ncbi.nlm.nih.gov/>);

^c^ Transcripts expression profiles in log2_fold change obtained from fold change selection method for differentially expressed transcripts using a Fisher exact test with a p-value of 0.001.

^d^ Biological process of differentially expressed transcripts from MIPS

(<http://pedant.helmholtz-muenchen.de/pedant3htmlview/pedant3view?Method=analysis&Db=p3_r48325_Par_brasi_Pb01>).
