# Supplementary material for: Transcriptional and Proteomic Responses to Carbon Starvation in Paracoccidioides
Source: PLoS Negl Trop Dis. 2014 May 8;8(5):e2855. doi: 10.1371/journal.pntd.0002855 (PMC4014450; doi:10.1371/journal.pntd.0002855)
Supplement: Table S4 — Down-regulated transcripts of Paracoccidioides ( Pb 01) yeast cells under carbon starvation detected by RNAseq analysis. (DOC) [file pntd.0002855.s015.doc]

**Table S4. Down-regulated transcripts of *Paracoccidioides* (*Pb*01) yeast cells under carbon starvation detected by RNAseq analysis.**

|  | **ID^a^** | **Annotation^b^** | **Fold change (log2)^c^** | **Biological process^d^** |
| --- | --- | --- | --- | --- |
| **METABOLISM** | | | | |
| **Amino acid metabolism** | | | | |
|  | PAAG_07999 | glutamate synthase | -3.69 | glutamate group biosynthesis |
|  | PAAG_03903 | sulfite reductase subunit beta | -3.07 | cysteine biosynthesis |
|  | PAAG_05932 | phosphoadenosine phosphosulfate reductase | -2.84 | cysteine biosynthesis |
|  | PAAG_05929 | sulfate adenylyltransferase | -2.74 | cysteine and methionine biosynthesis |
|  | PAAG_04052 | homoserine O-acetyltransferase | -2.38 | cysteine biosynthesis |
|  | PAAG_07102 | pentafunctional AROM polypeptide | -1.83 | aromatic aminoacid biosynthesis |
|  | PAAG_05328 | 3-isopropylmalate dehydrogenase A | -1.54 | leucine biosynthesis |
|  | PAAG_07998 | glutamate synthase small chain | -1.54 | glutamate group biosynthesis |
|  | PAAG_02901 | S-adenosylmethionine synthetase | -1.38 | methionine degradation |
|  | PAAG_07813 | cysteine synthase | -1.34 | cysteine biosynthesis |
|  | PAAG_04925 | homoserine O-acetyltransferase | -1.18 | methionine biosynthesis |
|  | PAAG_04835 | NADP-dependent L-serine/L-allo-threonine dehydrogenase ydfG | -1.17 | threonine and serine degradation |
|  | PAAG_06289 | tryptophan synthase | -1.07 | tryptophan biosynthesis |
|  | PAAG_01882 | acetolactate synthase catalytic subunit | -1.06 | isoleucine biosynthesis |
|  | PAAG_03296 | 3-isopropylmalate dehydratase | -1.02 | leucine biosynthesis |
| **Nitrogen and sulfur metabolism** | | | | |
|  | PAAG_01570 | choline-sulfatase | -4.90 | nitrogen, sulfur and selenium metabolism |
|  | PAAG_00795 | sulfate permease | -4.81 | nitrogen, sulfur and selenium metabolism |
|  | PAAG_00157 | sulfur metabolite repression control protein | -3.07 | regulation of sulphur metabolism |
|  | PAAG_08148 | sulfite reductase flavoprotein component | -2.62 | nitrogen, sulfur and selenium metabolism |
|  | PAAG_03845 | succinate-semialdehyde dehydrogenase | -1.27 | nitrogen metabolism |
|  | PAAG_06658 | acetamidase | -1.01 | nitrogen, sulfur and selenium metabolism |
| **C-compound and carbohydrate metabolism** | | | | |
|  | PAAG_05686 | glucan 1,3-beta-glucosidase | -1.95 | polysaccharide metabolism |
|  | PAAG_02998 | FAD binding domain-containing protein | -1.69 | C-compound and carbohydrate metabolism |
|  | PAAG_00545 | glycogen phosphorylase | -1.68 | C-compound and carbohydrate metabolism |
|  | PAAG_02769 | pyruvate dehydrogenase protein X component | -1.63 | C-compound and carbohydrate metabolism |
|  | PAAG_02806 | aldehyde reductase | -1.55 | C-compound and carbohydrate metabolism |
|  | PAAG_01405 | inositol oxygenase | -1.46 | Sugar, glucoside, polyol and carboxylate metabolism |
|  | PAAG_06535 | endochitinase | -1.32 | polysaccharide metabolism |
|  | PAAG_08326 | polysaccharide synthase Cps1 | -1.19 | polysaccharide metabolism |
|  | PAAG_00505 | morphine 6-dehydrogenase | -1.13 | C-compound and carbohydrate metabolism |
|  | PAAG_00091 | beta-glucan synthesis-associated protein KRE6 | -1.08 | Sugar, glucoside, polyol and carboxylate catabolism |
|  | PAAG_03142 | 2-hydroxyacid dehydrogenase | -1.07 | C-compound and carbohydrate metabolism |
|  | PAAG_08128 | sugar isomerase | -1.06 | C-compound and carbohydrate metabolism |
|  | PAAG_04857 | beta-glucosidase | -1.00 | polysaccharide metabolism |
| **Lipid, fatty acid and isoprenoid metabolism** | | | | |
|  | PAAG_06877 | triacylglycerol lipase | -1.65 | lipid, fatty acid and isoprenoid metabolism |
|  | PAAG_03722 | acyl-CoA desaturase | -1.45 | lipid, fatty acid and isoprenoid metabolism_biosynthesis |
|  | PAAG_07665 | 4-coumarate-CoA ligase | -1.35 | lipid, fatty acid and isoprenoid metabolism |
|  | PAAG_05665 | lipase | -1.27 | lipid, fatty acid and isoprenoid metabolism |
|  | PAAG_03104 | alkaline phytoceramidase | -1.24 | lipid, fatty acid and isoprenoid metabolism |
|  | PAAG_02292 | phosphomevalonate kinase | -1.24 | lipid, fatty acid and isoprenoid metabolism |
|  | PAAG_00310 | glycerol-3-phosphate dehydrogenase | -1.15 | lipid metabolism_glycerol accumulation |
|  | PAAG_00825 | phosphatidylinositol 4-kinase STT4 | -1.14 | lipid, fatty acid and isoprenoid metabolism |
|  | PAAG_02604 | L-tyrosine decarboxylase | -1.14 | lipid, fatty acid and isoprenoid metabolism |
|  | PAAG_07425 | UDP-glucose:sterol glycosyltransferase | -1.07 | glycolipid metabolism |
|  | PAAG_07722 | 1-phosphatidylinositol-4,5-bisphosphate phosphodiesterase | -1.05 | lipid, fatty acid and isoprenoid metabolism |
|  | PAAG_06230 | NADPH:adrenodoxin oxidoreductase | -1.04 | lipid, fatty acid and isoprenoid metabolism |
|  | PAAG_09079 | sphingolipid C4-hydroxylase SUR2 | -1.00 | lipid, fatty acid and isoprenoid metabolism |
| **Purin nucleotide, nucleoside and nucleobase**  **metabolism** | | | | |
|  | PAAG_06906 | adenine phosphoribosyltransferase | -1.24 | purin nucleotide/nucleoside/nucleobase metabolism |
|  | PAAG_07682 | ribonucleoside-diphosphate reductase large chain | -1.09 | deoxyribonucleotide metabolism |
|  | PAAG_01569 | guanine deaminase | -1.06 | purin nucleotide/nucleoside/nucleobase metabolism |
| **Secondary metabolism** | | | | |
|  | PAAG_05942 | uroporphyrinogen-III C-methyltransferase | -2.79 | biosynthesis of vitamins, cofactors, and prosthetic groups |
|  | PAAG_09037 | 3-dehydroshikimate dehydratase | -2.79 | metabolism of derivatives of dehydroquinic acid, shikimic acid and chorismic acid |
| **ENERGY** | | | | |
| **Glycolysis and gluconeogenesis** | | | | |
|  | PAAG_00771 | enolase | -1.19 | glycolysis and gluconeogenesis |
| **Electron transport and membrane-associated**  **energy conservation** | | | | |
|  | PAAG_02960 | external NADH-ubiquinone oxidoreductase | -1.13 | aerobic respiration |
|  | PAAG_00953 | NADH-cytochrome b5 reductase | -1.06 | electron transport/ respiration |
| **CELL CYCLE and DNA PROCESSING** | | | | |
|  | PAAG_02452 | DNA ligase | -1.68 | DNA recombination and DNA repair |
|  | PAAG_07398 | DNA polymerase epsilon catalytic subunit A | -1.57 | DNA synthesis and replication |
|  | PAAG_08713 | DNA mismatch repair protein msh6 | -1.47 | DNA repair |
|  | PAAG_00153 | chromosome segregation protein sudA | -1.44 | cell cycle |
|  | PAAG_06641 | cell division control protein Cdc25 | -1.43 | mitotic cell cycle and cell cycle control |
|  | PAAG_06486 | DNA polymerase alpha catalytic subunit | -1.36 | DNA synthesis and replication |
|  | PAAG_07170 | eukaryotic translation initiation factor 3 135 kDa subunit | -1.30 | cytokinesis (cell division) /septum formation and hydrolysis |
|  | PAAG_00670 | cohesin complex subunit | -1.27 | mitotic cell cycle |
|  | PAAG_07058 | DNA repair protein rad50 | -1.26 | DNA recombination and DNA repair |
|  | PAAG_03835 | septin | -1.26 | cytokinesis (cell division) /septum formation and hydrolysis |
|  | PAAG_06571 | sister chromatid cohesion protein pds5 | -1.24 | mitotic cell cycle |
|  | PAAG_07314 | MCM3-associated protein | -1.24 | DNA synthesis and replication |
|  | PAAG_04987 | helicase family member | -1.23 | DNA topology |
|  | PAAG_06722 | DNA replication licensing factor mcm7 | -1.20 | DNA synthesis and replication |
|  | PAAG_02909 | origin recognition complex subunit 1 | -1.19 | DNA synthesis and replication |
|  | PAAG_06271 | cyclin-dependent protein kinase PHO85 | -1.19 | mitotic cell cycle and cell cycle control |
|  | PAAG_03706 | nucleoporin Nup157/170 | -1.19 | chromosome segregation/division |
|  | PAAG_03993 | DNA replication licensing factor MCM2 | -1.18 | DNA synthesis and replication |
|  | PAAG_03866 | mitotic cohesin complex | -1.17 | mitotic cell cycle |
|  | PAAG_00984 | DNA polymerase epsilon subunit B | -1.15 | DNA synthesis and replication |
|  | PAAG_06688 | DNA repair protein RAD16 | -1.15 | DNA repair |
|  | PAAG_02533 | nuclear pore complex subunit Nup192 | -1.10 | cell cycle |
|  | PAAG_00605 | DNA mismatch repair protein pms1 | -1.10 | DNA repair |
|  | PAAG_08499 | pre-mRNA-splicing factor ATP-dependent RNA helicase PRP16 | -1.06 | DNA processing |
|  | PAAG_07993 | minichromosome loss protein | -1.05 | mitotic cell cycle and cell cycle control |
|  | PAAG_06601 | DNA polymerase delta catalytic subunit | -1.04 | DNA synthesis and replication |
|  | PAAG_02310 | DNA replication licensing factor MCM7 component | -1.04 | DNA synthesis and replication |
|  | PAAG_05247 | double-strand break repair protein mus-23 | -1.04 | DNA recombination and DNA repair |
|  | PAAG_07894 | DNA mismatch repair protein msh-2 | -1.03 | DNA repair |
|  | PAAG_07638 | histone chaperone Rttp106-like | -1.02 | DNA recombination |
|  | PAAG_05598 | MYB family conidiophore development protein FlbD | -1.02 | G1/S transition of mitotic cell cycle |
|  | PAAG_01043 | DNA repair protein rad8 | -1.00 | DNA repair |
| **TRANSCRIPTION and RNA PROCESSING** | | | | |
|  | PAAG_08071 | DNA-directed RNA polymerase III subunit RPC2 | -2.12 | rRNA synthesis |
|  | PAAG_08627 | C6 zinc finger domain-containing protein | -1.94 | transcription |
|  | PAAG_08716 | tyrosyl-tRNA synthetase | -1.58 | mRNA synthesis |
|  | PAAG_08202 | IBR domain-containing protein | -1.49 | mRNA synthesis |
|  | PAAG_02384 | inositol polyphosphate multikinase | -1.42 | transcription regulation |
|  | PAAG_00698 | nitrogen regulatory protein OTam | -1.17 | transcription |
|  | PAAG_06150 | DEAD-box type RNA helicase | -1.16 | mRNA processes |
|  | PAAG_00248 | small nucleolar ribonucleoprotein complex subunit Utp14 | -1.14 | rRNA processing |
|  | PAAG_07160 | transcriptional corepressor of histone genes (Hir3) | -1.14 | transcriptional control |
|  | PAAG_05064 | transcription factor prr1 | -1.13 | transcriptional control |
|  | PAAG_08490 | C6 zinc finger domain-containing protein | -1.07 | transcription |
|  | PAAG_07090 | C2H2 transcription factor (Rpn4) | -1.07 | transcriptional control |
|  | PAAG_00672 | MRS7 family protein | -1.04 | splicing |
|  | PAAG_03973 | RNA binding protein MSSP-2 | -1.03 | transcription |
|  | PAAG_01159 | U3 snoRNP protein Utp20 | -1.00 | rRNA processing |
| **TRANSLATION and RIBOSSOME BIOGENESIS** | | | | |
|  | PAAG_08348 | eukaryotic translation initiation factor 3 39 kDa subunit | -1.72 | translation initiation |
|  | PAAG_04010 | ankyrin repeat protein | -1.32 | ribosome biogenesis |
|  | PAAG_08930 | 60S ribosomal protein L20 | -1.17 | ribosome biogenesis |
|  | PAAG_04916 | elongation factor G | -1.12 | translation elongation |
|  | PAAG_06520 | translation initiation factor eIF4G | -1.11 | translation initiation |
|  | PAAG_07876 | translation initiation factor IF-2 | -1.08 | translation initiation |
| **PROTEIN FATE** | | | | |
|  | PAAG_03385 | non-classical export protein Nce102 | -2.38 | protein targeting, sorting and translocation |
|  | PAAG_08382 | Lamina-associated polypeptide 2 | -2.23 | protein processing (proteolytic) |
|  | PAAG_01201 | WD repeat-containing protein | -1.84 | protein/peptide degradation |
|  | PAAG_05297 | ubiquitin carboxyl-terminal hydrolase | -1.81 | assembly of protein complexes |
|  | PAAG_04661 | A-factor-processing enzyme | -1.76 | protein/peptide degradation |
|  | PAAG_02507 | pre-mRNA-splicing factor cwc23 | -1.63 | protein folding and stabilization |
|  | PAAG_00155 | autophagy-related protein 17 | -1.30 | protein/peptide degradation |
|  | PAAG_05092 | ubiquitin-specific protease | -1.24 | modification by ubiquitination, deubiquitination |
|  | PAAG_05845 | F-box domain-containing protein | -1.22 | protein/peptide degradation |
|  | PAAG_02130 | heat shock protein HSP98 | -1.20 | protein/peptide degradation |
|  | PAAG_04555 | sarcosine oxidase | -1.20 | protein modification |
|  | PAAG_06287 | carboxypeptidase S1 | -1.17 | protein/peptide degradation |
|  | PAAG_02427 | serine/threonine-protein kinase ppk8 | -1.12 | modification by phosphorylation, dephosphorylation, autophosphorylation |
|  | PAAG_08458 | peptidyl-prolyl isomerase cwc27 | -1.06 | protein folding and stabilization |
|  | PAAG_05144 | ubiquitin-protein ligase | -1.05 | modification by ubiquitination, deubiquitination |
|  | PAAG_08133 | E3 ubiquitin-protein ligase UPL3 | -1.03 | modification by ubiquitination, deubiquitination |
|  | PAAG_01784 | phosphatase | -1.02 | modification by phosphorylation, dephosphorylation, autophosphorylation |
|  | PAAG_04772 | J-type co-chaperone JAC1 | -1.02 | protein folding and stabilization |
| **BINDING** | | | | |
|  | PAAG_07154 | copper-transporting P-type ATPase | -2.00 | heavy metal binding (Cu, Fe, Zn) |
|  | PAAG_03184 | multidrug resistance-associated protein | -1.80 | ATP binding |
|  | PAAG_00635 | ABC transporter CDR4 | -1.51 | ATP binding |
|  | PAAG_05482 | meiotically up-regulated gene 62 protein | -1.46 | protein binding |
|  | PAAG_06985 | calponin | -1.45 | protein binding |
|  | PAAG_01157 | cwfJ domain-containing protein | -1.23 | binding |
| **TRANSPORT** | | | | |
|  | PAAG_03058 | high-affinity methionine permease | -5.64 | methionine transport |
|  | PAAG_01621 | general amino acid permease AGP3 | -4.33 | amino acid/amino acid derivatives transport |
|  | PAAG_03965 | high-affinity permease for basic amino acids | -3.24 | amino acid/amino acid derivatives transport |
|  | PAAG_03520 | arrestin | -3.00 | protein transport |
|  | PAAG_01023 | MFS multidrug transporter | -2.71 | drug/toxin transport |
|  | PAAG_07892 | caffeine resistance protein | -2.55 | drug/toxin transport |
|  | PAAG_06133 | fluconazole resistance protein | -2.17 | drug/toxin transport |
|  | PAAG_07191 | potassium transporter 5 | -2.09 | cation transport (H+, Na+, K+, Ca2+ , NH4+, etc.) |
|  | PAAG_03577 | ABC drug exporter AtrF | -1.89 | drug/toxin transport |
|  | PAAG_07108 | regulator of V-ATPase in vacuolar membrane protein | -1.85 | peptide transport |
|  | PAAG_05816 | MFS monocarboxylate transporter | -1.74 | transport |
|  | PAAG_00519 | uracil permease | -1.56 | nucleotide/nucleoside/nucleobase transport |
|  | PAAG_05584 | peroxisomal adenine nucleotide transporter 1 | -1.56 | proton transport |
|  | PAAG_05884 | MFS monocarboxylate transporter | -1.55 | transport |
|  | PAAG_03790 | calcium-transporting ATPase | -1.54 | cation transport (H+, Na+, K+, Ca2+ , NH4+, etc.) |
|  | PAAG_08232 | P-type ATPase | -1.50 | transport ATPases |
|  | PAAG_07228 | vacuolar assembly protein | -1.42 | vacuolar/lysosomal transport |
|  | PAAG_02796 | mitochondrial 2-oxodicarboxylate carrier 1 | -1.41 | C-compound and carbohydrate transport |
|  | PAAG_04459 | arginine permease | -1.31 | amino acid/amino acid derivatives transport |
|  | PAAG_06074 | Amt4 protein | -1.30 | amino transport |
|  | PAAG_07186 | calcium-transporting ATPase | -1.26 | cation transport (H+, Na+, K+, Ca2+ , NH4+, etc.) |
|  | PAAG_07726 | nitrate transporter | -1.24 | cation transport (H+, Na+, K+, Ca2+ , NH4+, etc.) |
|  | PAAG_02009 | sugar transporter | -1.19 | C-compound and carbohydrate transport |
|  | PAAG_09040 | FAD-binding 8 | -1.18 | homeostasis of metal ions (Na, K, Ca etc.) |
|  | PAAG_01167 | proline-specific permease | -1.13 | amino acid/amino acid derivatives transport |
|  | PAAG_03202 | mannose-P-dolichol utilization defect 1 protein | -1.13 | transport |
|  | PAAG_05363 | cation diffusion facilitator 1 | -1.07 | cation transport (H+, Na+, K+, Ca2+ , NH4+, etc.) |
|  | PAAG_02166 | MFS multidrug transporter | -1.06 | drug/toxin transpot |
|  | PAAG_01645 | mechanosensitive ion channel family | -1.06 | transport |
| **SIGNAL TRANSDUCTION** | | | | |
|  | PAAG_04436 | guanine nucleotide-binding protein alpha-1 subunit | -3.29 | cellular signalling |
|  | PAAG_07740 | beta-chimaerin | -1.53 | GTPase activator (GAP) |
|  | PAAG_03876 | DENN domain-containing protein | -1.52 | signal transduction |
|  | PAAG_00861 | RhoGAP domain-containing protein | -1.49 | GTPase activator (GAP) |
|  | PAAG_03936 | ubiquitin conjugating enzyme | -1.47 | enzymatic activity regulation / enzyme regulator |
|  | PAAG_07613 | cAMP-mediated signaling protein Sok1 | -1.44 | cAMP mediated-signaling |
|  | PAAG_04354 | Ras guanine-nucleotide exchange protein | -1.29 | regulator of G-protein signalling |
|  | PAAG_01440 | kinase domain-containing protein | -1.28 | signal transduction |
|  | PAAG_06776 | peptide methionine sulfoxide reductase msrA | -1.18 | regulation by modification |
| **CELL RESCUE, DEFENSE and VIRULENCE** | | | | |
|  | PAAG_01465 | carbonic anhydrase | -3.66 | stress response/ carbon utilization |
|  | PAAG_03216 | mitochondrial peroxiredoxin PRX1 | -3.50 | oxidative stress response |
|  | PAAG_05763 | acid phosphatase | -3.19 | osmotic and salt stress response |
|  | PAAG_06538 | CAP20 | -2.92 | virulence. disease factors |
|  | PAAG_04424 | peroxiredoxin HYR1 | -2.11 | oxidative stress response |
|  | PAAG_01298 | M protein repeat protein | -1.02 | virulence |
| **CELL GROWTH/ MORPHOGENESIS** | | | | |
|  | PAAG_07251 | apoptosis-inducing factor 1 | -2.59 | apoptosis |
|  | PAAG_09030 | MYB DNA-binding domain-containing protein | -1.56 | cell proliferation and differentiation |
|  | PAAG_06795 | MYB DNA-binding domain-containing protein | -1.49 | cell proliferation and differentiation |
|  | PAAG_03245 | telomere length regulator protein | -1.46 | organization of chromosome structure |
|  | PAAG_05127 | kelch-domain-containing protein | -1.42 | directional cell growth (morphogenesis) |
|  | PAAG_01545 | D-alanine-poly(phosphoribitol) ligase subunit 1 | -1.35 | [cell wall biogenesis](http://www.uniprot.org/keywords/KW-0961) |
|  | PAAG_00653 | SNF2 family helicase/ATPase | -1.23 | [ATP-dependent chromatin remodeling](http://www.ebi.ac.uk/QuickGO/GTerm?id=GO:0043044) |
|  | PAAG_06965 | ISWI chromatin-remodeling complex ATPase ISW1 | -1.09 | organization of chromosome structure |
|  | PAAG_04008 | pheromone-regulated membrane protein | -1.04 | mating (fertilization) |
|  | PAAG_02937 | mitochondrial dynamin GTPase (Msp1) | -1.01 | mitochondrion |
| **MISCELLANEOUS** | | | | |
|  | PAAG_01057 | taurine catabolism dioxygenase TauD | -3.45 | oxidation-reduction process |
|  | PAAG_03233 | oxidoreductase | -1.15 | oxidation-reduction process |
| **UNCLASSIFIED** | | | | |
|  | PAAG_03179 | hypothetical protein | -7.02 | - |
|  | PAAG_02855 | conserved hypothetical protein | -6.28 | - |
|  | PAAG_05037 | HHE domain-containing protein | -6.18 | - |
|  | PAAG_05351 | conserved hypothetical protein | -4.20 | - |
|  | PAAG_04990 | conserved hypothetical protein | -4.18 | - |
|  | PAAG_06308 | predicted protein | -3.86 | - |
|  | PAAG_05603 | conserved hypothetical protein | -3.78 | - |
|  | PAAG_04329 | predicted protein | -3.77 | - |
|  | PAAG_01058 | conserved domain-containing protein | -3.51 | - |
|  | PAAG_03519 | predicted protein | -3.33 | - |
|  | PAAG_04940 | conserved hypothetical protein | -3.33 | - |
|  | PAAG_02996 | hypothetical protein | -3.22 | - |
|  | PAAG_00216 | predicted protein | -3.00 | - |
|  | PAAG_07723 | predicted protein | -2.94 | - |
|  | PAAG_01815 | predicted protein | -2.94 | - |
|  | PAAG_02991 | conserved hypothetical protein | -2.91 | - |
|  | PAAG_02616 | conserved hypothetical protein | -2.88 | - |
|  | PAAG_02999 | predicted protein | -2.74 | - |
|  | PAAG_00251 | hypothetical protein | -2.70 | - |
|  | PAAG_03875 | predicted protein | -2.61 | - |
|  | PAAG_00270 | predicted protein | -2.58 | - |
|  | PAAG_02654 | predicted protein | -2.57 | - |
|  | PAAG_02540 | predicted protein | -2.54 | - |
|  | PAAG_01467 | conserved hypothetical protein | -2.49 | - |
|  | PAAG_02778 | conserved hypothetical protein | -2.47 | - |
|  | PAAG_03728 | hypothetical protein | -2.43 | - |
|  | PAAG_07912 | conserved hypothetical protein | -2.39 | - |
|  | PAAG_04360 | predicted protein | -2.38 | - |
|  | PAAG_05705 | conserved hypothetical protein | -2.34 | - |
|  | PAAG_02546 | conserved hypothetical protein | -2.34 | - |
|  | PAAG_04672 | predicted protein | -2.27 | - |
|  | PAAG_05136 | conserved hypothetical protein | -2.26 | - |
|  | PAAG_00252 | hypothetical protein | -2.24 | - |
|  | PAAG_03185 | hypothetical protein | -2.21 | - |
|  | PAAG_01721 | hypothetical protein | -2.20 | - |
|  | PAAG_02508 | conserved hypothetical protein | -2.20 | - |
|  | PAAG_07250 | predicted protein | -2.20 | - |
|  | PAAG_03528 | leucine rich repeat protein | -2.19 | - |
|  | PAAG_03457 | conserved hypothetical protein | -2.18 | - |
|  | PAAG_06755 | predicted protein | -2.18 | - |
|  | PAAG_03183 | conserved hypothetical protein | -2.16 | - |
|  | PAAG_01585 | predicted protein | -2.12 | - |
|  | PAAG_02223 | conserved hypothetical protein | -2.06 | - |
|  | PAAG_06775 | predicted protein | -2.04 | - |
|  | PAAG_08381 | predicted protein | -2.02 | - |
|  | PAAG_04588 | predicted protein | -2.01 | - |
|  | PAAG_05794 | conserved hypothetical protein | -1.98 | - |
|  | PAAG_05268 | predicted protein | -1.96 | - |
|  | PAAG_00129 | predicted protein | -1.96 | - |
|  | PAAG_08628 | conserved hypothetical protein | -1.94 | - |
|  | PAAG_07641 | conserved hypothetical protein | -1.94 | - |
|  | PAAG_04044 | predicted protein | -1.93 | - |
|  | PAAG_08383 | conserved hypothetical protein | -1.92 | - |
|  | PAAG_07173 | conserved hypothetical protein | -1.89 | - |
|  | PAAG_04560 | conserved hypothetical protein | -1.88 | - |
|  | PAAG_01848 | predicted protein | -1.88 | - |
|  | PAAG_04551 | hypothetical protein | -1.87 | - |
|  | PAAG_07621 | predicted protein | -1.81 | - |
|  | PAAG_00652 | conserved hypothetical protein | -1.80 | - |
|  | PAAG_08719 | conserved hypothetical protein | -1.79 | - |
|  | PAAG_01708 | predicted protein | -1.79 | - |
|  | PAAG_03872 | predicted protein | -1.79 | - |
|  | PAAG_02242 | hypothetical protein | -1.77 | - |
|  | PAAG_04087 | predicted protein | -1.77 | - |
|  | PAAG_05741 | predicted protein | -1.77 | - |
|  | PAAG_04793 | conserved hypothetical protein | -1.76 | - |
|  | PAAG_05958 | predicted protein | -1.74 | - |
|  | PAAG_08480 | hypothetical protein | -1.73 | - |
|  | PAAG_00307 | conserved hypothetical protein | -1.73 | - |
|  | PAAG_08072 | conserved hypothetical protein | -1.72 | - |
|  | PAAG_07977 | predicted protein | -1.71 | - |
|  | PAAG_04088 | conserved hypothetical protein | -1.70 | - |
|  | PAAG_05181 | conserved leucine-rich repeat protein | -1.70 | - |
|  | PAAG_07772 | conserved hypothetical protein | -1.70 | - |
|  | PAAG_04750 | conserved hypothetical protein | -1.70 | - |
|  | PAAG_03283 | predicted protein | -1.68 | - |
|  | PAAG_03844 | predicted protein | -1.68 | - |
|  | PAAG_06521 | predicted protein | -1.66 | - |
|  | PAAG_08768 | conserved hypothetical protein | -1.66 | - |
|  | PAAG_04994 | predicted protein | -1.66 | - |
|  | PAAG_04409 | conserved hypothetical protein | -1.65 | - |
|  | PAAG_00420 | hypothetical protein | -1.64 | - |
|  | PAAG_01003 | hypothetical protein | -1.64 | - |
|  | PAAG_07788 | conserved hypothetical protein | -1.64 | - |
|  | PAAG_02385 | predicted protein | -1.61 | - |
|  | PAAG_02240 | hypothetical protein | -1.60 | - |
|  | PAAG_05593 | predicted protein | -1.56 | - |
|  | PAAG_06729 | predicted protein | -1.56 | - |
|  | PAAG_04536 | predicted protein | -1.56 | - |
|  | PAAG_03152 | CobW domain-containing protein | -1.55 | - |
|  | PAAG_06974 | hypothetical protein | -1.55 | - |
|  | PAAG_08929 | hypothetical protein | -1.54 | - |
|  | PAAG_00156 | predicted protein | -1.54 | - |
|  | PAAG_00451 | predicted protein | -1.54 | - |
|  | PAAG_04585 | hypothetical protein | -1.53 | - |
|  | PAAG_04437 | hypothetical protein | -1.53 | - |
|  | PAAG_03351 | conserved hypothetical protein | -1.51 | - |
|  | PAAG_01849 | predicted protein | -1.51 | - |
|  | PAAG_02899 | hypothetical protein | -1.48 | - |
|  | PAAG_04256 | conserved hypothetical protein | -1.48 | - |
|  | PAAG_00250 | conserved hypothetical protein | -1.48 | - |
|  | PAAG_00820 | predicted protein | -1.48 | - |
|  | PAAG_08333 | predicted protein | -1.45 | - |
|  | PAAG_01730 | predicted protein | -1.45 | - |
|  | PAAG_00796 | predicted protein | -1.44 | - |
|  | PAAG_07928 | hypothetical protein | -1.42 | - |
|  | PAAG_07649 | conserved hypothetical protein | -1.41 | - |
|  | PAAG_05456 | predicted protein | -1.40 | - |
|  | PAAG_03074 | predicted protein | -1.39 | - |
|  | PAAG_07685 | predicted protein | -1.39 | - |
|  | PAAG_04834 | predicted protein | -1.38 | - |
|  | PAAG_02424 | predicted protein | -1.38 | - |
|  | PAAG_01116 | predicted protein | -1.38 | - |
|  | PAAG_07898 | hypothetical protein | -1.37 | - |
|  | PAAG_02572 | conserved hypothetical protein | -1.37 | - |
|  | PAAG_06832 | predicted protein | -1.36 | - |
|  | PAAG_04163 | predicted protein | -1.35 | - |
|  | PAAG_05654 | predicted protein | -1.35 | - |
|  | PAAG_07533 | conserved hypothetical protein | -1.34 | - |
|  | PAAG_01925 | conserved hypothetical protein | -1.34 | - |
|  | PAAG_07306 | predicted protein | -1.34 | - |
|  | PAAG_02489 | conserved hypothetical protein | -1.33 | - |
|  | PAAG_04951 | predicted protein | -1.33 | - |
|  | PAAG_05923 | conserved hypothetical protein | -1.33 | - |
|  | PAAG_04289 | predicted protein | -1.32 | - |
|  | PAAG_06875 | conserved hypothetical protein | -1.32 | - |
|  | PAAG_01719 | conserved hypothetical protein | -1.30 | - |
|  | PAAG_03218 | predicted protein | -1.30 | - |
|  | PAAG_06036 | conserved hypothetical protein | -1.29 | - |
|  | PAAG_03234 | predicted protein | -1.29 | - |
|  | PAAG_07604 | hypothetical protein | -1.29 | - |
|  | PAAG_00632 | predicted protein | -1.28 | - |
|  | PAAG_07405 | predicted protein | -1.28 | - |
|  | PAAG_08154 | conserved hypothetical protein | -1.28 | - |
|  | PAAG_02493 | predicted protein | -1.27 | - |
|  | PAAG_08515 | hypothetical protein | -1.27 | - |
|  | PAAG_01263 | predicted protein | -1.26 | - |
|  | PAAG_06650 | predicted protein | -1.26 | - |
|  | PAAG_01478 | predicted protein | -1.26 | - |
|  | PAAG_06272 | predicted protein | -1.26 | - |
|  | PAAG_02947 | predicted protein | -1.26 | - |
|  | PAAG_01004 | predicted protein | -1.25 | - |
|  | PAAG_01250 | conserved hypothetical protein | -1.25 | - |
|  | PAAG_00303 | hypothetical protein | -1.25 | - |
|  | PAAG_06834 | conserved hypothetical protein | -1.24 | - |
|  | PAAG_01303 | conserved hypothetical protein | -1.24 | - |
|  | PAAG_03384 | predicted protein | -1.23 | - |
|  | PAAG_01021 | predicted protein | -1.23 | - |
|  | PAAG_05122 | conserved hypothetical protein | -1.23 | - |
|  | PAAG_03097 | predicted protein | -1.22 | - |
|  | PAAG_00674 | conserved hypothetical protein | -1.22 | - |
|  | PAAG_02852 | conserved hypothetical protein | -1.22 | - |
|  | PAAG_07820 | predicted protein | -1.22 | - |
|  | PAAG_09020 | conserved hypothetical protein | -1.22 | - |
|  | PAAG_04122 | predicted protein | -1.20 | - |
|  | PAAG_03666 | predicted protein | -1.19 | - |
|  | PAAG_08254 | predicted protein | -1.19 | - |
|  | PAAG_04625 | predicted protein | -1.19 | - |
|  | PAAG_01486 | hypothetical protein | -1.19 | - |
|  | PAAG_05531 | conserved hypothetical protein | -1.19 | - |
|  | PAAG_08542 | WD40 domain-containing protein | -1.18 | - |
|  | PAAG_07858 | predicted protein | -1.17 | - |
|  | PAAG_09021 | conserved hypothetical protein | -1.17 | - |
|  | PAAG_01005 | hypothetical protein | -1.16 | - |
|  | PAAG_03582 | predicted protein | -1.16 | - |
|  | PAAG_00442 | predicted protein | -1.15 | - |
|  | PAAG_05907 | predicted protein | -1.14 | - |
|  | PAAG_06833 | conserved hypothetical protein | -1.14 | - |
|  | PAAG_08496 | predicted protein | -1.13 | - |
|  | PAAG_07719 | conserved hypothetical protein | -1.13 | - |
|  | PAAG_04889 | conserved hypothetical protein | -1.13 | - |
|  | PAAG_02707 | hypothetical protein | -1.13 | - |
|  | PAAG_02035 | protein EFR3 | -1.12 | [-](http://www.ebi.ac.uk/QuickGO/GTerm?id=GO:0090002) |
|  | PAAG_00167 | predicted protein | -1.12 | - |
|  | PAAG_09038 | predicted protein | -1.11 | - |
|  | PAAG_04024 | predicted protein | -1.11 | - |
|  | PAAG_05063 | predicted protein | -1.10 | - |
|  | PAAG_06277 | conserved hypothetical protein | -1.09 | - |
|  | PAAG_01297 | predicted protein | -1.09 | - |
|  | PAAG_05843 | predicted protein | -1.08 | - |
|  | PAAG_07642 | hypothetical protein | -1.08 | - |
|  | PAAG_02539 | conserved hypothetical protein | -1.08 | - |
|  | PAAG_08189 | predicted protein | -1.08 | - |
|  | PAAG_02474 | predicted protein | -1.08 | - |
|  | PAAG_07633 | conserved hypothetical protein | -1.08 | - |
|  | PAAG_08810 | hypothetical protein | -1.08 | - |
|  | PAAG_07684 | hypothetical protein | -1.07 | - |
|  | PAAG_07752 | predicted protein | -1.07 | - |
|  | PAAG_03597 | predicted protein | -1.07 | - |
|  | PAAG_00846 | conserved hypothetical protein | -1.07 | - |
|  | PAAG_06746 | predicted protein | -1.07 | - |
|  | PAAG_08985 | conserved hypothetical protein | -1.07 | - |
|  | PAAG_04727 | predicted protein | -1.07 | - |
|  | PAAG_01994 | predicted protein | -1.07 | - |
|  | PAAG_02650 | conserved hypothetical protein | -1.06 | - |
|  | PAAG_06704 | hypothetical protein | -1.05 | - |
|  | PAAG_01169 | predicted protein | -1.04 | - |
|  | PAAG_06499 | predicted protein | -1.03 | - |
|  | PAAG_07261 | predicted protein | -1.03 | - |
|  | PAAG_02224 | predicted protein | -1.03 | - |
|  | PAAG_06732 | predicted protein | -1.02 | - |
|  | PAAG_08771 | conserved hypothetical protein | -1.02 | - |
|  | PAAG_06315 | conserved hypothetical protein | -1.02 | - |
|  | PAAG_04922 | predicted protein | -1.00 | - |
|  | PAAG_07226 | predicted protein | -1.00 | - |
|  | PAAG_08536 | predicted protein | -1.00 | - |
|  | PAAG_08103 | EF hand domain-containing protein | -1.00 | - |
|  | PAAG_03575 | predicted protein | -1.00 | - |
|  | PAAG_01202 | predicted protein | -1.00 | - |

^a^ Identification of differentially regulated transcripts from *Paracoccidioides* genome database (<http://www.broadinstitute.org/annotation/genome/paracoccidioides_brasiliensis/MultiHome.html>).

^b^ Transcripts annotation from *Paracoccidioides* genome database or by homology from NCBI database (<http://www.ncbi.nlm.nih.gov/>);

^c^ Transcripts expression profiles in log2_fold change obtained from fold change selection method for differentially expressed transcripts using a Fisher exact test with a p-value of 0.001.

^d^ Biological process of differentially expressed transcripts from MIPS

(<http://pedant.helmholtz-muenchen.de/pedant3htmlview/pedant3view?Method=analysis&Db=p3_r48325_Par_brasi_Pb01>).
