# Supplementary material for: Transcriptional and Proteomic Responses to Carbon Starvation in Paracoccidioides
Source: PLoS Negl Trop Dis. 2014 May 8;8(5):e2855. doi: 10.1371/journal.pntd.0002855 (PMC4014450; doi:10.1371/journal.pntd.0002855)
Supplement: Table S5 — Up-regulated proteins of Paracoccidioides ( Pb 01) yeast cells under carbon starvation detected using NanoUPLC-MSE. (DOC) [file pntd.0002855.s016.doc]

**Table S5. Up-regulated proteins of *Paracoccidioides* (*Pb*01) yeast cells under carbon starvation detected using NanoUPLC-MS^E^.**

|  | **ID^a^** | **Annotation^b^** | **Peptides AVG^c^** | **Score AVG^c^** | **Fold change^d^** | **Biological process^e^** |  |
| --- | --- | --- | --- | --- | --- | --- | --- |
| **METABOLISM** | | | | | | |  |
| **Amino acid metabolism** | | | | | | |  |
|  | PAAG_08164 | homogentisate 1.2-dioxygenase | 10.4 | 1743.5 | 1.00 | tyrosine degradation |  |
|  | PAAG_07626 | Cobalamin-independent synthase | 21.5 | 4029.9 | 0.98 | methionine biosynthesis |  |
|  | PAAG_05776 | dihydroxy-acid dehydratase | 13.0 | 553.0 | 0.97 | isoleucine biosynthesis |  |
|  | PAAG_05253 | delta-1-pyrroline-5-carboxylate dehydrogenase | 17.0 | 1560.5 | 0.87 | glutamate degradation |  |
|  | PAAG_02859 | adenosylhomocysteinase | 19.0 | 4627.3 | 0.76 | methionine biosynthesis |  |
|  | PAAG_08701 | D-3-phosphoglycerate dehydrogenase | 11.3 | 259.5 | 0.75 | serine biosynthesis |  |
|  | PAAG_00966 | L-threonine 3-dehydrogenase | 8.0 | 750.6 | 0.74 | threonine degradation |  |
|  | PAAG_05048 | 3-isopropylmalate dehydratase large subunit | 24.7 | 2036.2 | 0.69 | leucine biosynthesis |  |
|  | PAAG_01568 | glycine dehydrogenase | 24.5 | 1035.1 | 0.59 | glycine biosynthesis |  |
|  | PAAG_08166 | 4-hydroxyphenylpyruvate dioxygenase | 9.0 | 398.3 | # | glycine biosynthesis |  |
|  | PAAG_03138 | alanine-glyoxylate aminotransferase | 7.0 | 250.0 | # | glycine biosynthesis |  |
|  | PAAG_01969 | arginase | 6.0 | 296.6 | # | glutamate biosynthesis |  |
|  | PAAG_01144 | aspartate aminotransferase | 8.5 | 269.7 | # | arginine biosynthesis |  |
|  | PAAG_01365 | choline dehydrogenase | 10.0 | 368.6 | # | glycine biosynthesis |  |
|  | PAAG_08649 | cysteine dioxygenase | 3.0 | 559.9 | # | cysteine degradation |  |
|  | PAAG_07954 | gamma-glutamyl phosphate reductase | 9.5 | 243.9 | # | proline biosynthesis |  |
|  | PAAG_03506 | glutamate decarboxylase | 5.3 | 228.8 | # | glutamate degradation |  |
|  | PAAG_07998 | glutamate synthase small chain | 10.0 | 184.4 | # | glutamate biosynthesis |  |
|  | PAAG_07317 | PENR2 protein | 3.0 | 185.2 | # | methionine biosynthesis |  |
|  | PAAG_08162 | maleylacetoacetate isomerase | 3.7 | 4457.3 | # | phenylalanine degradation |  |
|  | PAAG_04099 | methylcrotonoyl-CoA carboxylase subunit alpha | 12.0 | 249.7 | # | leucine degradation |  |
|  | PAAG_02693 | saccharopine dehydrogenase | 6.0 | 382.4 | # | lysine biosynthesis |  |
|  | PAAG_02901 | S-adenosylmethionine synthetase | 5.7 | 653.6 | # | L-methionine biosynthesis |  |
|  | PAAG_06416 | alanine racemase family protein | 12.0 | 819.0 | # | D- alanine biosynthethic process |  |
| **Nitrogen and sulfur metabolism** | | | | | | |  |
|  | PAAG_03333 | formamidase | 11.3 | 6047.4 | 2.05 | nitrogen metabolism |  |
|  | PAAG_07811 | sulfite oxidase | 8.7 | 1201.7 | # | nitrogen. sulfur and selenium metabolism |  |
| **C-compound and carbohydrate metabolism** | | | | | | |  |
|  | PAAG_06953 | short chain dehydrogenase/reductase family | 6.0 | 1687.7 | 2.96 | carbohydrate metabolism_sugar epimerase family |  |
|  | PAAG_07276 | glycogen synthase | 14.3 | 301.0 | 1.77 | carbohydrate metabolism |  |
|  | PAAG_04549 | mitochondrial 2-methylisocitrate lyase | 11.8 | 258.0 | 1.05 | C-compound metabolism_proprionate degradation |  |
|  | PAAG_06473 | mannitol-1-phosphate 5-dehydrogenase | 13.2 | 2172.9 | 0.71 | C-compound and carbohydrate metabolism |  |
|  | PAAG_04181 | sorbitol utilization protein SOU2 | 4.3 | 524.9 | # | C-compound and carbohydrate metabolism |  |
|  | PAAG_02653 | acetyl-coenzyme A synthetase | 14.0 | 498.7 | # | C-compound and carbohydrate metabolism |  |
|  | PAAG_00685 | alpha-mannosidase | 18.3 | 311.3 | # | polysaccharide  metabolism |  |
|  | PAAG_01935 | formyl-coenzyme A transferase | 8.0 | 264.0 | # | oxalate catabolic process |  |
|  | PAAG_05984 | glutaryl-CoA dehydrogenase | 11.0 | 468.8 | # | aromatic hydrocarbons catabolism |  |
|  | PAAG_03765 | NADP-dependent glycerol dehydrogenase | 4.0 | 349.7 | # | Sugar, glucoside, polyol and carboxylate catabolism |  |
|  | PAAG_05416 | NADP-dependent leukotriene B4 12-hydroxydehydrogenase | 4.0 | 316.9 | # | C-compound and carbohydrate metabolism |  |
|  | PAAG_06057 | aldose 1-epimerase | 4.0 | 315.4 | # | hexose metabolic process |  |
|  | PAAG_02162 | lactam utilization protein LamB | 4.0 | 383.0 | # | C-compound and carbohydrate metabolism |  |
|  | PAAG_01870 | short-chain dehydrogenase/reductase SDR | 7.0 | 203.0 | # | Carbohydrate metabolism_sugar epimerase family |  |
|  | PAAG_03316 | polysaccharide export protein | 8.0 | 227.5 | # | surface polysaccharide biosynthesis process |  |
| **Lipid, fatty acid and isoprenoid metabolism** | | | | | | |  |
|  | PAAG_02163 | acetyl-/propionyl-coenzyme A carboxylase alpha chain | 23.5 | 736.8 | 3.03 | lipid metabolism |  |
|  | PAAG_05249 | aldehyde dehydrogenase | 17.0 | 6533.0 | 2.45 | lipid and fatty acid metabolism_oxidation |  |
|  | PAAG_06309 | enoyl-CoA hydratase | 9.7 | 3668.3 | 1.24 | lipid and fatty acid metabolism_beta oxidation |  |
|  | PAAG_02664 | 3-ketoacyl-CoA thiolase | 15.2 | 6432.7 | 1.15 | lipid and fatty acid metabolism_beta oxidation |  |
|  | PAAG_03960 | isopentenyl-diphosphate Delta-isomerase | 6.5 | 301.6 | 1.00 | sterol biosynthesis |  |
|  | PAAG_03447 | acetyl-CoA acetyltransferase (acetoacetyl-CoA thiolase) | 11.3 | 487.6 | 0.79 | ergosterol biosynthesis |  |
|  | PAAG_01928 | peroxisomal dehydratase | 7.7 | 209.9 | # | Lipid, fatty acid and isoprenoid metabolism |  |
|  | PAAG_06099 | glycerol-3-phosphate dehydrogenase | 9.0 | 238.4 | # | lipid metabolism |  |
|  | PAAG_01833 | 2-succinylbenzoate-CoA ligase | 9.7 | 402.8 | # | fatty acid metabolism |  |
|  | PAAG_06224 | carnitine O-acetyltransferase | 13.7 | 359.7 | # | Lipid, fatty acid and isoprenoid metabolism |  |
|  | PAAG_07279 | farnesyl pyrophosphate synthetase | 5.0 | 175.2 | # | lipid metabolism |  |
|  | PAAG_04142 | NAD-dependent 15-hydroxyprostaglandin dehydrogenase | 5.0 | 166.0 | # | lipid metabolism |  |
|  | PAAG_04030 | short-chain-fatty-acid-CoA ligase | 10.0 | 183.8 | # | lipid and fatty acid metabolism |  |
| **Purin nucleotide/ nucleoside/ nucleobase metabolism** | | | | | | |  |
|  | PAAG_02115 | ribose-phosphate pyrophosphokinase | 10.5 | 838.7 | 1.82 | purin nucleotide/nucleoside/nucleobase metabolism |  |
|  | PAAG_05611 | deoxyuridine 5'-triphosphate nucleotidohydrolase | 5.7 | 5794.4 | 0.94 | pyrimidine nucleotide/nucleoside/nucleobase metabolism |  |
|  | PAAG_01457 | xanthine phosphoribosyltransferase 1 | 8.3 | 431.4 | 0.58 | purin nucleotide/nucleoside/nucleobase metabolism |  |
|  | PAAG_09072 | mitochondrial nuclease | 7.0 | 475.2 | # | polynucleotide degradation |  |
|  | PAAG_04974 | adenylosuccinate lyase | 13.5 | 217.1 | # | purine biosynthesis |  |
|  | PAAG_05803 | inosine-5'-monophosphate dehydrogenase IMD2 | 10.0 | 358.0 | # | purine nucleotide/nucleoside/nucleobase anabolism |  |
|  | PAAG_08856 | nicotinate-nucleotide pyrophosphorylase | 6.0 | 463.2 | # | biosynthesis of the pyridine nucleotides NAD and NADP |  |
|  | PAAG_02633 | ribose-phosphate pyrophosphokinase | 10.0 | 501.1 | # | nucleotide biosynthesis |  |
|  | PAAG_01751 | [cytidine deaminase](http://www.ncbi.nlm.nih.gov/blast/Blast.cgi#alnHdr_226288225) | 3.0 | 252.8 | # | UMP synthesis |  |
| **Secundary metabolism** | | | | | | |  |
|  | PAAG_02336 | [nudix hydrolase](http://www.ncbi.nlm.nih.gov/blast/Blast.cgi#alnHdr_225684341) | 9.0 | 236.6 | 1.27 | metabolism of vitamins, cofactors, and prosthetic groups |  |
|  | PAAG_00851 | 6.7-dimethyl-8-ribityllumazine synthase | 4.0 | 923.0 | # | biosynthesis of riboflavin |  |
|  | PAAG_01324 | folic acid synthesis protein | 9.3 | 383.2 | # | folic acid-containing compound  biosynthetic process |  |
|  | PAAG_01934 | riboflavin synthase alpha chain | 6.5 | 346.6 | # | biosynthesis of riboflavin |  |
|  | PAAG_04443 | spermidine synthase | 7.5 | 397.5 | # | B complex vitamins |  |
|  | PAAG_02656 | NADH-ubiquinone oxidoreductase 51 kDa subunit | 12.0 | 185.5 | # | ubiquinone |  |
| **ENERGY** | | | | | | |  |
| **Glycolysis and gluconeogenesis** | | | | | | |  |
|  | PAAG_02011 | phosphoglucomutase (glucose-6-phosphatase) | 19.2 | 672.7 | 1.37 | glycolysis and gluconeogenesis |  |
|  | PAAG_01995 | fructose-bisphosphate aldolase | 13.8 | 4512.4 | 1.30 | glycolysis and gluconeogenesis |  |
|  | PAAG_08203 | phosphoenolpyruvate carboxykinase | 19.2 | 1604.9 | 1.30 | gluconeogenesis |  |
|  | PAAG_02682 | fructose-1,6-bisphosphatase | 7.8 | 870.5 | 0.82 | gluconeogenesis |  |
|  | PAAG_01583 | 6-phosphofructokinase subunit beta | 16.0 | 160.5 | # | glycolysis |  |
|  | PAAG_06172 | glucokinase | 10.5 | 303.6 | # | glycolysis |  |
| **Glyoxylate cycle** | | | | | | |  |
|  | PAAG_06951 | isocitrate lyase | 20.2 | 585.6 | 1.26 | glyoxylate cycle |  |
| **Tricarboxylic-acid pathway** | | | | | | |  |
|  | PAAG_02732 | 2-oxoglutarate dehydrogenase E1 | 19.3 | 279.3 | 0.62 | TCA cycle |  |
|  | PAAG_00588 | fumarate hydratase | 7.0 | 858.2 | # | TCA cycle |  |
|  | PAAG_04597 | malate dehydrogenase | 3.0 | 167.8 | # | TCA cycle |  |
| **Electron transport and membrane-associated energy conservation** | | | | | | |  |
|  | PAAG_07246 | [cytochrome-c oxidase chain VI](http://www.ncbi.nlm.nih.gov/blast/Blast.cgi#alnHdr_225684940) | 8.8 | 2156.6 | 0.61 | electron transport |  |
|  | PAAG_02656 | NADH-ubiquinone oxidoreductase 51 kDa subunit | 12.0 | 185.5 | # | electron transport |  |
|  | PAAG_04570 | ATP synthase D chain. mitochondrial | 6.0 | 310.5 | # | respiration |  |
|  | PAAG_05605 | ATP synthase delta  chain | 4.0 | 619.2 | # | respiration |  |
|  | PAAG_08088 | cytochrome b-c1 complex subunit 2 | 12.3 | 764.4 | # | respiration |  |
|  | PAAG_06268 | cytochrome c | 4.0 | 448.0 | # | electron transport |  |
|  | PAAG_00173 | electron transfer flavoprotein subunit alpha | 9.5 | 475.7 | # | electron transport |  |
|  | PAAG_03599 | formate dehydrogenase | 4.0 | 228.1 | # | electron transport |  |
|  | PAAG_01265 | [cytochrome b5](http://www.ncbi.nlm.nih.gov/blast/Blast.cgi#alnHdr_261205440) | 3.0 | 956.0 | # | electron transport |  |
|  | PAAG_06796 | [cytochrome c oxidase polypeptide IV](http://www.ncbi.nlm.nih.gov/blast/Blast.cgi#alnHdr_225562763) | 3.0 | 468.4 | # | electron transport |  |
| **Ethanol production** | | | | | | |  |
|  | PAAG_00403 | alcohol dehydrogenase | 16.7 | 14201.8 | 1.83 | alcohol fermentation |  |
|  | PAAG_02050 | pyruvate decarboxylase | 14.3 | 2588.5 | 1.30 | alcohol fermentation |  |
|  | PAAG_04541 | alcohol dehydrogenase | 9.0 | 1872.7 | 0.97 | alcohol fermentation |  |
|  | PAAG_02512 | pyruvate decarboxylase | 9.0 | 162.6 | # | alcohol fermentation |  |
|  | PAAG_08248 | alcohol dehydrogenase | 5.0 | 295.8 | # | alcohol fermentation |  |
| **Pentose Phosphate pathway** | | | | | | |  |
|  | PAAG_05621 | 6-phosphogluconolactonase | 7.5 | 364.2 | # | pentose-phosphate shunt |  |
|  | PAAG_05146 | ribose 5-phosphate isomerase A | 5.7 | 389.6 | # | pentose-phosphate shunt |  |
| **CELL CYCLE and DNA PROCESSING** | | | | | | |  |
|  | PAAG_06948 | mitogen-activated protein kinase | 6.5 | 214.6 | 2.13 | cell cycle arrest |  |
|  | PAAG_00783 | serine/threonine-protein phosphatase PP1 | 6.5 | 247.4 | 0.82 | [DNA replication checkpoint and cell budding](http://www.ebi.ac.uk/QuickGO/GTerm?id=GO:0000076) |  |
|  | PAAG_00923 | proliferating cell nuclear antigen | 11.0 | 5527.4 | 0.82 | DNA synthesis and replication |  |
|  | PAAG_07608 | DNA helicase | 17.0 | 204.8 | # | DNA repair |  |
|  | PAAG_08917 | histone H2a | 4.3 | 1234.5 | # | DNA processing |  |
|  | PAAG_07098 | histone H4.1 | 4.0 | 1748.0 | # | DNA processing |  |
|  | PAAG_00126 | histone H4.2 | 4.0 | 1560.1 | # | DNA processing |  |
|  | PAAG_08918 | late histone H2B.L4 | 6.0 | 543.0 | # | DNA processing |  |
|  | PAAG_05147 | mitotic checkpoint protein BUB3 | 8.0 | 196.8 | # | cell cycle control |  |
|  | PAAG_03054 | G2/M phase checkpoint control protein Sum2 | 13.0 | 234.3 | # | cell cycle control |  |
| **TRANSCRIPTION** | | | | | | |  |
|  | PAAG_02467 | Transcription initiation factor TFIID subunit 14 | 5.3 | 261.8 | 3.22 | transcription regulation |  |
|  | PAAG_06250 | nuclear cap-binding protein | 2.5 | 502.9 | # | mRNA processing |  |
|  | PAAG_05609 | C2H2 type zinc finger domain-containing protein | 6.0 | 228.5 | # | transcriptional control |  |
|  | PAAG_02268 | DNA-directed RNA polymerase I subunit RPA12 | 7.0 | 469.2 | # | rRNA synthesis |  |
|  | PAAG_05397 | DNA-directed RNA polymerase II subunit RPB1 | 21.0 | 206.5 | # | rRNA synthesis |  |
|  | PAAG_02255 | mRNA decapping hydrolase | 5.3 | 485.6 | # | mRNA processing |  |
|  | PAAG_02437 | U2 small nuclear ribonucleoprotein B | 5.0 | 591.2 | # | splicing |  |
| **TRANSLATION** | | | | | | |  |
|  | PAAG_06886 | zinc finger protein GIS2 | 3.3 | 1490.4 | 5.54 | positive regulation of translation |  |
|  | PAAG_02865 | translation initiation factor RLI1 | 13.0 | 250.2 | 3.92 | translation |  |
|  | PAAG_07385 | 60S ribosomal protein L23a | 3.4 | 1960.8 | 1.88 | ribosome biogenesis |  |
|  | PAAG_06882 | 40S ribosomal protein S24 | 5.5 | 778.4 | 1.33 | ribosome biogenesis |  |
|  | PAAG_01413 | 40S ribosomal protein S17 | 5.7 | 1505.2 | 1.01 | ribosome biogenesis |  |
|  | PAAG_00347 | 60S ribosomal protein L9-B | 9.2 | 978.2 | 0.91 | ribosome biogenesis |  |
|  | PAAG_00772 | eukaryotic translation initiation factor 3 subunit | 5.3 | 1132.9 | 0.72 | translation |  |
|  | PAAG_07707 | 60S ribosomal protein L10a | 7.0 | 321.0 | 0.61 | ribosome biogenesis |  |
|  | PAAG_05882 | translation factor SUI1 | 3.0 | 1251.1 | # | translation |  |
|  | PAAG_01834 | 60S ribosomal protein L16 | 6.7 | 948.3 | # | ribosome biogenesis |  |
|  | PAAG_04425 | 60S ribosomal protein L22 | 5.5 | 1795.5 | # | ribosome biogenesis |  |
|  | PAAG_05233 | 60S ribosomal protein L26 | 5.0 | 1144.5 | # | ribosome biogenesis |  |
|  | PAAG_01939 | 60S ribosomal protein L27-A | 4.0 | 855.8 | # | ribosome biogenesis |  |
|  | PAAG_08847 | 60S ribosomal protein L28 | 2.0 | 277.8 | # | ribosome biogenesis |  |
|  | PAAG_06627 | 60S ribosomal protein L32 | 2.0 | 460.8 | # | ribosome biogenesis |  |
|  | PAAG_00648 | 60S ribosomal protein L33-B | 3.0 | 1502.2 | # | ribosome biogenesis |  |
|  | PAAG_06569 | 60S ribosomal protein L43 | 4.7 | 4081.0 | # | ribosome biogenesis |  |
|  | PAAG_03019 | 60S ribosomal protein L6-B | 6.0 | 279.3 | # | ribosome biogenesis |  |
|  | PAAG_04998 | 60S ribosomal protein L8-B | 5.5 | 1256.6 | # | ribosome biogenesis |  |
|  | PAAG_08285 | 50S ribosomal protein L12 | 2.0 | 257.5 | # | ribosome biogenesis |  |
|  | PAAG_01435 | 40S ribosomal protein S16 | 5.7 | 2012.5 | # | ribosome biogenesis |  |
|  | PAAG_03322 | 40S ribosomal protein S20 | 2.7 | 993.4 | # | ribosome biogenesis |  |
|  | PAAG_00385 | 40S ribosomal protein S23 | 4.7 | 801.4 | # | ribosome biogenesis |  |
|  | PAAG_03816 | 40S ribosomal protein S4 | 13.3 | 1061.9 | # | ribosome biogenesis |  |
|  | PAAG_01050 | cytosolic large ribosomal subunit protein L30 | 1.3 | 2218.3 | # | ribosome biogenesis |  |
|  | PAAG_07028 | histidyl-tRNA synthetase | 20.0 | 232.6 | # | translation |  |
|  | PAAG_08172 | lysyl-tRNA synthetase | 9.7 | 424.1 | # | translation |  |
|  | PAAG_03951 | prolyl-tRNA synthetase | 13.0 | 381.7 | # | translation |  |
|  | PAAG_08702 | seryl-tRNA synthetase | 17.0 | 663.3 | # | translation |  |
| **PROTEIN FATE** | | | | | | |  |
|  | PAAG_07802 | proteasome component PRE6 | 8.8 | 1141.1 | 2.80 | protein degradation |  |
|  | PAAG_07467 | dipeptidyl-peptidase | 19.0 | 286.8 | 1.60 | protein degradation |  |
|  | PAAG_08141 | proteasome subunit alpha type-4 | 8.7 | 617.9 | 1.37 | protein degradation |  |
|  | PAAG_04291 | nucleoside diphosphate kinase | 5.5 | 14408.8 | 0.79 | modification by phosphorylation. dephosphorylation. autophosphorylation |  |
|  | PAAG_06255 | mitochondrial co-chaperone GrpE | 5.5 | 1648.9 | 0.75 | protein folding |  |
|  | PAAG_03334 | peptidyl-prolyl cis-trans isomerase D | 14.2 | 6691.7 | 0.69 | protein folding |  |
|  | PAAG_07080 | ubiquitin | 5.0 | 11204.1 | 0.61 | modification by ubiquitination, deubiquitination |  |
|  | PAAG_06536 | ubiquitin | 5.3 | 11222.2 | 0.61 | modification by ubiquitination, deubiquitination |  |
|  | PAAG_00238 | ubiquitin | 7.2 | 11202.3 | 0.59 | modification by ubiquitination, deubiquitination |  |
|  | PAAG_07500 | xaa-Pro aminopeptidase | 8.3 | 347.9 | # | proteolysis |  |
|  | PAAG_07319 | xaa-Pro aminopeptidase | 13.0 | 295.5 | # | proteolysis |  |
|  | PAAG_03719 | Thimet oligopeptidase | 12.0 | 151.4 | # | proteolysis |  |
|  | PAAG_01472 | ubiquitin-conjugating enzyme | 6.0 | 192.9 | # | protein degradation |  |
|  | PAAG_00852 | proteasome component C1 | 7.3 | 201.5 | # | proteasomal degradation (ubiquitin/proteasomal pathway) |  |
|  | PAAG_00868 | proteasome component PRE4 | 4.0 | 457.1 | # | proteasomal degradation (ubiquitin/proteasomal pathway) |  |
|  | PAAG_03536 | proteasome component PRE5 | 6.3 | 671.1 | # | proteasomal degradation (ubiquitin/proteasomal pathway) |  |
|  | PAAG_02720 | proteasome-activating nucleotidase | 14.0 | 173.4 | # | proteasomal degradation (ubiquitin/proteasomal pathway) |  |
|  | PAAG_03279 | aminopeptidase | 21.0 | 238.6 | # | proteolysis |  |
|  | PAAG_00664 | aspartyl aminopeptidase | 12.3 | 1196.0 | # | protein/peptide degradation |  |
|  | PAAG_03464 | bleomycin hydrolase | 10.0 | 169.2 | # | proteolysis |  |
|  | PAAG_05583 | cysteine protease PalB | 17.0 | 139.8 | # | proteolysis |  |
|  | PAAG_00768 | peptidase family protein | 6.0 | 213.7 | # | proteolysis |  |
|  | PAAG_05417 | mitochondrial-processing peptidase subunit beta | 12.5 | 462.3 | # | proteolysis |  |
|  | PAAG_00739 | peptidyl-prolyl cis-trans isomerase B | 4.3 | 485.6 | # | protein folding |  |
|  | PAAG_02155 | peroxisomal targeting signal 2 receptor (PTS2) | 10.0 | 217.9 | # | protein fate |  |
|  | PAAG_04555 | sarcosine oxidase | 10.0 | 166.5 | # | protein modification |  |
|  | PAAG_00472 | serine/threonine-protein phosphatase | 7.0 | 170.4 | # | protein dephosphorilation |  |
|  | PAAG_03573 | vacuolar protein sorting-associated protein | 9.0 | 181.4 | # | protein fate |  |
| **BINDING** | | | | | | |  |
|  | PAAG_07038 | APAF1-interacting protein | 4.5 | 292.6 | # | metal binding |  |
|  | PAAG_08026 | MYB DNA-binding domain-containing protein | 6.0 | 196.2 | # | DNA binding |  |
|  | PAAG_07753 | RNA-binding La domain-containing protein | 9.0 | 173.7 | # | RNA binding |  |
| **TRANSPORT** | | | | | | |  |
|  | PAAG_05960 | NIPSNAP family protein | 11.2 | 906.6 | 1.20 | vesicular transport (Golgi network. etc.) |  |
|  | PAAG_03577 | ABC drug exporter AtrF | 21.0 | 155.8 | # | drug/ toxin transport |  |
|  | PAAG_05425 | Golgi membrane protein (Coy1) | 21.0 | 354.9 | # | Golgi vesicle transport |  |
|  | PAAG_04571 | nascent polypeptide-associated complex subunit alpha | 3.7 | 621.3 | # | protein transport |  |
|  | PAAG_08082 | plasma membrane ATPase | 11.0 | 179.2 | # | hydrogen ion transport |  |
| **SIGNAL TRANSDUCTION** | | | | | | |  |
|  | PAAG_02466 | ran-specific GTPase-activating protein | 5.5 | 4064.5 | 0.63 | [signal transduction_regulation of chromatin silencing](http://www.ebi.ac.uk/QuickGO/GTerm?id=GO:0031938) |  |
|  | PAAG_02973 | diploid state maintenance protein chpA | 7.0 | 409.8 | # | cellular signalling |  |
|  | PAAG_04261 | signal recognition particle 54 kDa protein | 11.0 | 188.8 | # | GTP catabolic process |  |
|  | PAAG_02377 | rho GDP-dissociation inhibitor | 4.0 | 841.3 | # | regulator of G-protein signaling |  |
| **CELL RESCUE, DEFENSE and VIRULENCE** | | | | | | |  |
|  | PAAG_07020 | thioredoxin reductase | 6.5 | 587.8 | 3.78 | cell redox homeostase |  |
|  | PAAG_04851 | osmotic growth protein | 16.8 | 1114.5 | 1.23 | response to anoxia |  |
|  | PAAG_02364 | thioredoxin | 3.7 | 15346.5 | 1.07 | cell redox homeostase |  |
|  | PAAG_05142 | 10 kDa heat shock protein mitochondrial | 7.0 | 29047.1 | 0.97 | stress response |  |
|  | PAAG_07750 | heat shock protein Hsp88 | 30.3 | 7178.0 | 0.91 | stress response |  |
|  | PAAG_08260 | Hsp90 co-chaperone Cdc37 | 11.0 | 1034.7 | 0.91 | stress response |  |
|  | PAAG_07775 | heat shock protein SSB1 | 15.0 | 1071.7 | 0.71 | stress response |  |
|  | PAAG_01262 | hsp70-like protein | 22.5 | 3529.6 | 0.65 | stress response |  |
|  | PAAG_02926 | superoxide dismutase | 4.3 | 698.6 | # | detoxification |  |
|  | PAAG_03502 | cytochrome c peroxidase | 6.0 | 192.5 | # | oxidative stress response |  |
|  | PAAG_00566 | aflatoxin B1 aldehyde reductase member 2 | 12.0 | 493.8 | # | detoxification |  |
|  | PAAG_06606 | cyanate hydratase | 4.0 | 482.3 | # | stress response/ cyanate metabolic process |  |
|  | PAAG_06947 | gamma-glutamyltranspeptidase | 12.0 | 206.4 | # | cell redox homeostase |  |
|  | PAAG_02548 | hydroxyacylglutathione hydrolase | 4.0 | 293.4 | # | glutathione biosynthetic process/ stress response |  |
|  | PAAG_08277 | nitroreductase family protein | 4.3 | 1157.8 | # | detoxification |  |
| **CELL GROWTH/ MORPHOGENESIS** | | | | | | |  |
|  | PAAG_00997 | actin-interacting protein | 12.0 | 192.4 | # | actin depolymerization |  |
|  | PAAG_00875 | ARP2/3 actin-organizing complex subunit Sop2 | 6.0 | 360.9 | # | actin filament organization |  |
|  | PAAG_03624 | Arp2/3 complex subunit Arc16 | 5.0 | 537.3 | # | actin filament organization |  |
|  | PAAG_04602 | mannosyl-oligosaccharide glucosidase | 10.0 | 175.9 | # | cell wall biogenesis |  |
|  | PAAG_02186 | nuclear segregation protein Bfr1 | 10.0 | 336.7 | # | meiosis |  |
|  | PAAG_01931 | phosphoacetylglucosamine mutase | 9.3 | 326.2 | # | cell wall processing |  |
| **MISCELLANEOUS** | | | | | | |  |
|  | PAAG_08247 | calmodulin | 4.5 | 1846.5 | 0.69 | control of enzymes. ion channels and other proteins by Ca^2+^ |  |
|  | PAAG_02139 | methyltransferase family | 5.0 | 190.5 | # | methyltransferase |  |
|  | PAAG_01302 | phosphorylase family protein | 6.5 | 664.6 | # | phosphoprotein |  |
|  | PAAG_03233 | [oxidoreductase](http://www.ncbi.nlm.nih.gov/blast/Blast.cgi#alnHdr_240281746) | 7.0 | 156.4 | # | oxidation-reduction process |  |
|  | PAAG_00712 | [N-acetyltransferase](http://www.ncbi.nlm.nih.gov/blast/Blast.cgi#alnHdr_226292615) | 6.0 | 214.0 | # | acetyltransferase |  |
|  | PAAG_06955 | thiol methyltransferase | 5.7 | 702.7 | # | thiol methyltransferase activity |  |
| **UNCLASSIFIED** | | | | | | | |
|  | PAAG_06515 | DUF833 domain-containing protein | 6.6 | 370.4 | 3.46 | - |  |
|  | PAAG_00340 | conserved hypothetical protein | 8.3 | 10679.8 | 1.96 | - |  |
|  | PAAG_06083 | dienelactone hydrolase family protein | 4.2 | 3409.2 | 1.40 | - |  |
|  | PAAG_00297 | conserved hypothetical protein | 7.3 | 1293.1 | 1.37 | - |  |
|  | PAAG_04478 | dienelactone hydrolase family protein | 5.5 | 502.3 | # | - |  |
|  | PAAG_02665 | DlpA domain-containing protein | 5.0 | 433.5 | # | - |  |
|  | PAAG_01399 | NAD dependent epimerase/dehydratase family protein | 7.0 | 259.8 | # | - |  |
|  | PAAG_07821 | NAD dependent epimerase/dehydratase family protein | 9.0 | 167.7 | # | - |  |
|  | PAAG_04220 | predicted protein | 3.0 | 284.6 | # | - |  |
|  | PAAG_09108 | RPEL repeat protein | 2.0 | 382.7 | # | - |  |
|  | PAAG_04282 | UBX domain-containing protein | 10.0 | 246.5 | # | - |  |
|  | PAAG_01127 | WD repeat domain 5B | 8.0 | 170.7 | # | - |  |
|  | PAAG_00335 | [MYG1 protein](http://www.ncbi.nlm.nih.gov/blast/Blast.cgi#alnHdr_261198959) | 8.0 | 330.3 | # | - |  |
|  | PAAG_00681 | predicted protein | 6.0 | 224.1 | # | - |  |
|  | PAAG_03778 | predicted protein | 2.0 | 348.3 | # | - |  |
|  | PAAG_01254 | predicted protein | 3.0 | 245.1 | # | - |  |
|  | PAAG_03726 | predicted protein | 6.0 | 234.6 | # | - |  |
|  | PAAG_04975 | predicted protein | 5.0 | 163.3 | # | - |  |
|  | PAAG_07581 | predicted protein | 6.0 | 152.7 | # | - |  |
|  | PAAG_04812 | predicted protein | 1.0 | 316.7 | # | - |  |
|  | PAAG_08005 | hypothetical protein | 3.0 | 901.4 | # | - |  |
|  | PAAG_02985 | hypothetical protein | 3.0 | 942.3 | # | - |  |
|  | PAAG_05933 | hypothetical protein | 3.0 | 194.1 | # | - |  |
|  | PAAG_05550 | hypothetical protein | 4.0 | 855.3 | # | - |  |
|  | PAAG_03239 | hypothetical protein | 1.0 | 291.3 | # | - |  |
|  | PAAG_01455 | hypothetical protein | 1.0 | 344.0 | # | - |  |
|  | PAAG_02242 | hypothetical protein | 6.0 | 309.4 | # | - |  |
|  | PAAG_03092 | hypothetical protein | 10.7 | 609.9 | # | - |  |
|  | PAAG_01863 | hypothetical protein | 3.5 | 811.9 | # | - |  |
|  | PAAG_06462 | conserved hypothetical protein | 2.5 | 925.7 | # | - |  |
|  | PAAG_07409 | conserved hypothetical protein | 3.0 | 239.9 | # | - |  |
|  | PAAG_02019 | conserved hypothetical protein | 3.3 | 6334.5 | # | - |  |
|  | PAAG_06523 | conserved hypothetical protein | 4.0 | 275.3 | # | - |  |
|  | PAAG_05856 | conserved hypothetical protein | 1.3 | 10414.5 | # | - |  |
|  | PAAG_05222 | conserved hypothetical protein | 3.0 | 281.5 | # | - |  |
|  | PAAG_05692 | conserved hypothetical protein | 7.0 | 185.8 | # | - |  |
|  | PAAG_05874 | conserved hypothetical protein | 5.5 | 302.5 | # | - |  |
|  | PAAG_04274 | conserved hypothetical protein | 2.5 | 210.6 | # | - |  |
|  | PAAG_01592 | conserved hypothetical protein | 4.0 | 217.1 | # | - |  |
|  | PAAG_07184 | conserved hypothetical protein | 8.0 | 254.8 | # | - |  |
|  | PAAG_07886 | conserved hypothetical protein | 5.0 | 200.3 | # | - |  |
|  | PAAG_07989 | conserved hypothetical protein | 6.3 | 218.9 | # | - |  |
|  | PAAG_01075 | conserved hypothetical protein | 10.0 | 169.9 | # | - |  |
|  | PAAG_05277 | conserved hypothetical protein | 18.0 | 139.5 | # | - |  |
|  | PAAG_07386 | conserved hypothetical protein | 2.0 | 661.9 | # | - |  |
|  | PAAG_05766 | conserved hypothetical protein | 17.0 | 171.5 | # | - |  |
|  | PAAG_03368 | conserved hypothetical protein | 16.0 | 141.3 | # | - |  |

^a^ Identification of differentially regulated proteins from *Paracoccidioides* genome database (<http://www.broadinstitute.org/annotation/genome/paracoccidioides_brasiliensis/MultiHome.html>) using the ProteinLynx Global Server (PLGS) version 3.0 (Waters Corporation. Manchester. UK);

^b^ Proteins annotation from *Paracoccidioides* genome database or by homology from NCBI database (<http://www.ncbi.nlm.nih.gov/>);

^c^  Average of matching peptides and score for each protein obtained from MS data using the ProteinLynx Global Server (PLGS);

^d^ Protein expression profiles in log2 (fold change) obtained from ProteinLynx Global Server (PLGS) analysis normalized with internal standard.

^e^ Biological process of differentially expressed proteins from MIPS

(<http://pedant.helmholtz-muenchen.de/pedant3htmlview/pedant3view?Method=analysis&Db=p3_r48325_Par_brasi_Pb01> ) and Uniprot database (<http://www.uniprot.org/>).

#: identified only in carbon starvation condition.
