# Supplementary material for: Transcriptional and Proteomic Responses to Carbon Starvation in Paracoccidioides
Source: PLoS Negl Trop Dis. 2014 May 8;8(5):e2855. doi: 10.1371/journal.pntd.0002855 (PMC4014450; doi:10.1371/journal.pntd.0002855)
Supplement: Table S6 — Down-regulated proteins of Paracoccidioides ( Pb 01) yeast cells under carbon starvation detected using NanoUPLC-MSE. (DOC) [file pntd.0002855.s017.doc]

**Table S6. Down-regulated proteins of *Paracoccidioides* (*Pb*01) yeast cells under carbon starvation detected using NanoUPLC-MS^E^**.

|  | **ID^a^** | **Annotation^b^** | **Peptides AVG^c^** | **Score AVG^c^** | **Fold change^d^** | **Biological process^e^** |
| --- | --- | --- | --- | --- | --- | --- |
| **METABOLISM** | | | | | | |
| **Amino acid metabolism** | | | | | | |
|  | PAAG_04701 | cystathionine gamma-lyase | 7.2 | 276.0 | -1.86 | cysteine biosynthesis |
|  | PAAG_05005 | anthranilate synthase component 1 | 10.5 | 481.8 | -1.11 | tryptophan biosynthesis |
|  | PAAG_02603 | aspartate aminotransferase | 11.8 | 734.0 | -0.88 | aspartate biosynthesis |
|  | PAAG_07760 | threonine synthase | 12.5 | 343.2 | -0.74 | threonine biosynthesis |
|  | PAAG_03569 | 1.2-dihydroxy-3-keto-5-methylthiopentene dioxygenase | 7.0 | 465.5 | * | methionine biosynthesis |
|  | PAAG_05328 | 3-isopropylmalate dehydrogenase A | 13.0 | 531.5 | * | leucine biosynthesis |
|  | PAAG_07605 | acetolactate synthase small subunit | 5.7 | 339.3 | * | L-isoleucine biosynthesis |
|  | PAAG_03043 | adenylyl-sulfate kinase | 4.3 | 513.2 | * | methionine biosynthesis |
|  | PAAG_07563 | asparagine synthetase | 9.5 | 321.9 | * | asparagine biosynthesis |
|  | PAAG_05198 | chorismate mutase | 10.0 | 690.9 | * | cysteine biosynthesis and aromatic groups |
|  | PAAG_07813 | cysteine synthase | 11.0 | 1040.8 | * | cysteine biosynthesis |
|  | PAAG_07089 | homocitrate synthase | 9.7 | 358.6 | * | lysine biosynthesis |
|  | PAAG_04348 | homoserine kinase | 4.0 | 326.8 | * | threonine biosynthesis |
|  | PAAG_07102 | pentafunctional AROM polypeptide | 31.0 | 376.9 | * | aromatic group biosynthesis |
|  | PAAG_05929 | sulfate adenylyltransferase | 18.3 | 2080.4 | * | cysteine and methionine biosynthesis |
| **C-compound and carbohydrate metabolism** | | | | | | |
|  | PAAG_00850 | glucosamine-fructose-6-phosphate aminotransferase | 12.8 | 1553.4 | -1.50 | C-compound and carbohydrate metabolism |
|  | PAAG_00545 | glycogen phosphorylase | 27.3 | 1583.0 | -1.47 | Carbohydrate metabolism |
|  | PAAG_07804 | D-lactate dehydrogenase | 11.0 | 522.2 | -1.43 | C-compound process/ lactate oxidation process |
|  | PAAG_08295 | pyruvate dehydrogenase E1 component subunit alpha | 9.8 | 609.4 | -1.21 | C-compound and carbohydrate metabolism/ Acetyl-CoA from pyruvate |
|  | PAAG_01534 | pyruvate dehydrogenase E1 component subunit beta | 10.2 | 4412.5 | -1.08 | C-compound and carbohydrate metabolism/ Acetyl-CoA from pyruvate |
|  | PAAG_00666 | pyruvate dehydrogenase complex component Pdx1 | 6.0 | 1198.2 | -0.76 | C-compound process/ pyruvate metabolic process |
|  | PAAG_08174 | mannose-1-phosphate guanyltransferase subunit beta-A | 10.0 | 432.5 | -0.65 | C-compound and carbohydrate metabolism |
|  | PAAG_02975 | 2.4-dihydroxyhept-2-ene-1.7-dioic acid aldolase | 5.0 | 464.5 | * | [phenylacetate catabolic process](http://www.ebi.ac.uk/QuickGO/GTerm?id=GO:0010124) |
|  | PAAG_02769 | pyruvate dehydrogenase protein X component | 12.0 | 589.1 | * | carbohydrate metabolism |
|  | PAAG_03774 | S-(hydroxymethyl)glutathione dehydrogenase | 12.0 | 421.3 | * | C-compound and carbohydrate metabolism/ metane metabolism |
|  | PAAG_00799 | uroporphyrinogen decarboxylase | 9.0 | 252.9 | * | porphyrin biosynthesis |
| **Lipid, fatty acid and isoprenoid metabolism** | | | | | | |
|  | PAAG_05150 | ATP-citrate synthase subunit 1 | 18.8 | 540.2 | -0.87 | lipid metabolism_acetyl CoA biosynthesis |
|  | PAAG_05093 | succinyl-CoA: 3-ketoacid-coenzyme A transferase subunit B | 15.3 | 3325.1 | -0.59 | [lipopolysaccharide biosynthesis](http://www.uniprot.org/keywords/KW-0448) |
|  | PAAG_02211 | [GDSL Lipase/Acylhydrolase](http://www.ncbi.nlm.nih.gov/blast/Blast.cgi#alnHdr_261190038) | 6.0 | 322.3 | * | lipid metabolism |
|  | PAAG_07013 | enoyl-CoA hydratase/carnithine racemase | 5.0 | 304.1 | * | lipid. fatty acid and isoprenoid metabolism |
|  | PAAG_01525 | fatty acid synthase subunit alpha reductase | 42.7 | 340.8 | * | lipid and fatty acid biosynthesis |
|  | PAAG_01524 | fatty acid synthase subunit beta dehydratase | 44.3 | 1308.7 | * | lipid and fatty acid biosynthesis |
|  | PAAG_00869 | fumarylacetoacetate hydrolase domain-containing protein | 5.0 | 358.8 | * | lipid. fatty acid and isoprenoid metabolism |
|  | PAAG_06215 | hydroxymethylglutaryl-CoA lyase | 5.5 | 273.6 | * | lipid. fatty acid and isoprenoid metabolism |
| **Purin nucleotide/ nucleoside/ nucleobase metabolism** | | | | | | |
|  | PAAG_06906 | adenine phosphoribosyltransferase | 5.3 | 922.0 | * | purin nucleotide/nucleoside/nucleobase metabolism |
|  | PAAG_07529 | orotidine 5'-phosphate decarboxylase | 2.7 | 546.4 | * | pyrimidine biosynthesis |
|  | PAAG_01437 | uricase | 6.5 | 435.5 | * | purine metabolism |
| **Secundary metabolism** | | | | | | |
|  | PAAG_00047 | Siroheme synthase | 6.5 | 358.8 | * | cobalamin (vitamin B12) biosynthesis |
|  | PAAG_01519 | inositol monophosphatase | 4.0 | 319.9 | * | secondary metabolism |
| **ENERGY** | | | | | | |
| **Glycolysis and gluconeogenesis** | | | | | | |
|  | PAAG_02869 | phosphoglycerate kinase | 16.8 | 1827.4 | -1.07 | glycolysis |
|  | PAAG_00726 | pyruvate carboxylase | 28.3 | 463.5 | -0.81 | pyruvate metabolism |
|  | PAAG_01015 | hexokinase | 7.0 | 373.0 | * | glycolysis |
| **Glyoxylate cycle** | | | | | | |
|  | PAAG_04542 | malate synthase | 15.5 | 1473.1 | -0.69 | glyoxylate cycle |
| **Tricarboxylic-acid pathway** | | | | | | |
|  | PAAG_01463 | succinyl-CoA ligase subunit beta | 17.4 | 1934.2 | -1.92 | TCA cycle |
|  | PAAG_00417 | succinyl-CoA ligase subunit alpha | 10.2 | 2036.3 | -1.21 | TCA cycle |
|  | PAAG_07729 | isocitrate dehydrogenase subunit 2 | 12.3 | 2284.2 | -1.00 | TCA cycle |
|  | PAAG_00856 | isocitrate dehydrogenase subunit 1 | 11.3 | 1307.7 | -0.95 | TCA cycle |
| **Electron transport and membrane-associated energy conservation** | | | | | | |
|  | PAAG_00953 | NADH-cytochrome b5 reductase | 10.5 | 703.4 | -0.59 | electron transport |
|  | PAAG_07285 | vacuolar ATP synthase catalytic subunit A | 11.0 | 267.9 | * | [ATP hydrolysis coupled proton transport](http://www.ebi.ac.uk/QuickGO/GTerm?id=GO:0015991) |
|  | PAAG_06288 | vacuolar ATP synthase subunit B | 13.0 | 339.2 | * | [ATP hydrolysis coupled proton transport](http://www.ebi.ac.uk/QuickGO/GTerm?id=GO:0015991) |
|  | PAAG_06155 | vacuolar ATP synthase subunit E | 6.0 | 1306.2 | * | [ATP hydrolysis coupled proton transport](http://www.ebi.ac.uk/QuickGO/GTerm?id=GO:0015991) |
| **Pentose Phosphate pathway** | | | | | | |
|  | PAAG_00633 | glucose-6-phosphate 1-dehydrogenase | 10.0 | 254.3 | * | glucose metabolism |
| **CELL CYCLE and DNA PROCESSING** | | | | | | |
|  | PAAG_04949 | UV excision repair protein Rad23 | 6.3 | 1289.8 | -0.82 | DNA repair |
|  | PAAG_06751 | DNA damage checkpoint protein rad24 | 14.8 | 13494.5 | -0.62 | cell cycle |
|  | PAAG_06751 | DNA damage checkpoint protein rad24 | 14.8 | 13494.5 | -0.62 | cell cycle |
|  | PAAG_07175 | vacuolar sorting-associated protein | 4.8 | 369.6 | -0.59 | protein sorting in cell division |
|  | PAAG_03834 | vacuolar sorting-associated protein | 6.0 | 382.4 | * | protein sorting in cell division |
|  | PAAG_07773 | cyclin-dependent kinases regulatory subunit | 4.0 | 451.2 | * | mitotic cell cycle and cell cycle control |
|  | PAAG_00106 | histone acetyltransferase type B catalytic subunit | 8.0 | 397.9 | * | DNA repair |
|  | PAAG_02055 | histone chaperone asf1 | 3.0 | 938.2 | * | DNA replication |
|  | PAAG_01943 | spindle pole body component alp6 | 14.0 | 251.2 | * | cell cycle |
| **TRANSCRIPTION** | | | | | | |
|  | PAAG_07957 | pre-mRNA-splicing factor srp1 | 7.0 | 689.5 | -1.50 | splicing |
|  | PAAG_06891 | mRNA binding post-transcriptional regulator (Csx1) | 6.5 | 250.9 | -1.15 | mRNA synthesis |
|  | PAAG_01695 | [arsenite resistance protein Ars2](http://www.ncbi.nlm.nih.gov/blast/Blast.cgi#alnHdr_327349561) | 14.7 | 308.4 | * | transcription |
|  | PAAG_04161 | [transcription factor](http://www.ncbi.nlm.nih.gov/blast/Blast.cgi#alnHdr_239612135) | 23.0 | 257.9 | * | transcription control |
|  | PAAG_08234 | [transcription factor RfeF](http://www.ncbi.nlm.nih.gov/blast/Blast.cgi#alnHdr_325094709) | 4.0 | 359.3 | * | transcription control |
|  | PAAG_01733 | 28 kDa ribonucleoprotein | 7.3 | 1255.6 | * | transcriptional control |
|  | PAAG_00101 | small nuclear ribonucleoprotein | 3.0 | 447.2 | * | splicing |
|  | PAAG_01630 | small nuclear ribonucleoprotein LSM2 | 3.0 | 675.1 | * | splicing |
|  | PAAG_02329 | U2 small nuclear ribonucleoprotein A | 4.3 | 453.0 | * | splicing |
|  | PAAG_07983 | ribonuclease H | 9.0 | 249.3 | * | RNA processing |
|  | PAAG_06966 | RNA methyltransferase | 14.0 | 259.6 | * | RNA processing |
| **TRANSLATION** | | | | | | |
|  | PAAG_02024 | elongation factor 1-alpha | 16.7 | 6102.2 | -1.04 | translation |
|  | PAAG_04690 | 40S ribosomal protein S15 | 3.0 | 5894.3 | -1.04 | ribosome biogenesis |
|  | PAAG_02921 | elongation factor Tu | 15.3 | 1074.9 | -0.85 | translation |
|  | PAAG_00801 | 60S acidic ribosomal protein P0 lyase | 6.0 | 499.9 | -0.74 | ribosome biogenesis |
|  | PAAG_02634 | ribosomal protein S6 | 8.0 | 1258.7 | -0.61 | ribosome biogenesis |
|  | PAAG_00765 | [60S ribosomal protein L36](http://www.ncbi.nlm.nih.gov/blast/Blast.cgi#alnHdr_295672720) | 6.0 | 1386.8 | * | ribosome biogenesis |
|  | PAAG_00689 | ATP-dependent RNA helicase eIF4A | 9.7 | 585.5 | * | protein biosynthesis |
|  | PAAG_07283 | ATP-dependent RNA helicase FAL1 | 11.0 | 261.6 | * | ribossome biogenesis |
|  | PAAG_06140 | eukaryotic translation initiation factor 1A | 5.0 | 438.9 | * | translation |
|  | PAAG_00747 | eukaryotic translation initiation factor 2 subunit gamma | 8.7 | 408.6 | * | translation |
|  | PAAG_01330 | eukaryotic translation initiation factor 3 RNA-binding subunit | 4.0 | 334.8 | * | translation |
|  | PAAG_00815 | eukaryotic translation initiation factor 3 subunit A | 16.0 | 258.3 | * | translation |
|  | PAAG_02837 | eukaryotic translation initiation factor 3 subunit H | 8.5 | 352.0 | * | translation |
|  | PAAG_04958 | eukaryotic translation initiation factor 6 | 6.5 | 852.6 | * | translation |
|  | PAAG_02071 | glutamyl-tRNA synthetase | 14.5 | 521.8 | * | aminoacyl-tRNA-synthetases |
|  | PAAG_01786 | phenylalanyl-tRNA synthetase beta chain | 13.7 | 452.2 | * | translation |
|  | PAAG_08025 | tRNA (uracil-5-)-methyltransferase TRM9 | 6.0 | 376.5 | * | tRNA modification |
| **PROTEIN FATE** | | | | | | |
|  | PAAG_01778 | peptidyl-prolyl cis-trans isomerase H | 4.8 | 444.3 | -1.62 | protein folding and stabilization |
|  | PAAG_07339 | S-phase kinase-associated protein 1A | 6.0 | 1591.6 | -1.40 | histone H2A monoubiquitination |
|  | PAAG_06068 | T-complex protein 1 subunit beta | 13.5 | 310.7 | -1.37 | protein folding |
|  | PAAG_01727 | T-complex protein 1 subunit delta | 16.5 | 382.1 | -1.05 | protein folding |
|  | PAAG_02497 | [WD repeat domain 5B](http://www.ncbi.nlm.nih.gov/blast/Blast.cgi#alnHdr_225684485) | 13.0 | 331.0 | * | [Ubl conjugation pathway](http://www.uniprot.org/keywords/KW-0833) |
|  | PAAG_01926 | [26S protease regulatory subunit 6A](http://www.ncbi.nlm.nih.gov/blast/Blast.cgi#alnHdr_226288798) | 7.0 | 356.9 | * | [ubiquitin-dependent protein proteolysis](http://www.ebi.ac.uk/QuickGO/GTerm?id=GO:0006511) |
|  | PAAG_05943 | 26S proteasome non-ATPase regulatory subunit 12 | 9.0 | 429.8 | * | assembly of proteasome |
|  | PAAG_01706 | 26S proteasome regulatory subunit RPN10 | 4.0 | 707.9 | * | protein/peptide degradation |
|  | PAAG_08020 | 26S proteasome regulatory subunit rpn-8 | 7.0 | 410.5 | * | protein/peptide degradation |
|  | PAAG_07037 | calnexin | 10.0 | 370.5 | * | protein folding and stabilization |
|  | PAAG_01962 | proteasome 26S subunit | 5.0 | 949.5 | * | protein/peptide degradation |
|  | PAAG_08184 | T-complex protein 1 epsilon subunit | 10.0 | 374.2 | * | protein folding |
|  | PAAG_07165 | T-complex protein 1 subunit gamma | 16.5 | 294.4 | * | protein folding |
|  | PAAG_01588 | SNARE Ykt6 | 7.0 | 685.2 | * | protein targeting, sorting and translocation |
|  | PAAG_04327 | ubiquitin carboxyl-terminal hydrolase | 17.0 | 217.9 | * | protein deubiquitination |
|  | PAAG_03932 | [ubiquitin-activating enzyme E1 Y](http://www.ncbi.nlm.nih.gov/blast/Blast.cgi#alnHdr_225680103) | 16.3 | 374.2 | * | protein ubiquitination |
| **BINDING** | | | | | | |
|  | PAAG_04391 | progesterone binding protein | 3.0 | 970.3 | -1.34 | metal binding |
|  | PAAG_03941 | G4 quadruplex nucleic acid binding protein | 10.7 | 471.5 | * | RNA binding |
| **TRANSPORT** | | | | | | |
|  | PAAG_04651 | GTP-binding nuclear protein GSP1/Ran | 5.8 | 1134.8 | -1.59 | transport |
|  | PAAG_08487 | [MIT family metal ion transporter](http://www.ncbi.nlm.nih.gov/blast/Blast.cgi#alnHdr_225684145) | 9.0 | 272.1 | * | ion transport (cobalt) |
|  | PAAG_05135 | [tetratricopeptide repeat protein 1](http://www.ncbi.nlm.nih.gov/blast/Blast.cgi#alnHdr_327352245) | 7.0 | 351.0 | * | potassium transport |
|  | PAAG_04904 | ATP-binding cassette sub-family F member 2 | 11.5 | 421.2 | * | ABC transport |
|  | PAAG_03644 | mitochondrial import receptor subunit tom-40 | 9.0 | 351.5 | * | protein transport |
|  | PAAG_02306 | vacuolar H+\/Ca2+ exchanger | 4.0 | 253.8 | * | ion transport |
|  | PAAG_07900 | phosphatidylinositol-phosphatidylcholine transfer protein (SEC14) | 9.0 | 404.3 | * | protein transport |
| **SIGNAL TRANSDUCTION** | | | | | | |
|  | PAAG_07634 | small GTPase RhoA | 5.0 | 2087.7 | -1.36 | signal transduction/ cell migration |
|  | PAAG_06344 | rab GDP-dissociation inhibitor | 15.3 | 2416.2 | -0.87 | signal transduction |
|  | PAAG_08028 | GTP-binding protein ypt1 | 4.0 | 281.1 | * | protein transport/ signal transduction process |
|  | PAAG_02458 | GTP-binding protein ypt7 | 6.0 | 305.1 | * | protein transport/ signal transduction process |
|  | PAAG_08992 | type 2A phosphatase activator tip41 | 4.0 | 255.5 | * | signal transduction |
| **CELL RESCUE, DEFENSE AND VIRULENCE** | | | | | | |
|  | PAAG_05679 | heat shock protein | 16.4 | 2679.1 | -3.45 | stress response_protein folding |
|  | PAAG_04164 | superoxide dismutase | 4.2 | 2084.0 | -1.02 | oxidative stress response |
|  | PAAG_00871 | 30 kDa heat shock protein | 9.5 | 2657.6 | -0.59 | stress response_protein folding |
|  | PAAG_05392 | betaine aldehyde dehydrogenase | 12.0 | 2316.8 | -0.58 | oxireductase |
|  | PAAG_07990 | tetracycline transporter | 4.0 | 248.3 | * | antibiotic resistence |
|  | PAAG_01465 | carbonic anhydrase | 6.3 | 21001.6 | * | stress oxidative response/ carbon utilization |
|  | PAAG_03216 | mitochondrial peroxiredoxin PRX1 | 10.7 | 14525.5 | * | oxidative stress response |
| **CELL GROWTH/ MORPHOGENESIS** | | | | | | |
|  | PAAG_07234 | NAP family protein | 8.0 | 388.3 | -1.26 | cytoskeleton organization |
|  | PAAG_03031 | tubulin beta chain | 8.2 | 362.9 | -1.17 | cell morphogenesis |
|  | PAAG_01647 | tubulin alpha-1 chain | 12.2 | 2901.5 | -1.04 | cell morphogenesis |
|  | PAAG_02396 | actin | 11.3 | 853.3 | -0.74 | cell morphogenesis |
|  | PAAG_01986 | nucleosome binding protein | 2.3 | 4835.6 | -0.71 | budding cell growth |
|  | PAAG_06370 | sphingolipid long chain base-responsive protein LSP1 | 13.0 | 315.1 | * | endocytosis |
| **MISCELLANEOUS** | | | | | | |
|  | PAAG_04908 | NAD binding Rossmann fold oxidoreductase | 6.0 | 268.9 | * | oxidoreductase |
|  | PAAG_02354 | serine 3-dehydrogenase | 9.0 | 2405.8 | * | [serine 3-dehydrogenase activity](http://www.ebi.ac.uk/QuickGO/GTerm?id=GO:0031132) |
| **UNCLASSIFIED** | | | | | | |
|  | PAAG_03309 | suaprga1 | 5.8 | 1963.0 | -1.67 | - |
|  | PAAG_03152 | CobW domain-containing protein | 7.6 | 632.0 | -1.31 | - |
|  | PAAG_00503 | HAD-superfamily hydrolase | 6.8 | 820.8 | -0.78 | - |
|  | PAAG_08370 | conserved hypothetical protein | 10.3 | 377.7 | -0.75 | - |
|  | PAAG_07772 | conserved hypothetical protein | 6.5 | 3329.7 | -0.69 | - |
|  | PAAG_03701 | BAR domain-containing protein | 8.2 | 637.9 | -0.66 | - |
|  | PAAG_03649 | WD repeat-containing protein | 12.0 | 326.1 | -0.66 | - |
|  | PAAG_05087 | RNP domain-containing protein | 8.0 | 523.2 | * | - |
|  | PAAG_05181 | conserved leucine-rich repeat protein | 6.0 | 221.4 | * | - |
|  | PAAG_03890 | DUF866 domain-containing protein | 8.0 | 306.2 | * | - |
|  | PAAG_08103 | EF hand domain-containing protein | 25.0 | 368.9 | * | - |
|  | PAAG_02567 | EF-hand superfamily Ca2+-modulated protein | 11.0 | 457.4 | * | - |
|  | PAAG_01160 | DNL zinc finger domain-containing protein | 5.0 | 256.9 | * | - |
|  | PAAG_05037 | HHE domain-containing protein | 4.0 | 334.5 | * | - |
|  | PAAG_01097 | Poly(rC)-binding protein | 10.0 | 350.4 | * | - |
|  | PAAG_02382 | quinone oxidoreductase | 11.7 | 533.0 | * | - |
|  | PAAG_04793 | [LEA domain-containing protein](http://www.ncbi.nlm.nih.gov/blast/Blast.cgi#alnHdr_225684485) | 11.3 | 2586.5 | * | - |
|  | PAAG_03611 | [extracellular serine-rich protein](http://www.ncbi.nlm.nih.gov/blast/Blast.cgi#alnHdr_327354056) | 7.0 | 216.8 | * | - |
|  | PAAG_06914 | conserved hypothetical protein | 5.0 | 269.0 | * | - |
|  | PAAG_01504 | conserved hypothetical protein | 3.0 | 259.6 | * | - |
|  | PAAG_05262 | conserved hypothetical protein | 2.0 | 225.3 | * | - |
|  | PAAG_05639 | conserved hypothetical protein | 3.0 | 267.5 | * | - |
|  | PAAG_04730 | conserved hypothetical protein | 5.0 | 282.5 | * | - |
|  | PAAG_05623 | conserved hypothetical protein | 13.0 | 242.9 | * | - |
|  | PAAG_04989 | predicted protein | 6.0 | 219.2 | * | - |
|  | PAAG_06624 | predicted protein | 5.0 | 275.6 | * | - |
|  | PAAG_01919 | predicted protein | 5.0 | 310.1 | * | - |
|  | PAAG_00183 | predicted protein | 7.0 | 274.0 | * | - |
|  | PAAG_04936 | predicted protein | 3.0 | 615.9 | * | - |
|  | PAAG_04967 | predicted protein | 1.0 | 422.4 | * | - |
|  | PAAG_00251 | hypothetical protein | 3.0 | 361.8 | * | - |

^a^ Identification of differentially regulated proteins from *Paracoccidioides* genome database (<http://www.broadinstitute.org/annotation/genome/paracoccidioides_brasiliensis/MultiHome.html>) using the ProteinLynx Global Server (PLGS) version 3.0 (Waters Corporation. Manchester. UK);

^b^ Proteins annotation from *Paracoccidioides* genome database or by homology in NCBI database (<http://www.ncbi.nlm.nih.gov/>);

^c^  Average of matching peptides and score for each protein obtained from MS data using the ProteinLynx Global Server (PLGS);

^d^ Protein expression profiles in log2 (fold change) obtained from ProteinLynx Global Server (PLGS) analysis normalized with internal standard.

^e^ Biological process of differentially expressed proteins from MIPS

(<http://pedant.helmholtz-muenchen.de/pedant3htmlview/pedant3view?Method=analysis&Db=p3_r48325_Par_brasi_Pb01> ) and Uniprot database (<http://www.uniprot.org/>).

*: identified only in the presence of glucose (carbon condition).
