# Supplementary material for: Transcriptional and Proteomic Responses to Carbon Starvation in Paracoccidioides
Source: PLoS Negl Trop Dis. 2014 May 8;8(5):e2855. doi: 10.1371/journal.pntd.0002855 (PMC4014450; doi:10.1371/journal.pntd.0002855)
Supplement: Table S7 — Up-regulated proteins and transcripts of Paracoccidioides ( Pb 01) yeast cells under carbon starvation detected by NanoUPLC-MSE and RNAseq analysis. (DOC) [file pntd.0002855.s018.doc]

**Table S7. Up-regulated proteins and transcripts of *Paracoccidioides* (*Pb*01) yeast cells under carbon starvation detected by NanoUPLC-MS^E^ and RNAseq analysis.**

|  | **ID^a^** | **Annotation^b^** | **Fold change (proteome)^c^** | **Fold change (transcriptome)^d^** | **Biological process^e^** |
| --- | --- | --- | --- | --- | --- |
| **METABOLISM** | | | | | |
| **Amino acid metabolism** | | | | | |
|  | PAAG_05253 | delta-1-pyrroline-5-carboxylate dehydrogenase | 0.87 | 1.26 | glutamate degradation |
|  | PAAG_00966 | L-threonine 3-dehydrogenase | 0.74 | 1.20 | threonine degradation |
|  | PAAG_08162 | maleylacetoacetate isomerase | # | 1.35 | phenylalanine degradation |
|  | PAAG_08649 | cysteine dioxygenase | # | 1.15 | cysteine degradation |
|  | PAAG_01365 | choline dehydrogenase | # | 1.64 | glycine biosynthesis |
| **Nitrogen and sulfur metabolism** | | | | | |
|  | PAAG_03333 | formamidase | 2.05 | 4.39 | nitrogen metabolism |
| **C-compound and carbohydrate metabolism** | | | | | |
|  | PAAG_02162 | lactam utilization protein LamB | # | 2.54 | C-compound and carbohydrate metabolism |
|  | PAAG_02653 | acetyl-coenzyme A synthetase | # | 1.67 | C-compound and carbohydrate metabolism |
|  | PAAG_03765 | NADP-dependent glycerol dehydrogenase | # | 2.53 | sugar. glucoside. polyol and carboxylate catabolism |
|  | PAAG_05416 | NADP-dependent leukotriene B4 12-hydroxydehydrogenase | # | 2.43 | C-compound and carbohydrate metabolism |
| **Lipid, fatty acid and isoprenoid metabolism** | | | | | |
|  | PAAG_02664 | 3-ketoacyl-CoA thiolase | 1.15 | 1.77 | lipid and fatty acid metabolism_beta oxidation |
|  | PAAG_05249 | aldehyde dehydrogenase | 2.45 | 2.42 | lipid and fatty acid metabolism_oxidation |
|  | PAAG_02163 | acetyl-/propionyl-coenzyme A carboxylase alpha chain | 3.03 | 2.09 | lipid metabolism |
|  | PAAG_01928 | peroxisomal dehydratase | # | 1.93 | Lipid, fatty acid and isoprenoid metabolism |
| **Secundary metabolism** |  |  |  |  |  |
|  | PAAG_02336 | nudix hydrolase | 1.27 | 1.07 | metabolism of vitamins, cofactors and prosthetic groups |
|  | PAAG_08856 | nicotinate-nucleotide pyrophosphorylase | # | 1.20 | biosynthesis of vitamins, cofactors and prosthetic groups |
| **ENERGY** | | | | | |
| **Glycolysis and gluconeogenesis** | | | | | |
|  | PAAG_01995 | fructose-bisphosphate aldolase | 1.30 | 1.28 | glycolysis and gluconeogenesis |
| **Ethanol production** | | | | | |
|  | PAAG_00403 | alcohol dehydrogenase | 1.83 | 3.92 | alcohol fermentation |
|  | PAAG_04541 | alcohol dehydrogenase | 0.97 | 2.77 | alcohol fermentation |
|  | PAAG_02512 | pyruvate decarboxylase | # | 1.33 | alcohol fermentation |
|  | PAAG_07038 | APAF1-interacting protein | # | 1.24 | metal binding |
| **CELL RESCUE, DEFENSE AND VIRULENCE** | | | | | |
|  | PAAG_02926 | superoxide dismutase | # | 2.49 | detoxification |
|  | PAAG_08277 | nitroreductase family protein | # | 1.30 | detoxification |
|  | PAAG_03502 | cytochrome c peroxidase | # | 3.03 | oxidative stress response |
|  | PAAG_02548 | hydroxyacylglutathione hydrolase | # | 1.03 | glutathione biosynthetic process/ stress response |
| **CELL GROWTH/ MORPHOGENESIS** | | | | | |
|  | PAAG_03624 | Arp2/3 complex subunit Arc16 | # | 2.46 | cell growth/morphogenesis |
| **UNCLASSIFIED** | | | | | |
|  | PAAG_06083 | dienelactone hydrolase family protein | 1.40 | 3.34 | - |
|  | PAAG_00297 | conserved hypothetical protein | 1.37 | 1.19 | - |
|  | PAAG_01254 | predicted protein | # | 1.24 | - |
|  | PAAG_01455 | hypothetical protein | # | 3.07 | - |
|  | PAAG_02985 | hypothetical protein | # | 1.24 | - |
|  | PAAG_05856 | conserved hypothetical protein | # | 1.21 | - |

^a^ Identification of **the same** proteins and transcripts which were regulated in proteome and transcriptome analysis from *Paracoccidioides* genome database (<http://www.broadinstitute.org/annotation/genome/paracoccidioides_brasiliensis/MultiHome.html>);

^b^ Proteins and transcripts annotations from *Paracoccidioides* genome database or by homology in NCBI database (<http://www.ncbi.nlm.nih.gov/>);

^c^ Protein expression profiles in log2 (fold change) obtained from ProteinLynx Global Server (PLGS) analysis normalized with internal standard.

^d^ Transcript expression profiles in log2 (fold change) obtained from fold change selection method for differentially expressed transcripts using a Fisher exact test with a p-value of 0.001.

^e^ Biological process of differentially expressed transcripts and proteins from MIPS

(<http://pedant.helmholtz-muenchen.de/pedant3htmlview/pedant3view?Method=analysis&Db=p3_r48325_Par_brasi_Pb01>) and Uniprot database (<http://www.uniprot.org/>).

**#:** identified only in carbon starvation condition.
