# Supplementary material for: Transcriptional and Proteomic Responses to Carbon Starvation in Paracoccidioides
Source: PLoS Negl Trop Dis. 2014 May 8;8(5):e2855. doi: 10.1371/journal.pntd.0002855 (PMC4014450; doi:10.1371/journal.pntd.0002855)
Supplement: Table S8 — Down-regulated proteins and transcripts of Paracoccidioides ( Pb 01) yeast cells under carbon starvation detected by NanoUPLC-MSE and RNAseq analysis. (DOC) [file pntd.0002855.s019.doc]

**Table S8. Down-regulated proteins and transcripts of *Paracoccidioides* (*Pb*01) yeast cells under carbon starvation detected by NanoUPLC-MS^E^ and RNAseq analysis.**

|  | **ID^a^** | **Annotation^b^** | **Fold change (proteome)^c^** | **Fold change (transcriptome)^d^** | **Biological process^e^** |
| --- | --- | --- | --- | --- | --- |
| **METABOLISM** | | | | | |
| **Amino acid metabolism** | | | | | |
|  |  |  |  |  |  |
|  | PAAG_07102 | pentafunctional AROM polypeptide | * | -1.83 | aromatic group biosynthesis |
|  | PAAG_05929 | sulfate adenylyltransferase | * | -2.74 | cysteine and methionine biosynthesis |
|  | PAAG_07813 | cysteine synthase | * | -1.34 | cysteine biosynthesis |
|  | PAAG_05328 | 3-isopropylmalate dehydrogenase A | * | -1.54 | leucine biosynthesis |
|  |  |  |  |  |  |
| **C-compound and carbohydrate metabolism** | | | | | |
|  | PAAG_00545 | glycogen phosphorylase | -1.47 | -1.68 | carbohydrate metabolism |
|  | PAAG_02769 | pyruvate dehydrogenase protein X component | * | -0.94 | carbohydrate metabolism |
|  | | | | | |
|  |  |  |  |  |  |
| **Purin nucleotide/ nucleoside/ nucleobase metabolism** | | | | | |
|  | PAAG_06906 | adenine phosphoribosyltransferase | * | -1.24 | purin nucleotide/nucleoside/nucleobase metabolism |
| **ENERGY** | | | | | |
| **Electron transport and membrane-associated energy conservation** | | | | | |
|  | PAAG_00953 | NADH-cytochrome b5 reductase | -0.59 | -1.06 | electron transport |
| **CELL RESCUE, DEFENSE AND VIRULENCE** | | | | | |
|  | PAAG_03216 | mitochondrial peroxiredoxin PRX1 | * | -3.50 | oxidative stress response |
|  | PAAG_01465 | carbonic anhydrase | * | -3.66 | stress oxidative response/ carbon utilization |
| **UNCLASSIFIED** | | | | | |
|  | PAAG_03152 | CobW domain-containing protein | -1.31 | -1.55 | - |
|  | PAAG_07772 | conserved hypothetical protein | -0.69 | -1.70 | - |
|  | PAAG_08103 | EF hand domain-containing protein | * | -1.00 | - |
|  | PAAG_00251 | hypothetical protein | * | -2.70 | - |
|  | PAAG_04793 | LEA domain-containing protein | * | -1.76 | - |
|  | PAAG_05037 | HHE domain-containing protein | * | -6.18 | - |
|  | PAAG_05181 | conserved leucine-rich repeat protein | * | -1.70 | - |

^a^ Identification of **the same** proteins and transcripts which were regulated in proteome and transcriptome analysis from *Paracoccidioides* genome database (<http://www.broadinstitute.org/annotation/genome/paracoccidioides_brasiliensis/MultiHome.html>);

^b^ Proteins and transcripts annotations from *Paracoccidioides* genome database or by homology in NCBI database (<http://www.ncbi.nlm.nih.gov/>);

^c^ Protein expression profiles in log2 (fold change) obtained from ProteinLynx Global Server (PLGS) analysis normalized with internal standard.

^d^ Transcript expression profiles in log2 (fold change) obtained from fold change selection method for differentially expressed transcripts using a Fisher exact test with a p-value of 0.001.

^e^ Biological process of differentially expressed transcripts and proteins from MIPS

(<http://pedant.helmholtz-muenchen.de/pedant3htmlview/pedant3view?Method=analysis&Db=p3_r48325_Par_brasi_Pb01>) and Uniprot database (<http://www.uniprot.org/>).

***:** identified only in the presence of glucose (carbon condition).
