# Supplementary material for: Transcriptional and Proteomic Responses to Carbon Starvation in Paracoccidioides
Source: PLoS Negl Trop Dis. 2014 May 8;8(5):e2855. doi: 10.1371/journal.pntd.0002855 (PMC4014450; doi:10.1371/journal.pntd.0002855)
Supplement: Table S9 — Up- and down-regulated proteins and transcripts, respectively, of Paracoccidioides ( Pb 01) yeast cells under carbon starvation detected by NanoUPLC-MSE and RNAseq analysis. (DOC) [file pntd.0002855.s020.doc]

**Table S9. Up- and down-regulated proteins and transcripts, respectively, of *Paracoccidioides* (*Pb*01) yeast cells under carbon starvation detected by NanoUPLC-MS^E^ and RNAseq analysis.**

|  | **ID^a^** | **Annotation^b^** | **Fold change (proteome)^c^** | **Fold change (transcriptome)^d^** | **Biological process^e^** |
| --- | --- | --- | --- | --- | --- |
| **METABOLISM** | | | | | |
| **Amino acid metabolism** | | | | | |
|  | PAAG_07998 | glutamate synthase small chain | # | -1.54 | glutamate biosynthesis |
|  | PAAG_02901 | S-adenosylmethionine synthetase | # | -1.38 | L-methionine biosynthesis |
| **PROTEIN FATE** | | | | | |
|  | PAAG_04555 | sarcosine oxidase | # | -1.20 | protein modification |
| **TRANSPORT** | | | | | |
|  | PAAG_03577 | ABC drug exporter AtrF | # | -1.89 | drug/ toxin transport |
| **MISCELLANEOUS** | | | | | |
|  | PAAG_03233 | oxidoreductase | # | -1.15 | oxidation-reduction process |
| **UNCLASSIFIED** | | | | | |
|  | PAAG_02242 | hypothetical protein | # | -1.77 | - |

^a^ Identification of **the same** proteins and transcripts which were regulated in proteome and transcriptome analysis from *Paracoccidioides* genome database (<http://www.broadinstitute.org/annotation/genome/paracoccidioides_brasiliensis/MultiHome.html>);

^b^ Proteins and transcripts annotations from *Paracoccidioides* genome database or by homology in NCBI database (<http://www.ncbi.nlm.nih.gov/>);

^c^ Protein expression profiles in log2 (fold change) obtained from ProteinLynx Global Server (PLGS) analysis normalized with internal standard.

^d^ Transcript expression profiles in log2 (fold change) obtained from fold change selection method for differentially expressed transcripts using a Fisher exact test with a p-value of 0.001.

^e^ Biological process of differentially expressed transcripts and proteins from MIPS (<http://pedant.helmholtz-muenchen.de/pedant3htmlview/pedant3view?Method=analysis&Db=p3_r48325_Par_brasi_Pb01>) and Uniprot database (<http://www.uniprot.org/>).

**#:** identified only in carbon starvation condition.
