# Supplementary material for: Transcriptional and Proteomic Responses to Carbon Starvation in Paracoccidioides
Source: PLoS Negl Trop Dis. 2014 May 8;8(5):e2855. doi: 10.1371/journal.pntd.0002855 (PMC4014450; doi:10.1371/journal.pntd.0002855)
Supplement: Table S10 — Down- and up-regulated proteins and transcripts, respectively, of Paracoccidioides ( Pb 01) yeast cells under carbon starvation detected by NanoUPLC-MSE and RNAseq analysis. (DOC) [file pntd.0002855.s021.doc]

**Table S10. Down- and up-regulated proteins and transcripts, respectively, of *Paracoccidioides* (*Pb*01) yeast cells under carbon starvation detected by NanoUPLC-MS^E^ and RNAseq analysis.**

|  | **ID^a^** | **Annotation^b^** | **Fold change (proteome)^c^** | **Fold change (transcriptome)^d^** | **Biological process^e^** |
| --- | --- | --- | --- | --- | --- |
| **UNCLASSIFIED** | | | | | |
|  | PAAG_00503 | HAD-superfamily hydrolase | -0.78 | 1.41 | - |
|  | PAAG_06624 | predicted protein | * | 1.41 | - |

^a^ Identification of **the same** proteins and transcripts which were regulated in proteome and transcriptome analysis from *Paracoccidioides* genome database (<http://www.broadinstitute.org/annotation/genome/paracoccidioides_brasiliensis/MultiHome.html>);

^b^ Proteins and transcripts annotations from *Paracoccidioides* genome database or by homology in NCBI database (<http://www.ncbi.nlm.nih.gov/>);

^c^ Protein expression profiles in log2 (fold change) obtained from ProteinLynx Global Server (PLGS) analysis normalized with internal standard.

^d^ Transcript expression profiles in log2 (fold change) obtained from fold change selection method for differentially expressed transcripts using a Fisher exact test with a p-value of 0.001.

^e^ Biological process of differentially expressed transcripts and proteins from MIPS

(<http://pedant.helmholtz-muenchen.de/pedant3htmlview/pedant3view?Method=analysis&Db=p3_r48325_Par_brasi_Pb01>) and Uniprot database (<http://www.uniprot.org/>).

***:** identified only in the presence of glucose (carbon condition).
